# Discovery of an L‐like Configuration for 3’‐Fluoro‐5’‐norcarbonucleoside Phosphonates as Potent Anti‐HIV Agents

**DOI:** 10.1002/cmdc.202200377

**Published:** 2022-09-15

**Authors:** Pierre‐Yves Geant, Malika Kaci, Jean‐Pierre Uttaro, Christian Périgaud, Christophe Mathé

**Affiliations:** ^1^ Institut des Biomolécules Max Mousseron (IBMM) UMR 5247 Université de Montpellier, CNRS, ENSCM 1019, route de Mende 34293 Montpellier France

**Keywords:** antiviral, HIV, L-enantiomer, nucleoside, phosphonate

## Abstract

Recently, we reported the racemic synthesis of 3’‐fluoro‐5’‐norcarbocyclic nucleoside phosphonates bearing adenine as the heterocyclic base. For this study, to evaluate the antiviral activity of each enantiomer, we synthesized both enantiomers, as well as their corresponding bis(POM) prodrugs. Anti‐HIV‐1 evaluation against the LAI strain and clinically NRTI‐resistant HIV‐1 strains are presented. The activities against these different strains show that the activities of bis(POM) prodrug (−)‐**9** are equivalent or even superior to those of (*R)‐*PMPA.

## Graphical abstract

**We recently reported** the racemic synthesis of 3’‐fluoro‐5’‐norcarbocyclic nucleoside phosphonates bearing adenine as heterocyclic base. To evaluate the antiviral activity of each enantiomer, we synthesized both enantiomers as well as their corresponding bis(POM) prodrugs. Evaluations against the HIV‐1 LAI strain and clinically NRTI‐resistant HIV‐1 strains are presented. The activities against these different strains show that the activities of bis(POM) prodrug (−)‐**9** are equivalent or even superior to those of (*R)‐*PMPA.

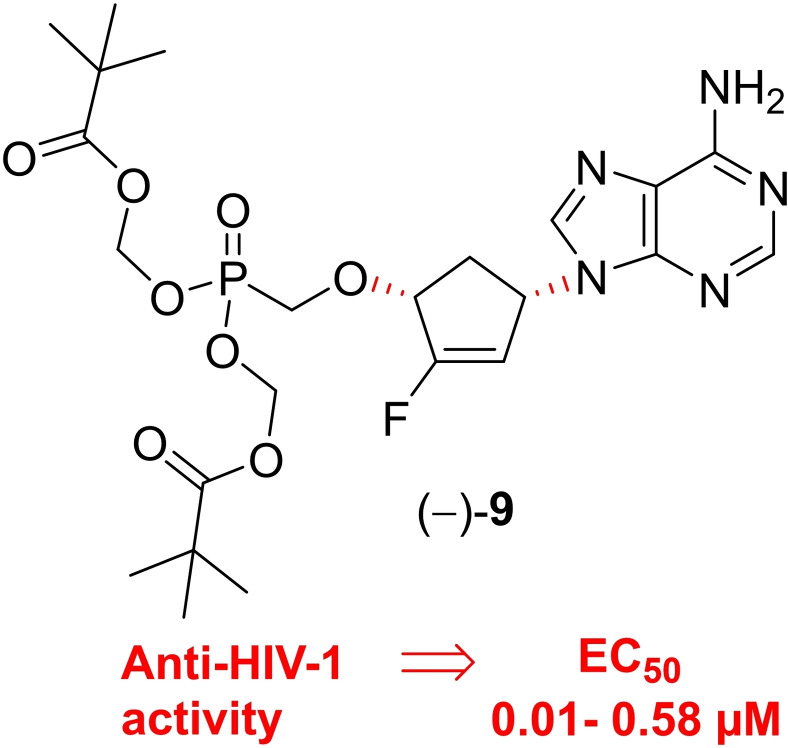

## Introduction

**Figure 1:**
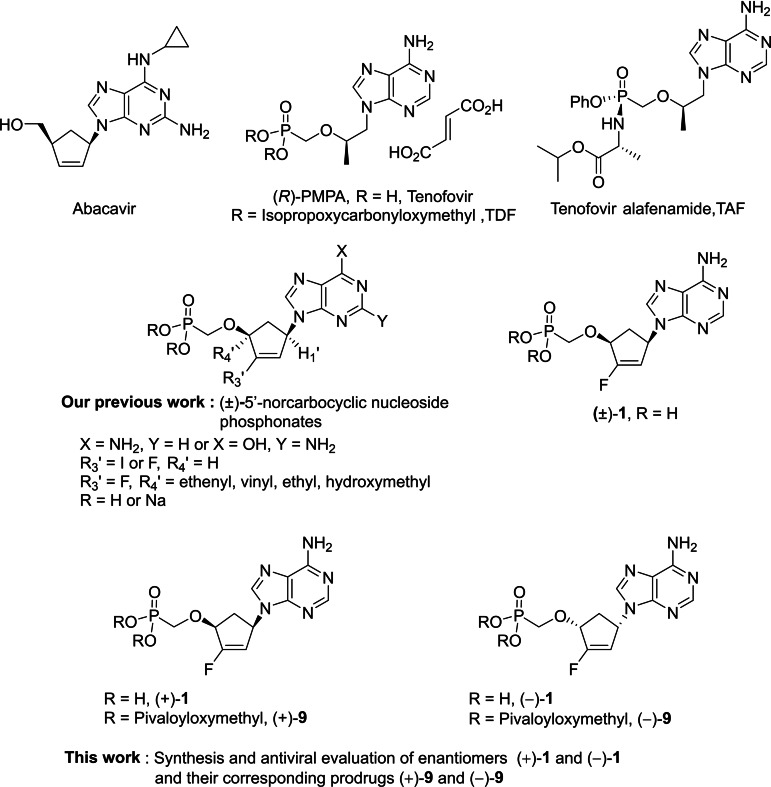
Chemical structures of Abacavir, Tenofovir and its prodrugs TDF and TAF. Structures of the 5’‐norcarbocyclic nucleotides previously described as well as compounds investigated in this study.

In this period of the Covid‐19 pandemic that we are going through, impacting our model of society and leading us somehow to dehumanize ourselves, we should not forget that other pandemics remain present. The year 2021 was the 40th sad anniversary of the onset of the Human Immunodeficiency Virus (HIV)/Acquired Immunodeficiency Syndrome (AIDS) pandemic.^[1]^ Around 38 millions of people are currently living with HIV infections worldwide and most of them would die without any long life antiretroviral treatment. Four decades after the discovery of HIV as the etiological agent of AIDS, no vaccine is still available for the prevention of AIDS and antivirals are the only suitable weapons to treat HIV infections. It is a crucial concern because all anti‐HIV drugs have long‐term side effects and may be associated with the emergence of resistant viral strains, in the event of poor or sub‐optimal treatment compliance, especially in countries where access to anti‐HIV medicines or antiretroviral therapy (ART) is structurally underdeveloped. Therefore, it is essential to propose new anti‐HIV agents in order to find out molecules that are more effective and less toxic than those currently used. Among the compounds approved by the Food and Drug Administration (FDA) as anti‐HIV agents, nucleosides and nucleotides reverse transcriptase inhibitors (NRTI)^[2]^ represent an important family, as illustrated with the clinical use of Abacavir, Tenofovir disoproxil fumarate (TDF) and Tenofovir alafenamide (TAF) (Figure 1).^[3]^ As a part of our research on carbocyclic nucleoside phosphonates as potential anti‐viral agents, we have reported several series of 5’‐norcarbocyclic nucleoside phosphonates as potent anti‐HIV agents.^[4]^ They combine different structural modifications already present in the development of antiretroviral agents such as the replacement of the oxygen atom of the sugar moiety by a methylene group, the introduction of a phosphonyl moiety as mimic of the phosphate function as well as various additional modifications in positions 3’ or 4’ (Figure 1, R_3_’=I or F, R_4_’=ethenyl, vinyl…). In such a carbonucleoside structure, isosteric O/CH_2_ replacement confers chemical and metabolic stability compared to pentofuranonucleoside analogues. In the case of nucleoside phosphonates, the presence of P−C bond instead of the hydrolysable P−O bound gives rise to a metabolic stable linkage in comparison to the phosphate moiety.^[5]^

Among the previously synthetized compounds, belonging to 5’‐norcarbocyclic nucleoside phosphonates, compound ((±)‐**1**, Figure 1) exhibited significant anti‐HIV‐1 activity against LAI strain in PBMC (EC_50_=0.88 μM), comparatively with (*R)‐*PMPA (Tenofovir) (EC_50_=1.1 μM) and without additional cytotoxicity.^[4b]^

Nevertheless, compound **(±)‐1** has been synthesized and evaluated as a racemic form. In order to evaluate the antiviral activity of each enantiomer, we describe herein the synthesis and biological evaluation of both enantiomers (+)**‐1** and (−)**‐1** and their corresponding bis(PivaloylOxyMethyl) (POM) prodrugs, (+)**‐9** and (−)**‐9**.

## Results and Discussion

### Chemistry

**Scheme 1:**
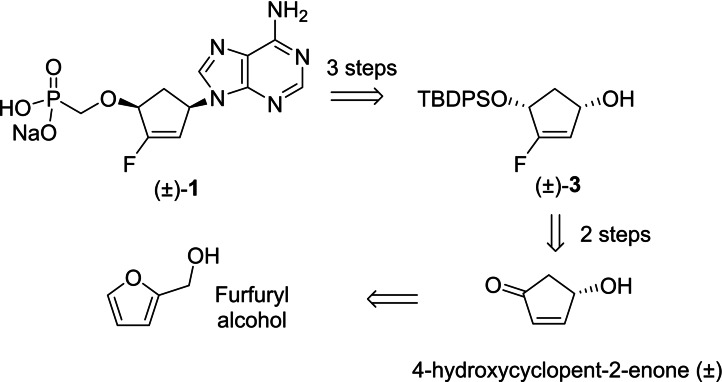
Retrosynthetic pathway used for the racemic synthesis of compound (±)‐**1**.^[4b]^

In our previous work,^[4b]^ we achieved the racemic synthesis of compound **1** using 4‐hydroxycyclopent‐2‐enone (±) as intermediate (Scheme 1). Such a compound is a suitable molecular scaffold, as an enantiopure form, for the development of important chiral building blocks in chemical synthesis from racemic preparations, enantioselective approaches, syntheses from the chiral pool.^[6]^ Close to the structure of this scaffold, the preparation of optically active *cis*‐4‐*O*‐protected‐cyclopentene‐2‐enol derivatives raised also a great interest because of their potential use as chiral building blocks. Many examples in the literature report their use as a chiral precursor for the enantioselective synthesis of carbocyclic nucleosides,^[7]^ total synthesis of natural products^[8]^ and prostaglandin derivatives.^[9]^ Previously reported preparations of enantiomeric pure cis‐2‐cyclopentene‐1,4‐diol employ enzymatic resolutions of 4‐*O*‐protected derivatives^[10]^ or chemical desymmetrization of meso‐cyclopentenoids using a palladium complex of the Trost ligand.^[6]^ However, these procedures are not necessarily user‐friendly. They require the use of biocatalysts or catalysts that can be expensive, time consuming and difficult to implement on a large scale in order to obtain substantial amounts of chiral building blocks. In order to quickly and easily access to a chiral building blocks of interest, we focused our efforts on an alternative approach based on the deracemization on a more advanced intermediate of the synthesis of compound (±)‐**1**, namely compound (±)‐**3** (Scheme 1).

**Scheme 2:**
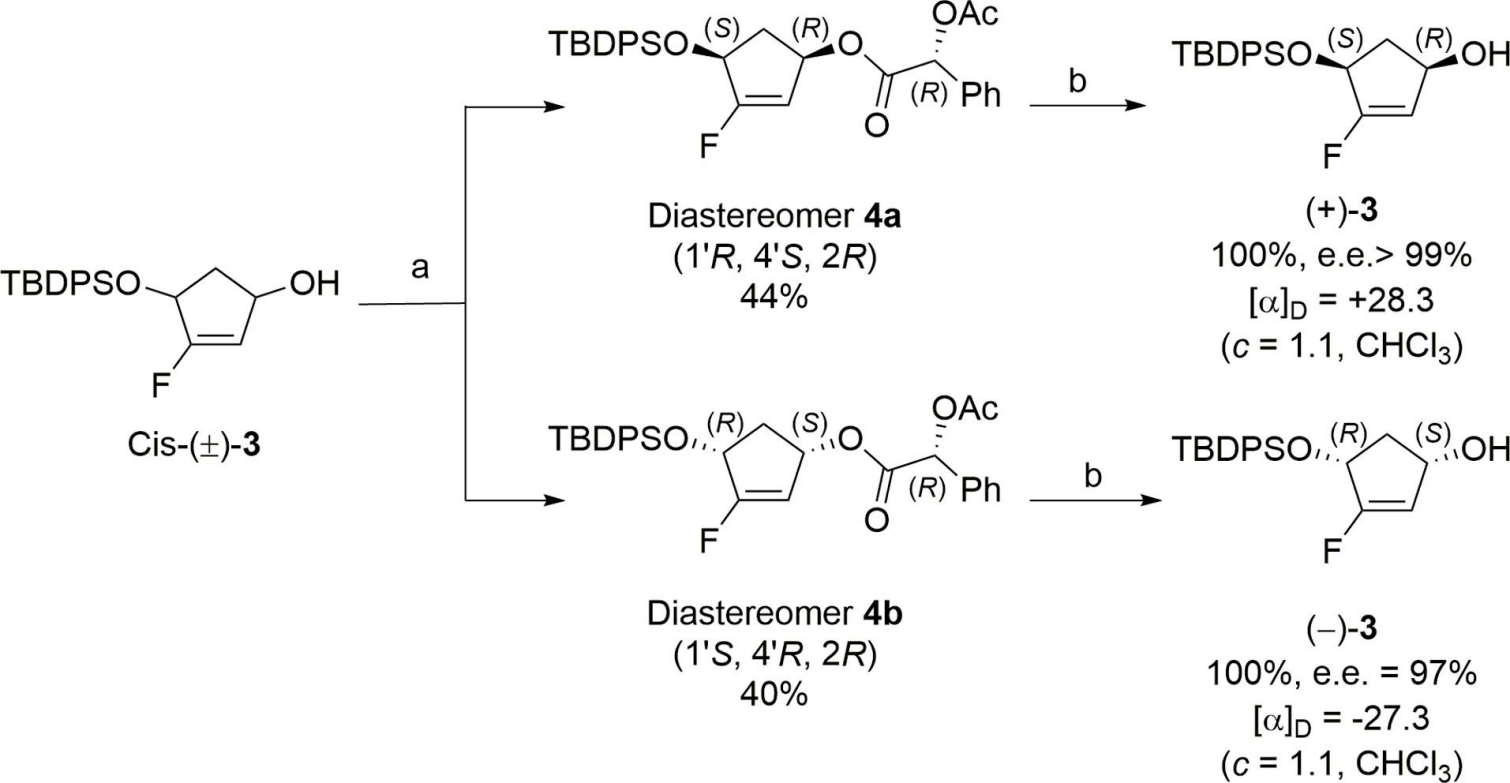
Optical resolution of (±)‐**3** with (*R)‐O*‐acetylmandelic acid. Reagents and conditions: a) i) (*R)‐O*‐acetylmandelic acid, DCC, DMAP, CH_2_Cl_2_, rt, 2 h; ii) silica gel chromatography; b) LiOH, THF/H_2_O, rt, 4 h.

Among the methods developed for optical resolution, we chose to introduce resolving agents^[11]^ on compound (±)‐**3** (Scheme 2). The presence of the allylic hydroxyl function can be helpful for its conversion into the corresponding ester using one of the enantiomers of a chiral carboxylic acid. Thus, many chiral acids can be used to perform optical resolution. Among the common resolving agents, we considered the (*R)‐O*‐acetylmandelic acid and DCC/DMAP mediated coupling^[12]^ (Steglich esterification) was chosen as convenient method for the introduction of the resolving agent.

**Figure 2:**
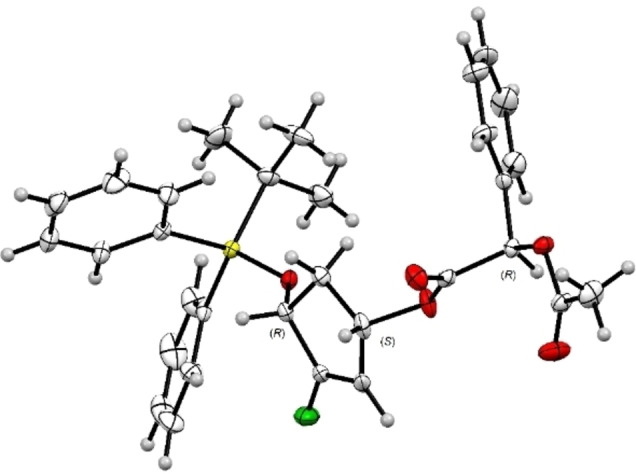
ORTEP drawing of the X‐ray crystallographic structure of **4 b**.

Under these conditions, diastereomers **4 a** and **4 b** were obtained after separation on silica gel chromatography in 44 % and 40 % yield, respectively. The crystallization allows us to obtain a crystal of **4 b** and its structure was unambiguously determined by X‐ray crystallographic analysis. The absolute stereochemistry (1’*S*, 4’*R*, 2*R*) of diastereomer **4 b** was established as depicted in Figure 2.

**Scheme 3:**
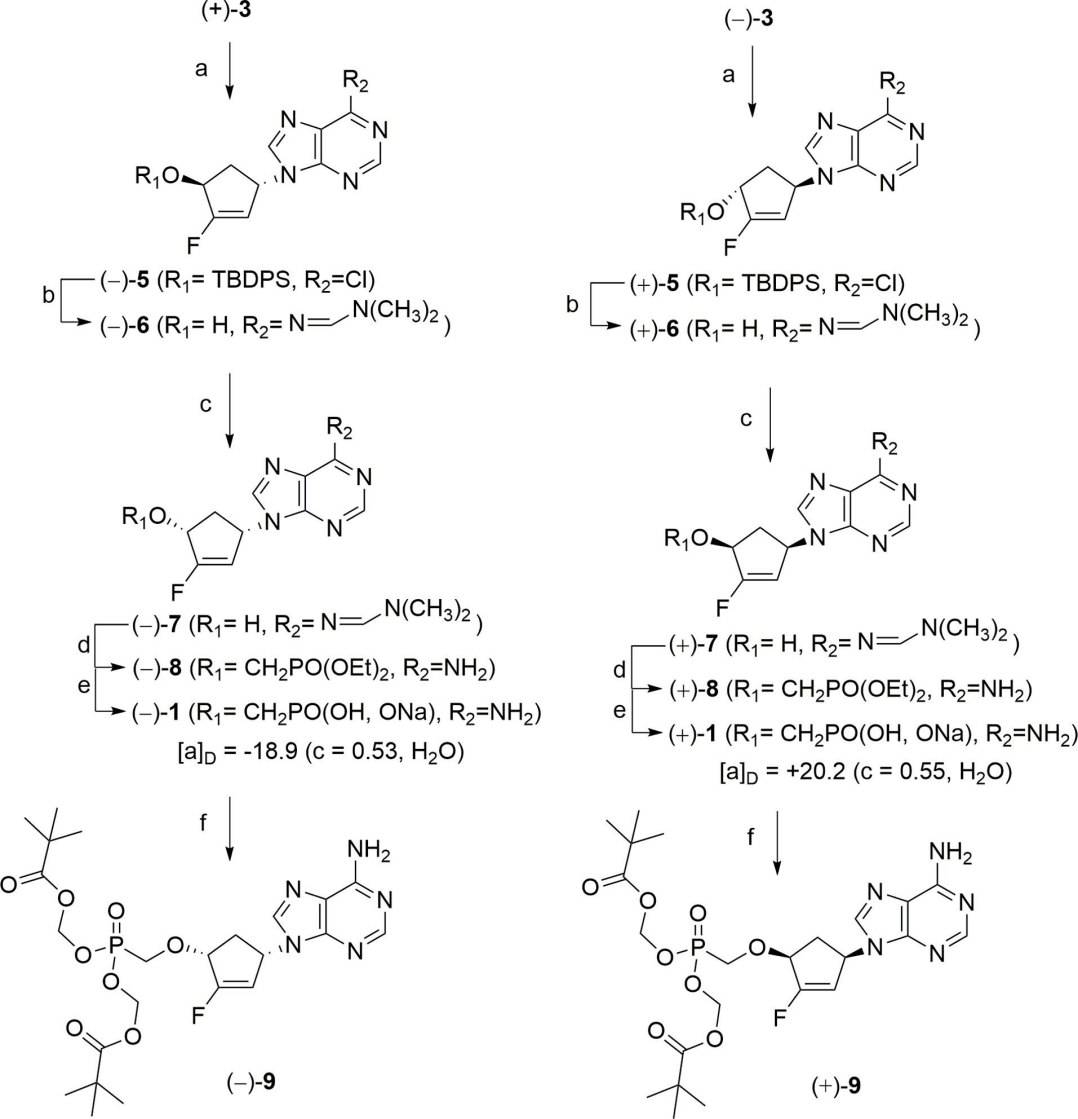
Synthesis of enantiomers of compound **1** and their corresponding bis(POM) prodrugs. Reagents and conditions: a) 6‐chloropurine, PPh_3_, DIAD, THF, rt, 1 h; b) i) NH_3_/MeOH, 70 °C, 24 h; ii) DMF‐dimethylacetal, DMF, 50 °C, 15 h; c) i) benzoic acid, PPh_3_, DIAD, THF, rt, 3 h; ii) K_2_CO_3_, MeOH, rt, 2 h; d) i) (EtO)_2_POCH_2_OTs, LiOtBu, THF, rt, 3 days; ii) AcOH/MeOH/H_2_O, rt, 24 h; e) TMSBr, DMF, rt, 16 h; f) i) Bu_4_NOH, Et_3_N, DMF, rt, 30 min; ii) POMCl, rt, 5 days.

At this stage, the optical resolving agent must be removed to obtain the two desired enantiomers of compound **3**. Due to the presence of the TBDPS group, the removal was carried out in basic conditions. At first, we used MeONa in MeOH but the purification step was difficult due to the co‐elution of the methyl mandelate formed under these conditions with compound **3**. In order to improve the yield and to simplify the purification step, the reaction was carried out using LiOH in a THF/H_2_O mixture (1/1; v/v). Under these conditions, the side product formed is mandelic acid, a more polar compound than the corresponding methyl ester. In this case, mandelic acid was easily removed and both enantiomers of compound **3** were obtained in quantitative yield (Scheme 2). The optical purity of each enantiomer was determined by HPLC analysis (see SI), based on racemic sample, and was established as more than 99 % for the dextrogyre compound and 97 % for the levogyre enantiomer. This optical resolution, which is efficient and very easy to implement, allowed us to obtain both enantiomers of the desired compound **3** in gram scale. With these last in hand, the synthesis of both enantiomers of derivative **1** was carried out following our previously reported multi‐steps approach and briefly summarized in Scheme 3. As expected, compound (+)‐**3** gives the levogyre enantiomer of compound **1** whereas compound (−)‐**3** furnished the dextrogyre enantiomer of compound **1**.

However, these two enantiomers can suffer from limitations such as their reduced cell penetration (the free phosphonic acid form is negatively charged at physiological pH). Finally, we decided to synthesize the most commonly used carbonyloxymethyl pronucleotides (pivaloyloxymethyl‐ or POM)^[13]^ prodrug forms of both enantiomers of compound **1**. Indeed, the POM group, previously reported as a basic labile protecting group^[14]^ does not introduce an additional stereochemical center at phosphorus atom. Thus, each enantiomers of compound **1** was first converted into the corresponding tetrabutylammonium salt using tetrabutylammonium hydroxide, in order to improve compound solubility in organic solvents, and was then treated with NEt_3_ in dry DMF in the presence of chloromethyl pivalate to furnished the desired bis(POM) products, (−)**‐9** and (+)**‐9**, as described in Scheme 3.

### Anti‐HIV evaluation

The newly synthesized phosphonate compounds, which can be seen as pairs of D‐ and L‐like carbonucleoside enantiomers, were evaluated in PBM cells for their activity against the replication of HIV‐1 LAI strain as well as their cytotoxicity, comparatively with (±)‐**1** and (*R)‐*PMPA (Tenofovir). The results are presented in Table 1. These evaluations showed that the L‐like enantiomer (−)‐**1** exhibited a more potent anti‐HIV activity (EC_50_=0.19 μM) than the racemic (±)‐**1** as previously reported. The antiviral activity observed for (−)‐**1** was higher than that of Tenofovir (EC_50_=1.1 μM). The nucleoside phosphonate D‐like analogue **(+)‐1** exhibited a lower activity (EC_50_=4.3 μM) in comparison with the L‐like enantiomer as well as Tenofovir. No cytotoxicity was observed for both enantiomers (−)‐**1** and (+)**‐1**. The anti‐HIV evaluation of their corresponding bis(POM) prodrugs exhibited, as expected, a better antiviral activity for both enantiomers comparatively to their parent nucleoside. Remarkably, the anti‐HIV‐1 activity of the corresponding L‐type enantiomer (EC_50_=0.063 μM) was 8‐fold greater than that of the D‐like and 17‐fold greater than Tenofovir (−)‐**9**, however associated with some cytotoxicity, without compromising the selectivity index (SI) which remains much higher as compared with Tenofovir (82.5 versus 9.1).

**Table 1:** Anti HIV‐1 LAI activity and cytotoxic properties of 3’‐fluoro‐5’‐norcarbocyclic nucleoside phosphonates and reference compound (*R)‐*PMPA in human peripheral blood mononuclear cells (PBMC).

Compounds	EC_50_ [μM]^[a]^	CC_50_ [μM]^[b]^	SI^[c]^
(±)**‐1** ^[d]^	0.88	≥10	≥1.3
(−)‐**1**	0.19	≥10	≥52.6
(+)**‐1**	4.3	≥10	≥2.32
(−)**‐9**	0.063	5.2	≥82.5
(+)**‐9**	0.51	≥10	≥10.1
**(*R)‐*PMPA** ^[d]^	1.1	≥10	≥9.1

[a] EC_50_ Effective concentration of drug required to reduce viral replication by 50 %. Data are the mean from 3 blood donors. [b] CC_50_ Cytotoxic concentration of drug required to reduce cell growth by 50 %. Data are the mean from 3 blood donors. [c] SI=CC_50_/EC_50_. [d] Reference^[4b]^

One of the drawbacks of the use of NRTI is the emergence of drug‐resistant strains during the extended treatment period, which may significantly compromise the clinical efficacy. On the basis of the results obtained, we selected compounds (±)‐1, (−)‐1, (−)‐9 and (+)‐9 and Tenofovir in order to determine their anti‐HIV activities against LAI, as a reference strain, in T CD4+ H9 cells as well as resistant strains to compounds used in chemotherapy, owing to mutations at the level of reverse transcriptase. Testing the activity of the selected compounds against drug‐resistant strains will establish their relative activity against clinically relevant NRTI‐resistant viruses. The selected drug‐resistant viral strains were HIV‐1‐56252‐1, HIV‐1‐7324‐1, HIV‐1‐52534‐2 and HIV‐1‐71361‐1. They were selected according to the structure of the most effective compounds of our work with degrees of resistance towards nucleosides used in anti‐HIV chemotherapy, such as ABC and TDF, and nucleoside analogues of the L series, such as 3TC. The corresponding mutations which confer resistance to NRTI have been previously identified (TAM, K65R, and/or insertion in position 69, etc.…).^[15]^ The viral strains HIV‐1‐56252‐1 and HIV‐1‐52534‐2 are strongly resistant to AZT, TDF, ABC and 3TC, respectively. The strain HIV‐1‐7324‐1 is strongly resistant to AZT but less resistant to TDF and ABC. Whereas, the strain HIV‐1‐71361‐1 is AZT‐sensitive but slightly less resistant to TDF, ABC and 3TC.^[16]^ The results are presented in Table 2. Among the most drug‐resistant viral strains (i. e. HIV‐1‐52534‐2), no activity was observed for the 3’‐fluoro‐5’‐norcabocyclic nucleoside phosphonates except for compound (−)‐9 which remains significantly active against strain 52534‐2. Antiviral activity was also maintained against resistant strains 56252‐1, 7324‐1 and 71631‐1, with a better antiviral effect for compounds with a L‐like configuration (i. e., (−)‐1 and (−)‐9). Such a difference in activity between a D‐ or L‐like configuration nucleoside analogues has been already reported in the literature.^[17]^ Herein, we report for the first time a significant anti‐HIV activity of a 5’‐norcarbocyclic nucleoside^[18]^ structure that is associated with an L‐like configuration.

**Table 2:** Anti HIV‐1 activity against resistant viral strains of selected 3’‐fluoro‐5’‐norcarbocyclic nucleoside phosphonates and their prodrugs and reference compound (*R)‐*PMPA in T CD4+ H9 cells.

	Strains				
	LAI	56252‐1	7324‐1	52534‐2	71361‐1
Compounds	EC_50_ [μM]^[a]^	EC_50_ [μM]^[a]^	EC_50_ [μM]^[a]^	EC_50_ [μM]^[a]^	EC_50_ [μM]^[a]^
(±)**‐1**	0.66	10	0.17	≥10	3.73
(−)‐**1**	0.33	0.69	0.42	≥10	0.90
(−)**‐9**	0.11	0.09	0.01	0.58	0.11
(+)**‐9**	1.85	0.96	0.24	≥10	0.68
**(*R*)** *‐* **PMPA**	0.13	1.65	0.10	6.10	0.10

[a] EC_50_ Effective concentration of drug required to reduce viral replication by 50 %. Data are the mean from three experiments.

## Conclusion

In summary, D‐ and L‐like enantiomers of 3’‐fluoro‐5’‐norcarbocyclic nucleoside phosphonates incorporating adenine as a nucleobase were synthesized using an efficient optical resolution from a corresponding racemic intermediate, affording the suitable diasteroisomers at the gram scale. The synthesis of the bis(POM) prodrug of each enantiomer was also achieved. All compounds were evaluated against HIV‐1 LAI strain. It appeared from these evaluations that compounds possessing an L‐type configuration, both the phosphonates and their prodrugs, exhibited the best anti‐HIV activities, as compared to (*R)‐*PMPA (Tenofovir). In addition, when the anti‐HIV activity was evaluated against a panel of drug‐resistant viral strains, the antiviral activity of compound (−)‐**1** and its prodrug (−)**‐9**, was also maintained against certain resistant viral strains.

## Experimental Section

^1^H NMR, ^13^C NMR and ^31^P spectra were recorded with proton decoupling at ambient temperature on the following spectrometers: DRX 400 (Bruker BioSpin Corporation, Billerica, MA, USA) or AM‐300 spectrometers (Bruker, BioSpin Corporation). Chemical shifts (*δ*) are quoted in parts per million (ppm) referenced to the appropriate residual solvent peak: CDCl_3_ at 7.26 ppm and 77.16 ppm, [d_6_]DMSO at 2.50 ppm and 39.52 ppm, CD_3_OD at 3.41 ppm and 49.00 ppm, and D_2_O at 4.63 ppm relative to TMS. ^19^F chemical shifts are reported using trichlorofluoromethane as external reference. For ^31^P NMR spectra, chemical shifts are reported relative to external H_3_PO_4_. Signals are indicated as s (singlet), d (doublet), t (triplet), q (quartet), m (multiplet) and br (broad). MS and HRMS were recorded in the positive or negative mode on a Micromass Q‐TOF Waters. UV spectra were recorded with an Uvikon 931 (Kontron) spectrophotometer, *λ* are expressed in nm and *ϵ* in L.mol^−1^.cm^−1^. Thin layer chromatography was performed on pre‐coated aluminum sheets of Silica Gel 60 F_254_ (Merck), and visualization of products was accomplished by UV absorbance and ethanolic acidic *p*‐anisaldehyde solution or 5 % ethanolic sulfuric acid solution, followed by heating. Purification by column chromatography was performed with silica gel 60 (230–400 mesh), flash purification system (Isolera Four) using prepacked columns (FlashPure Silica) or with reversed phase silica gel (RP‐18, 25–40 μm). Ion exchange chromatography was performed on DOWEX 50WX8‐200 resins (Na^+^ form) (Aldrich, Milwaukee). All solvents were distilled under argon atmosphere prior to use: THF from a blue solution of sodium‐benzophenone ketyl radical, CH_2_Cl_2_ and DMF from CaH_2_. All air and/or moisture sensitive reactions were carried out in anhydrous conditions under argon atmosphere using oven‐dried glassware and/or carefully dried in line with a flameless heat gun. Commercially available reagents were used as purchased without further purification.

### (1R,4S)‐4‐{[tert‐butyl(diphenyl)silyl]oxy}‐3‐fluoro‐2‐cyclopenten‐1‐yl (2R)‐(acetyloxy)(phenyl)ethanoate 4 a and (1S,4R)‐4‐{[tert‐butyl(diphenyl)silyl]oxy}‐3‐fluoro‐2‐cyclopenten‐1‐yl (2R)‐(acetyloxy)(phenyl)ethanoate 4 b

To a solution of racemic **3** (1.54 g, 4.32 mmol) in dry CH_2_Cl_2_ (15 mL) at room temperature were added DCC (1.35 g, 6.54 mmol) and DMAP (49 mg, 0.40 mmol). (*R)‐O*‐acetylmandelic acid (1.27 g, 6.52 mmol) was added and the mixture was stirred at room temperature for 3 h. The precipitate was filtrated, washed with CH_2_Cl_2_ and the filtrate was concentrated. The diastereomeric mixture was separated by flash chromatography on silica gel (Petroleum Ether/CH_2_Cl_2_ 80 : 20 to 0 : 100) to afford **4 a** (0.91 g, 40 % yield) and **4 b** (1.01 g) as colorless oils. **Physical data for 4 a**: R_*f*_=0.51 (CH_2_Cl_2_). ^1^H NMR (CDCl_3_, 400 MHz): *δ* 7.68–7.65 (m, 4H, ArH), 7.50–7.37 (m, 11H, ArH), 5.91 (s, 1H, H2), 5.36–5.31 (m, 1H, H1′), 5.16 (d, *J=*2.4 Hz, 1H, H2′), 4.51 (dd, *J*=6.8, 3.5 Hz, 1H, H4′), 2.55 (dt, *J*=14.6, 7.4 Hz, 1H, H5′a), 2.21 (s, 3H, CH_3_), 1.85 (br d, *J*=13.4 Hz, 1H, H5′b), 1.09 (s, 9H, *t‐*Bu). ^13^C NMR (CDCl_3_, 100 MHz): *δ* 170.5 (CO), 168.7 (CO), 166.3 (d, *J=*289.7 Hz, C3’), 136.0 (C_Ar_), 136.0 (C_Ar_), 133.7 (Cq), 133.6 (Cq), 133.2 (Cq), 130.1 (C_Ar_), 130.0 (C_Ar_), 129.4 (C_Ar_), 129.0 (C_Ar_), 127.9 (C_Ar_), 127.8 (C_Ar_), 127.7 (C_Ar_), 105.1 (d, *J=*11.7 Hz, C2′), 74.7 (C2), 72.9 (d, *J=*12.4 Hz, C1′), 70.4 (d, *J=*20.5 Hz, C4′), 39.1 (d, *J=*5.2 Hz, C5′), 26.9 (3xCH_3_), 20.9 (CH_3_), 19.3 (Cq). ^19^F NMR (CDCl_3_, 376.5 MHz): *δ* −121.06. HRMS (ESI^+^): calculated for C_31_H_33_O_5_FNaSi [M+Na]^+^5.1979; found: 555.1976. [α]_D_=+34.5 (*c=*0.55, CHCl_3_). **Physical data for 4 b**: R_*f*_=0.47 (CH_2_Cl_2_). ^1^H NMR (CDCl_3_, 300 MHz): *δ* 7.67–7.59 (m, 4H, ArH), 7.50–7.34 (m, 11H, ArH), 5.90 (s, 1H, H2), 5.37–5.32 (m, 2H, H1’ and H2’), 4.48 (ddd, *J=*5.2, 3.4, 1.2 Hz, 1H, H4’), 2.44 (dt, *J=*14.6, 7.5 Hz, 1H, H5’a), 2.20 (s, 3H, CH_3_CO), *H5’b signal in H_2_O peak*, 1.05 (s, 9H, C(CH_3_)_3_). ^13^C NMR (CDCl_3_, 100 MHz): *δ* 170.4 (CO), 168.6 (CO), 166.1, (d, *J*=289.4 Hz, C3’), 135.9 (C_Ar_), 135.8 (C_Ar_), 133.7 (Cq), 133.6 (Cq), 133.1 (Cq), 130.0 (C_Ar_), 129.9. (C_Ar_), 129.4 (C_Ar_), 128.9 (C_Ar_), 127.8 (C_Ar_), 127.7 (C_Ar_), 127.7 (C_Ar_), 105.2 (d, *J*=12.1 Hz, C2’), 74.7 (C2), 72.8 (d, *J*=12.4 Hz, C4’), 70.3 (d, *J*=20.6 Hz, C1’), 39.0 (d, *J*=5.0 Hz, C5’), 26.8 (3xCH_3_), 20.8 (CH_3_), 19.2 (Cq). ^19^F NMR (CDCl_3_, 376.5 MHz): *δ* −120.99. HRMS (ESI^+^): calculated for C_31_H_33_O_5_FNaSi [M+Na]^+^5.1979; found: 555.1977. [α]_D_=‐102.3 (*c=*0.55, CHCl_3_).

### (1R,4S)‐4‐(tert‐butyldiphenylsilyloxy)‐3‐fluorocyclopent‐2‐enol (+)‐3

To a solution of **4 a** (2.48 g, 4.65 mmol) in THF (50 mL) and H_2_O (50 mL) was added LiOH (0.97 mg, 23 mmol). The mixture was stirred at room temperature for 4 h, then aqueous saturated NH_4_Cl solution (50 mL) was added. The aqueous layer was extracted with CH_2_Cl_2_ (4×50 mL), the combined organic layers were washed with brine, dried (MgSO_4_), filtrated and concentrated to give enantiopure **(+)‐3** as a colorless oil (1.64 g, 99 %) which was used without further purification. R_*f*_=0.29 (PE/EtOAc 8 : 2). ^1^H NMR (CDCl_3_, 300 MHz): *δ* 7.72–7.67 (m, 4H, ArH), 7.47–7.36 (m, 6H, ArH), 5.32 (d, *J=*2.4 Hz, 1H, H2), 4.54–4.50 (m, 1H, H4), 4.47–4.38 (m, 1H, H1), 2.51 (dt, *J*=14.3, 7.3 Hz, 1H, H5a), 1.72–1.64 (m, 1H, H5b), 1.09 (s, 9H, *t‐*Bu). ^13^C NMR (CDCl_3,_ 75 MHz): *δ* 164.8 (d, *J*=288.7 Hz, C3), 136.0 (C_Ar_), 135.9 (2×C_Ar_), 133.7 (Cq), 133.2 (Cq), 130.0 (C_Ar_), 129.9 (C_Ar_), 127.8 (2×C_Ar_), 127.7 (2×C_Ar_), 109.0 (d, *J*=7.2 Hz, C2), 70.9 (d, 7H, *J*=20.8 Hz, C4), 69.1 (d, *J*=10.9 Hz, C1), 43.3 (d, *J*=5.0 Hz, C5), 26.9 (3×CH_3_), 19.2 (Cq). ^19^F (CDCl_3_, 282 MHz): *δ* −124.76 (dt, *J*=6.6, 1.9 Hz). HRMS (ESI^−^): calculated for C_21_H_24_FO_2_Si [M−H]^−^ 355.1530; found: 355.1549. [α]_D_=+28.3 (*c=*1.10, CHCl_3_).

### (1S,4R)‐4‐(tert‐butyldiphenylsilyloxy)‐3‐fluorocyclopent‐2‐enol (−)‐3

Compound **(−)‐3** (1.71 g, 97 %) was obtained from compound **4 b** (2.66 g, 5.02 mmol) according to procedure described for compound **(+)‐3**. [α]_D_ **=**‐27.3 (*c=*1.10, CHCl_3_).

### 9‐((1S,4S)‐4‐{[tert‐butyl(diphenyl)silyl]oxy}‐3‐fluoro‐2‐cyclopenten‐1‐yl)‐6‐chloro‐9H‐purine (−)‐5

To a solution of PPh_3_ (4.24 g, 16.16 mmol) and 6‐chloropurine (2.50 g, 16.16 mmol) in dry THF (110 mL) at 0 °C was added dropwise DIAD (3.2 mL, 16.16 mmol). The mixture was stirred at room temperature for 1 h and then canulated on a solution of **(+)‐4** (1.80 g, 5.05 mmol) in dry THF (30 mL). The reaction mixture was stirred at room temperature for 1 h. The precipitate was removed by filtration, washed with Et_2_O (20 mL) and the filtrate was concentrated. Purification by flash chromatography on silica gel (CH_2_Cl_2_/EtOAc 95 : 5) afforded **(−)‐5** as a white foam (1.34 g, 54 %). R_*f*_=0.30 (CH_2_Cl_2_/EtOAc 95 : 5). ^1^H NMR (CDCl_3_, 400 MHz): *δ* 8.67 (s, 1H, H2), 7.95 (s, 1H, H8), 7.72–7.65 (m, 4H, ArH), 7.47–7.34 (m, 6H, ArH), 5.83–5.74 (m, 1H, H1’), 5.40 (d, 1H, *J=*2.3 Hz, H2’), 5.16–5.08 (m, 1H, H4’), 2.60 (ddd, 1H, *J=*14.4, 8.3, 3.8 Hz, H5’a), 2.16 (ddd, 1H, *J=*14.4, 7.2, 2.6 Hz, H5’b), 1.11 (s, 9H, *t‐*Bu). ^13^C NMR (CDCl_3_, 100 MHz): *δ* 167.3 (d, *J=*291.8, C3’), 152.0 (C2), 151.4 (Cq), 151.3 (Cq), 143.1 (C8), 135.9 (C_Ar_), 135.9 (C_Ar_), 133.3 (Cq), 132.8 (Cq), 132.2 (Cq), 130.2 (C_Ar_), 130.2 (C_Ar_), 128.0 (C_Ar_), 127.9 (C_Ar_), 104.1 (d, *J=*13.3 Hz, C2′), 72.0 (d, *J=*20.3 Hz, C4′), 54.5 (d, *J=*12.1 Hz, C1′), 40.7 (d, *J=*5.1 Hz, C5′), 26.9 (3×CH_3_), 19.3 (Cq). ^19^F NMR (CDCl_3_, 376.5 MHz): *δ* −118.13. UV (EtOH 95) λ_max_=265 nm (ϵ_max=_8000). HRMS (ESI^+^): calculated for C_26_H_27_ClFN_4_OSi [M+H]^+^493.1627; found: 493,1618. [α]_D_=‐38.6 (*c=*0.47, CHCl_3_).

### 9‐((1R,4R)‐4‐{[tert‐butyl(diphenyl)silyl]oxy}‐3‐fluoro‐2‐cyclopenten‐1‐yl)‐6‐chloro‐9H‐purine (+)‐5

Compound **(+)‐5** (1.5 g, 52 %) was obtained from compound **(−)‐4** (2.06 g, 5.80 mmol) according to procedure described for compound **(−)‐5**. [α]_**D**_ **=**+39.2 (*c=*0.48, CHCl_3_).

### N′‐9‐[(1S,4S)‐3‐fluoro‐4‐hydroxy‐2‐cyclopenten‐1‐yl]‐9H‐purin‐6‐yl‐N,N‐dimethylimidoformamide (−)‐6

A solution of **(−)‐5** (657 mg, 1.2 mmol) in methanolic 2 N ammonia (48 mL) was heated at 70 °C in a sealed bomb for 24 h. Solvent was evaporated to dryness and purification by flash chromatography on silica gel (CH_2_Cl_2_/MeOH 97.5 : 2.5 to 90 : 10) afforded the 6‐amino *O*‐deprotected product contaminated with by‐products (221 mg). The mixture was used for the next step without further purification. To a solution of the previously prepared compound in DMF (5 mL) was added DMF‐dimethylacetal (370 μL, 2.79 mmol). The reaction mixture was heated at 50 °C for 15 h, then cooled down to room temperature and concentrated to dryness. Purification by flash chromatography on silica gel (CH_2_Cl_2_ to CH_2_Cl_2_/MeOH 90 : 10) gave **(−)‐6** (241 mg, 68 %) as a colorless oil. Rf=0.26 (CH_2_Cl_2_/MeOH 90 : 10). ^1^H NMR (CDCl_3_, 400 MHz): *δ* 8.92 (s, 1H, H2), 8.52 (s, 1H, H8), 7.84 (s, 1H, NCH=N), 5.77–5.71 (m, 1H, H1’), 5.41 (d, 1H, *J=*2.5 Hz, H2’), 5.17 (br s, 1H, H4’), 3.46 (s., 1H, OH), 3.24 (s, 3H, CH_3_), 3.18 (s, 3H, CH_3_), 2.61 (ddd, 1H, *J=*13.8, 8.1, 3.8 Hz, H5’a), 2.45 (ddd, 1H, *J=*14.6, 7.5, 2.9 Hz, H5’b).;^13^C NMR (CDCl_3_, 100 MHz): *δ* 166.8 (d, *J=*289.2 Hz, C3’), 159.7 (Cq), 158.2 (C2), 152.6 (C8), 151.4 (Cq), 139.4 (CH=N), 126.4 (Cq), 104.8 (d, *J=*12.1 Hz, C2’), 70.3 (d, *J=*2.0 Hz, C4’), 53.6 (d, *J=*12.1 Hz, C1’), 41.3 (CH_3_), 40.2, (d, *J=*5.2 Hz, C5’), 35.2 (CH_3_). ^19^F NMR (CDCl_3_, 376.5 MHz): *δ* −121.97. UV (EtOH 95) λ_max_=310 nm (ϵ_max=_15000). HRMS (ESI^+^): calculated for C_13_H_16_FN_6_O [M+H]^+^291.1370; found 291.1367. [α]_D_=‐172.0 (*c=*0.5, CHCl_3_).

### N′‐9‐[(1R,4R)‐3‐fluoro‐4‐hydroxy‐2‐cyclopenten‐1‐yl]‐9H‐purin‐6‐yl‐N,N‐dimethylimidoformamide (+)‐6

Compound **(+)‐6** (266 mg, 71 %) was obtained from compound **(+)‐5** (705 mg, 1.29 mmol) according to procedure described for compound **(−)‐6**. [α]_D_ **=**+176.0 (*c=*0.5, CHCl_3_).

### N′‐9‐[(1S,4R)‐3‐fluoro‐4‐hydroxy‐2‐cyclopenten‐1‐yl]‐9H‐purin‐6‐yl‐N,N‐dimethylimidoformamide (−)‐7

A mixture of PPh_3_ (2.49 g, 9.5 mmol) and benzoic acid (1.16 g, 9.5 mmol) in dry THF (10 mL) was canulated on a solution of alcohol **(−)‐6** (553 mg, 1.9 mmol) in dry THF (10 mL). The reaction mixture was cooled to 0 °C and DIAD (1.87 mL, 9.5 mmol) was added. The resulting solution was allowed to reach room temperature, stirred for 3 h and then concentrated to dryness. The residue was dissolved in MeOH (38 mL) and K_2_CO_3_ (524 mg, 3.8 mmol) was added. The reaction mixture was stirred at room temperature for 2 h then concentrated to dryness. Purification by flash chromatography on silica gel (CH_2_Cl_2_ to CH_2_Cl_2_/MeOH 90 : 10) afforded **(−)‐7** (472 mg, 85 %) as a colorless oil. R_*f*_=0.45 (CH_2_Cl_2_/MeOH 9 : 1). ^1^H NMR (CDCl_3_, 400 MHz):*δ* 8.95 (s, 1H, H2), 8.48 (s, 1H, H8), 7.88 (s, 1H, CH=N), 7.37 (d, 1H, *J=*11.0 Hz, OH), 5.28–5.21 (m, 1H, H1′), 5.21 (s, 1H, H2′), 4.62 (d, *J=*7.4 Hz, 1H, H4′), 3.27 (s, 3H, CH_3_), 3.22 (s, 3H, CH_3_), 3.13–3.05 (m, 1H, H5′a), 2.35 (dd, *J*=15.6, 2.3 Hz, 1H, H5’b). ^13^C NMR (CDCl_3_, 100 MHz): *δ* 167.6 (d, *J=*290.2 Hz, C3’), 160.2 (Cq), 158.4 (C2), 151.6 (C8), 150.3 (Cq), 141.7 (CH=N), 127.7 (Cq), 104.2 (d, *J=*12.2 Hz, C2′), 70.4 (d, *J=*22.2 Hz, C4′), 55.3 (d, *J=*11.5 Hz, C1′), 41.5 (CH_3_), 37.9 (d, *J=*5.7 Hz, C5′), 35.4 (CH_3_). ^19^F NMR (CDCl_3_, 376.5 MHz): *δ* −123.25. UV (EtOH 95) λ_max_=312 nm (ϵ_max=_23415). HRMS (ESI^+^): calculated for C_13_H_16_N_6_OF [M+H]^+^291.1370; found: 291.1369. [α]_D_=‐236.4 (*c=*0.55, CHCl_3_).

### N′‐9‐[(1R,4S)‐3‐fluoro‐4‐hydroxy‐2‐cyclopenten‐1‐yl]‐9H‐purin‐6‐yl‐N,N‐dimethylimidoformamide (+)‐7

Compound **(+)‐7** (489 mg, 85 %) was obtained from compound **(+)‐6** (572 mg, 1.97 mmol) according to procedure described for compound **(−)‐7**. [α]_D_ **=**+231.5 (*c=*0.54, CHCl_3_).

### Diethyl‐[(1R,4S)‐4‐(6‐amino‐9H‐purin‐9‐yl)‐2‐fluoro‐2‐cyclopenten‐1‐yl]oxymethylphosphonate (−)‐8

To a solution of **(−)‐7** (198 mg, 0.68 mmol) in dry THF (10 mL) at 0 °C was added LiO*t*Bu (2.2 M solution in THF, 0.93 mL, 2.05 mmol). The solution was stirred at 0 °C for 1 h until addition of diethyl(tosyloxymethyl)phosphonate (879 mg, 2.73 mmol). The reaction mixture was stirred at room temperature for 3 days, and then quenched by addition of AcOH (few drops). Solvents were evaporated, the residue was dissolved in AcOH/MeOH/H_2_O (9 : 1 : 1, 10 mL) and stirred at room temperature for 24 h. After concentration and purification by flash chromatography on silica gel (CH_2_Cl_2_/MeOH 98 : 2 to 90 : 10), compound **(−)‐8** (187 mg, 71 %) was obtained as a yellowish foam. R_*f*_=0.46 (CH_2_Cl_2_/MeOH 9 : 1). ^1^H NMR (300 MHz, MeOD): *δ* 8.21 (s, 1H, H2), 8.16 (s, 1H, H8), 5.69 (d, *J*=2.5 Hz, 1H, H3’), 5.55 (m, 1H, H4’), 4.87 (s, 2H, NH_2_), 4.65 (dd, *J*=7.0, 1.8 Hz, 1H, H1’), 4.21–4.01 (m, 6H, 2xCH_2_), 3.06 (dt, *J*=15.2, 7.7 Hz, 1H, H5’a), 2.09 (dd, *J*=15.0, 2.2 Hz, 1H, H5’b), 1.34–1.28 (m, 6H, 2xCH_3_). ^13^C NMR (75 MHz, MeOD): *δ* 166.2 (d, *J*=287.2 Hz, C2’), 157.3 (Cq), 153.8 (C2), 150.2 (Cq), 140.7 (C8), 120.2 (Cq), 108.5 (d, *J*=12.5 Hz, C3’), 80.7 (dd, *J*=21.0, 13.7 Hz, C1’), 64.3 (d, *J*=167.8 Hz, OCH_2_P), 64.2 (d, *J*=6.6 Hz, 2xCH_2_CH_3_), 53.3 (d, *J*=11.2 Hz, C4’), 37.8 (d, *J*=5.3 Hz, C5’), 16.74 (d, *J*=5.7 Hz, 2xCH_3_). ^31^P NMR (121 MHz, MeOD): *δ* 21.41. ^19^F NMR (282 MHz, MeOD): *δ* −123.24 (d, *J*=2.5 Hz). UV (EtOH 95) λ_max_=262 nm (ϵ_max=_13500). HRMS (ESI^+^): calculated for C_15_H_22_FN_5_O_4_P [M+H]^+^386.1393; found 386.1405. [α]_D_=‐31.7 (*c=*0.60, CHCl_3_).

### Diethyl‐[(1S,4R)‐4‐(6‐amino‐9H‐purin‐9‐yl)‐2‐fluoro‐2‐cyclopenten‐1‐yl]oxymethylphosphonate (+)‐8

Compound **(+)‐8** (173 mg, 68 %) was obtained from compound **(+)‐7** (192 mg, 0.66 mmol) according to procedure described for compound **(−)‐8**. [α]_D_ **=**+32.4 (*c=*0.62, CHCl_3_).

### Sodium‐{[(1R,4S)‐4‐(6‐amino‐9H‐purin‐9‐yl)‐2‐fluoro‐2‐cyclopenten‐1‐yl]oxymethylphosphonic acid (−)‐1

To a solution of **(−)‐8** (79 mg, 0.205 mmol) in dry DMF (5 mL) at 0 °C was added TMSBr (0.4 mL, 3.08 mmol). The reaction mixture was stirred at room temperature for 16 h before addition of TEAB (1 M) until pH=7. Solvents were evaporated to dryness and purification by flash chromatography on RP‐18 (H_2_O to H_2_O/MeOH 70/30) followed by Dowex (Na+) percolation afforded compound **(−)‐1** (32 mg, 42 %) as a white lyophilisate. R_*f*_ **=**0.19 (*i*PrOH/NH_4_OH/H_2_O: 7/2/1). ^1^H NMR (400 MHz, D_2_O): *δ* 8.13 (s, 1H, H2), 8.01 (s, 1H, H8), 5.62 (d, *J*=2.5 Hz, 1H, H3’), 5.35–5.29 (m, 1H, H4’), 4.70 (dd, *J*=7.4, 2.7 Hz, 1H, H1’), 3.71 (d, *J=*9.3 Hz, 2H, OCH_2_P), 3.08 (dt, *J*=15.5, 7.9 Hz, 1H, H5’a), 1.98 (br d, *J*=14.9 Hz, 1H, H5’b). ^13^C NMR (101 MHz, D_2_O): *δ* 164.6 (d, *J*=286.2 Hz, C2’), 155.1 (Cq), 152.01 (C2), 148.1 (Cq), 140.6 (C8), 118.4 (Cq), 106.8 (d, *J=*13.3 Hz, C3’), 78.7 (dd, *J*=20.3, 12.7 Hz, C1’), 65.8 (d, *J*=156.2 Hz, OCH_2_P), 52.07 (d, *J*=11.4 Hz, C4’), 36.1 (C5’). ^31^P NMR (162 MHz, D_2_O): *δ* 15.03. ^19^F NMR (D_2_O, 376.5 MHz): *δ* −121.64. UV (H_2_O) λ_max_=261 nm (ϵ_max=_13300). HRMS (ESI^+^): calculated for C_11_H_14_FN_5_O_4_P [M+H]^+^30.0767 found 330.0743. [α]_D_=‐18.9 (c=0.53, H_2_O).

### Sodium‐{[(1S,4R)‐4‐(6‐amino‐9H‐purin‐9‐yl)‐2‐fluoro‐2‐cyclopenten‐1‐yl]oxymethylphosphonic acid (+)‐1

Compound **(+)‐1** (36 mg, 44 %) was obtained from compound **(+)‐8** (84 mg, 0.217 mmol) according to procedure described from compound **(−)‐1**. [α]_D_ **=**+20.2 (c=0.55, H_2_O).

### bis‐(Pivaloyloxymethyl)‐((1’R,4’S)‐4‐(6‐amino‐9H‐purin‐9‐yl)‐2‐fluorocyclopent‐2‐en‐1‐yl)oxymethyl‐phosphonate (−)‐9

NEt_3_ (72 μL, 0.52 mmol) was added to a solution of **(−)‐1** (3xBu_4_N^+^ salt, 181 mg, 0.164 mmol) in dry DMF (1 mL). The solution was stirred at room temperature for 30 minutes until the addition of chloromethyl pivalate (122 μL, 0.839 mmol). The reaction mixture was stirred at room temperature for 5 days and then quenched by the addition of water (5 mL) and CH_2_Cl_2_ (5 mL). The layers were separated and the aqueous layer was extracted with CH_2_Cl_2_ (3x5 mL). The combined organic layers were dried (MgSO_4_), filtrated and concentrated. Purification by flash chromatography on silica gel (CH_2_Cl_2_ to CH_2_Cl_2_/MeOH 96/4) afforded the bis‐POM‐prodrug (6.8 mg, 28 %) as a colorless oil. R_*f*_=0.29 (CH_2_Cl_2_/MeOH 95/5). ^1^H NMR (400 MHz, CDCl_3_): *δ* 8.34 (s, 1H, H2), 8.02 (s, 1H, H8), 5.87 (br.s, 2H, NH_2_), 5.74 – 5.61 (m, 4H, 2xOCH_2_O), 5.57–5.51 (m, 1H, H4’), 5.50 (d, *J*=2.7 Hz, 1H, H3’), 4.60 (dd, *J*=7.5, 2.3 Hz, 1H, H1’), 4.11 (dd, *J*=14.0, 8.4 Hz, 1H, CHaHbP), 3.99 (dd, *J*=14.0, 8.6 Hz, 1H, CHaHbP), 3.06 (ddd, *J*=15.4, 7.8 Hz, 1H, H5’a), 2.06 (ddd, *J*=15.1, 4.9, 2.5 Hz, 1H, H5’b), 1.22 (s, 9H, C(CH_3_)_3_), 1.20 (s, 9H, C(CH_3_)_3_) ^13^C NMR (101 MHz, CDCl_3_): *δ* 176.8 (C=O), 164.6 (d, *J*=289.5 Hz, C2’), 155.4 (Cq), 152.8 (C2), 149.5 (Cq), 139.0 (C8), 119.6 (Cq), 107.5 (d, *J*=11.6 Hz, C3’), 82.8 (OCH_2_O), 79.3 (dd, *J*=20.5, 10.8 Hz, C1’), 64.4 (d, *J*=167.6 Hz, CH_2_P), 51.1 (d, *J*=10.6 Hz, C4’), 38.7 (Cq), 37.3 (d, *J*=5.1 Hz, C5’), 26.8 (CH_3_) ^31^P NMR (162 MHz, CDCl_3_): *δ* 20.16. ^19^F NMR (376.5 MHz, CDCl_3_,): *δ* −119.94. UV (EtOH) λ_max_=260 nm (ϵ_max_=7000). HRMS (ESI^+^): calculated for C_23_H_34_N_5_O_8_FP [M+H]+58.2129; found 558.2132.

### bis‐(Pivaloyloxymethyl)‐((1’S,4’R)‐4‐(6‐amino‐9H‐purin‐9‐yl)‐2‐fluorocyclopent‐2‐en‐1‐yl)oxymethyl‐phosphonate (+)‐9

Following the procedure described for **(−)‐9**, and starting from **(+)‐1** (3xBu_4_N^+^ salt, 175 mg, 0.159 mmol), **(+)‐9** was obtained as a colorless oil (7 mg, 30 %).

### Cells and virus

Peripheral blood mononuclear cells (PBMC) from healthy donors were isolated and stimulated for 3 days with 1 mg/mL of phytohemagglutinin‐P (PHA‐P, Sigma) and 5 IU/mL of recombinant human interleukin‐2 (rHuIL‐2, Roche). PBMC were growth under CO_2_ in a humid atmosphere at 37 °C in RPMI‐1640 GlutaMAX medium supplemented with antibiotics (penicillin, streptomycin, neomycin), 10 % fetal calf serum (FCS, previously inactivated by heat) and 10 UI/mL of rHuIL‐2. The HIV‐1 LAI strain was previously described.^[19]^ Resistant viral strains are from the NIH AIDS Research & Reference Reagent Program (HIV‐1–52534‐2, HIV‐1–56252‐1, HIV‐1–71361‐1, and HIV‐1–7324‐1) carry mutations in RT.^[16]^

### Anti‐HIV assay

PHA‐P activated PBMCs (1.5×10^5^ cells) were pre‐treated for 30 min by increasing concentrations of the various compounds to be tested and then infected with 100 % infectious tissue culture doses 50 % (TCID_50_) of the HIV‐1‐LAI. Supernatants were collected at day 7 post infection and stored at ‐ 20 °C. Viral replication was measured by quantifying reverse transcriptase activity in cell culture supernatants by the use of Lenti kit RT (Cavidi). Cytotoxicity of the compounds was evaluated in uninfected PHA‐P PBMC by MTT assay (Promega) on day 7. Experiments were performed in triplicate and repeated with another blood donors. Data analyses were performed using SoftMax® Pro 4.6 software (Molecular Devices). Percent of inhibition of RT activity or cell viability were plotted versus compound concentration and fitted with quadratic curves to determine 50 % effective concentration (EC_50_) and 50 % cytotoxic concentration (CC_50_). The viral resistant strains were amplified and the viral stocks titrated using cells of the H9 line.^[20]^ More specifically, with regard to the antiviral test, the cells were distributed in the wells of 96‐well microplates at the rate of 50,000 cells per well. They were then pretreated for 30 minutes with increasing concentrations of the various compounds to be tested and then infected with 125 TCID_50_ of the various resistant strains. Seven and 14 days after infection, culture supernatants were collected and frozen at −20 ° C until measurement of viral replication via quantification of the enzymatic activity of the TI. The EC_50_ was calculated as previously described. A possible cytotoxicity of the molecules towards the cells of the H9 line was assessed by microscopic observation throughout the cell cultures.

## Conflict of interest

The authors declare no conflict of interest.

1

## Supporting information

As a service to our authors and readers, this journal provides supporting information supplied by the authors. Such materials are peer reviewed and may be re‐organized for online delivery, but are not copy‐edited or typeset. Technical support issues arising from supporting information (other than missing files) should be addressed to the authors.

Supporting Information

## Data Availability

The data that support the findings of this study are available from the corresponding author upon reasonable request.
