# Supplementary material for: Discovery of an L‐like Configuration for 3’‐Fluoro‐5’‐norcarbonucleoside Phosphonates as Potent Anti‐HIV Agents
Source: ChemMedChem. 2022 Sep 15;17(20):e202200377. doi: 10.1002/cmdc.202200377 (PMC9825896; doi:10.1002/cmdc.202200377)

ChemMedChem

Supporting Information

Discovery of an L-like Configuration for 3′-Fluoro-5′-norcarbonucleoside Phosphonates as Potent Anti-HIV Agents

Pierre-Yves Geant, Malika Kaci, Jean-Pierre Uttaro, Christian Périgaud, and Christophe Mathé*

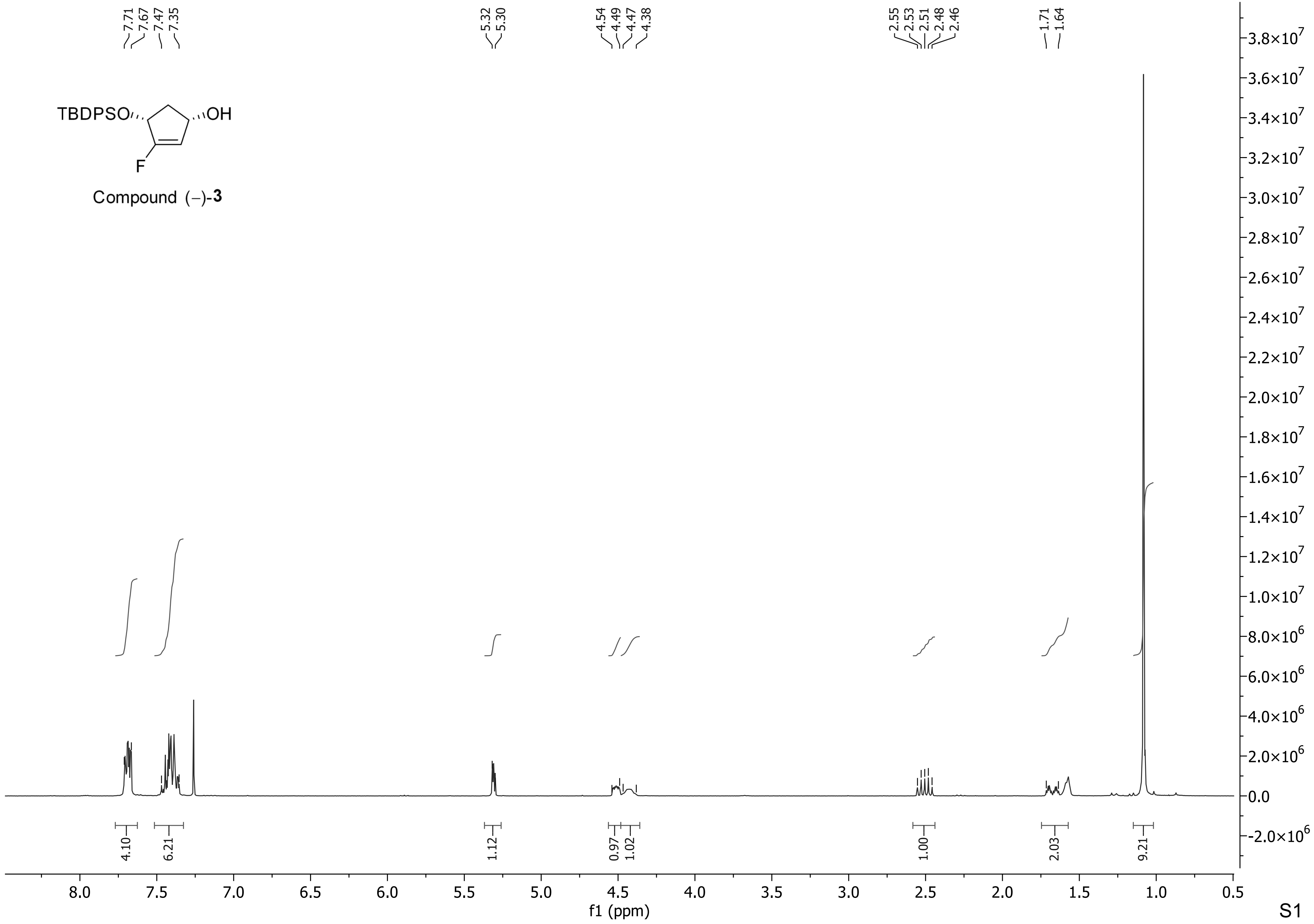

TBDPSO
OH
F
Compound (–)-3
7.71
7.67
7.47
7.35
5.32
5.30
4.54
4.49
4.47
4.38
2.55
2.53
2.51
2.48
2.46
1.71
1.64
4.10
6.21
1.12
0.97
1.02
1.00
2.03
9.21
f1 (ppm)

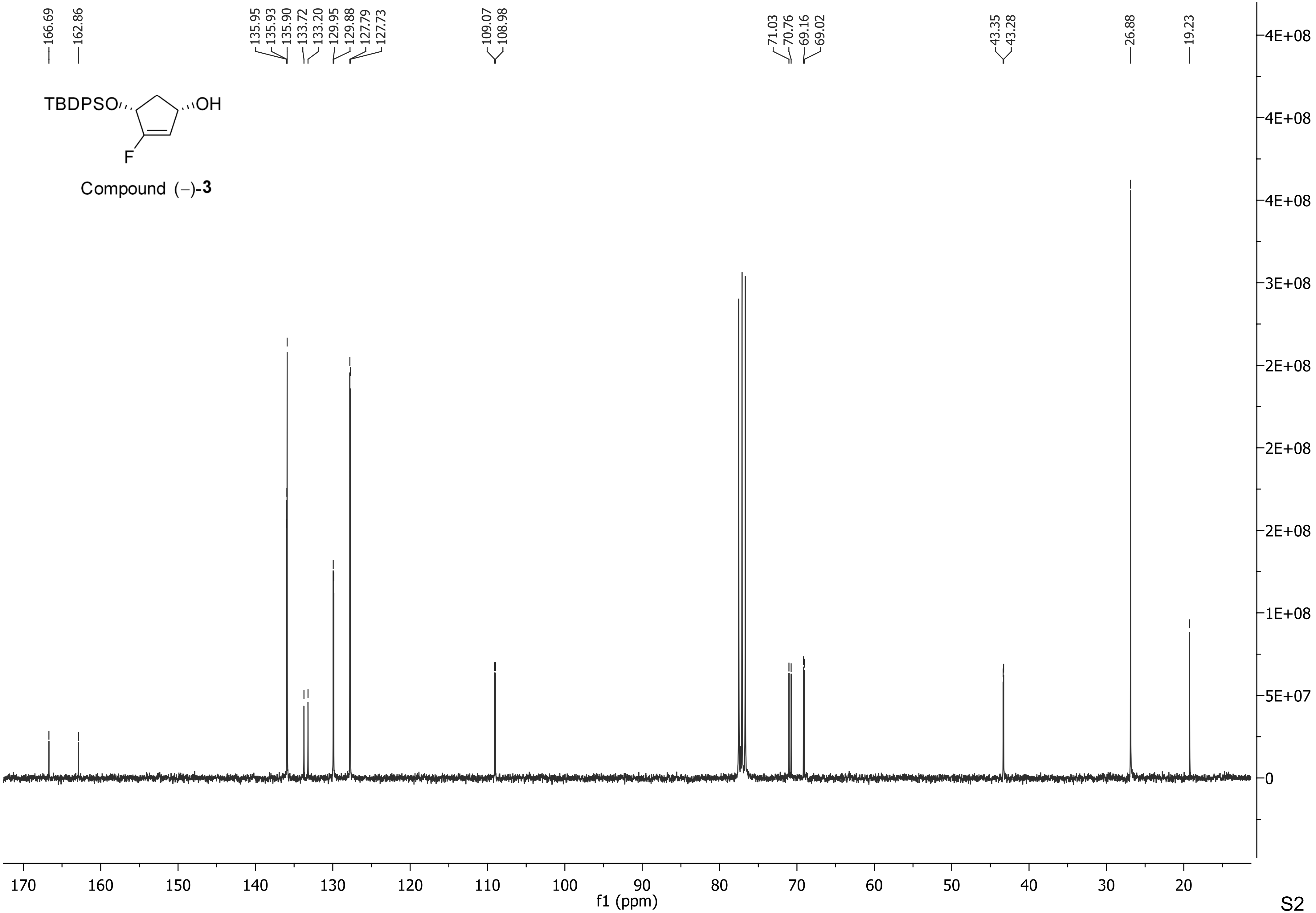
166.69
162.86
135.95
135.93
135.90
133.72
133.20
129.95
129.88
127.79
127.73
109.07
108.98
71.03
70.76
69.16
69.02
43.35
43.28
26.88
19.23
TBDPSO
OH
F
Compound (–)-3
f1 (ppm)

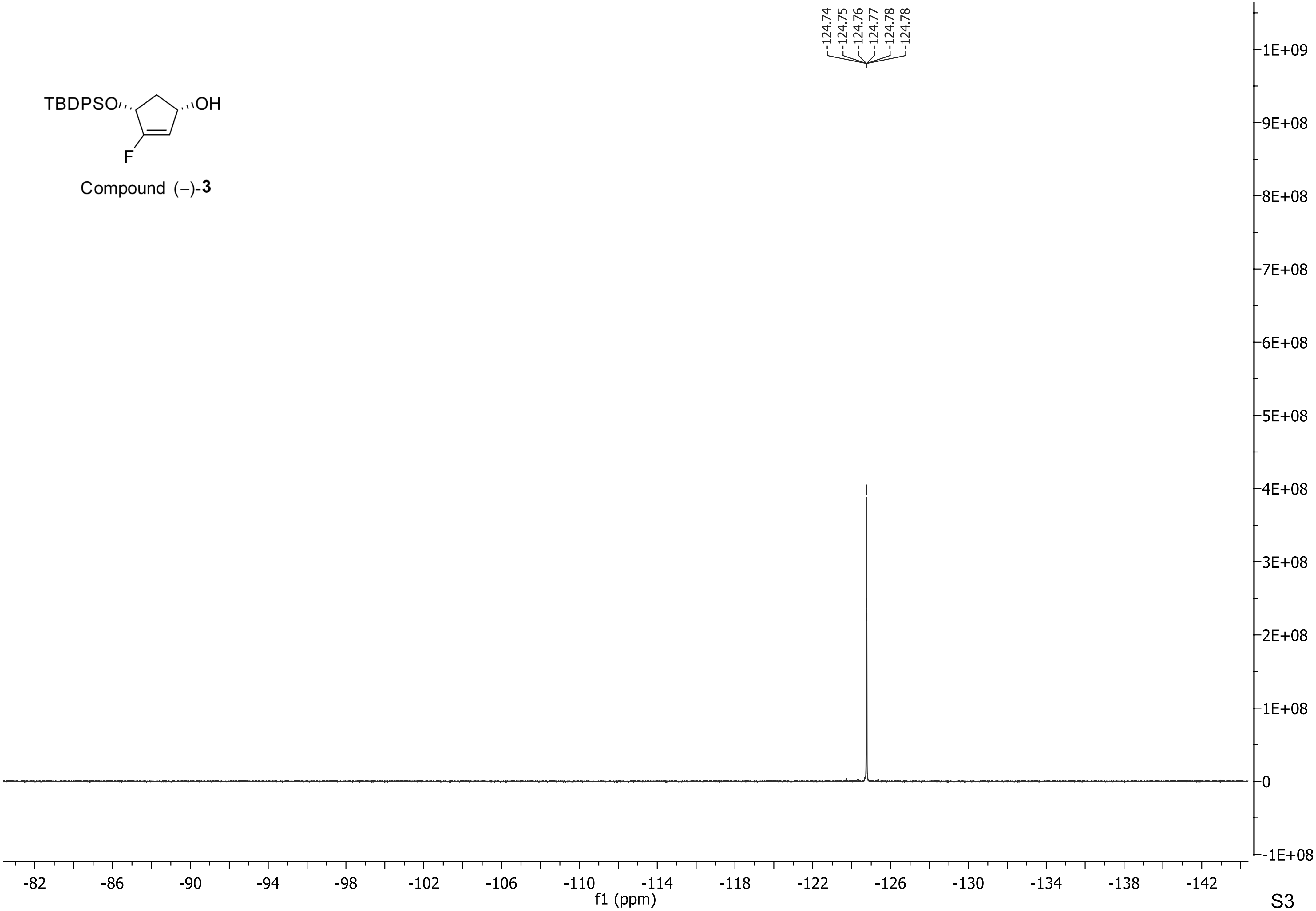
TBDPSO
OH
F
-124.74
-124.75
-124.76
-124.77
-124.78
-124.78
1E+09
9E+08
8E+08
7E+08
6E+08
5E+08
4E+08
3E+08
2E+08
1E+08
0
-1E+08
-82 -86 -90 -94 -98 -102 -106 -110 -114 -118 -122 -126 -130 -134 -138 -142
f1 (ppm)

Compound (–)-**3**

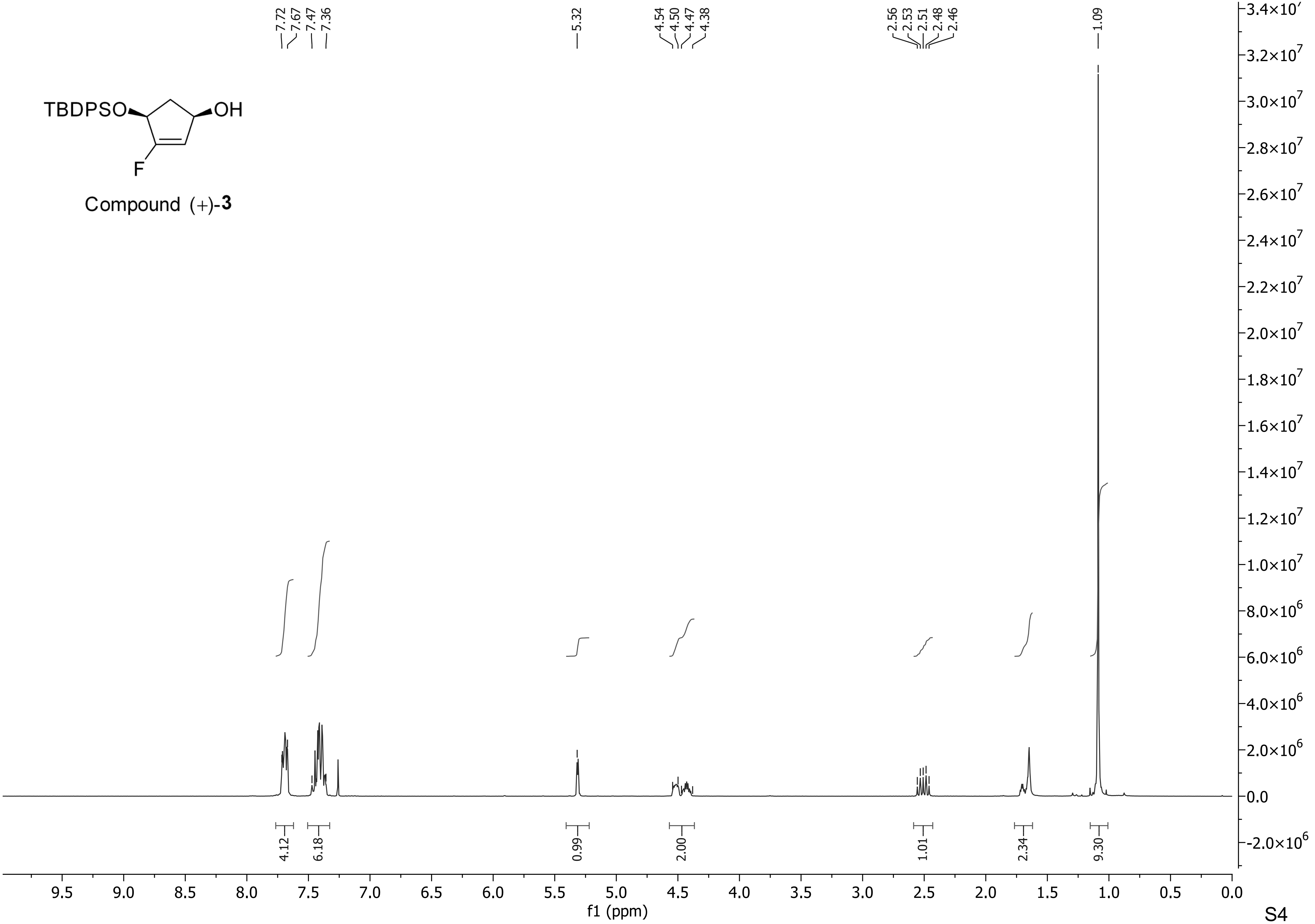
7.72
7.67
7.47
7.36
5.32
4.54
4.50
4.47
4.38
2.56
2.53
2.51
2.48
2.46
1.09
TBDPSO
OH
F
4.12
6.18
0.99
2.00
1.01
2.34
9.30
9.5 9.0 8.5 8.0 7.5 7.0 6.5 6.0 5.5 5.0 4.5 4.0 3.5 3.0 2.5 2.0 1.5 1.0 0.5 0.0
f1 (ppm)

Compound (+)-**3**

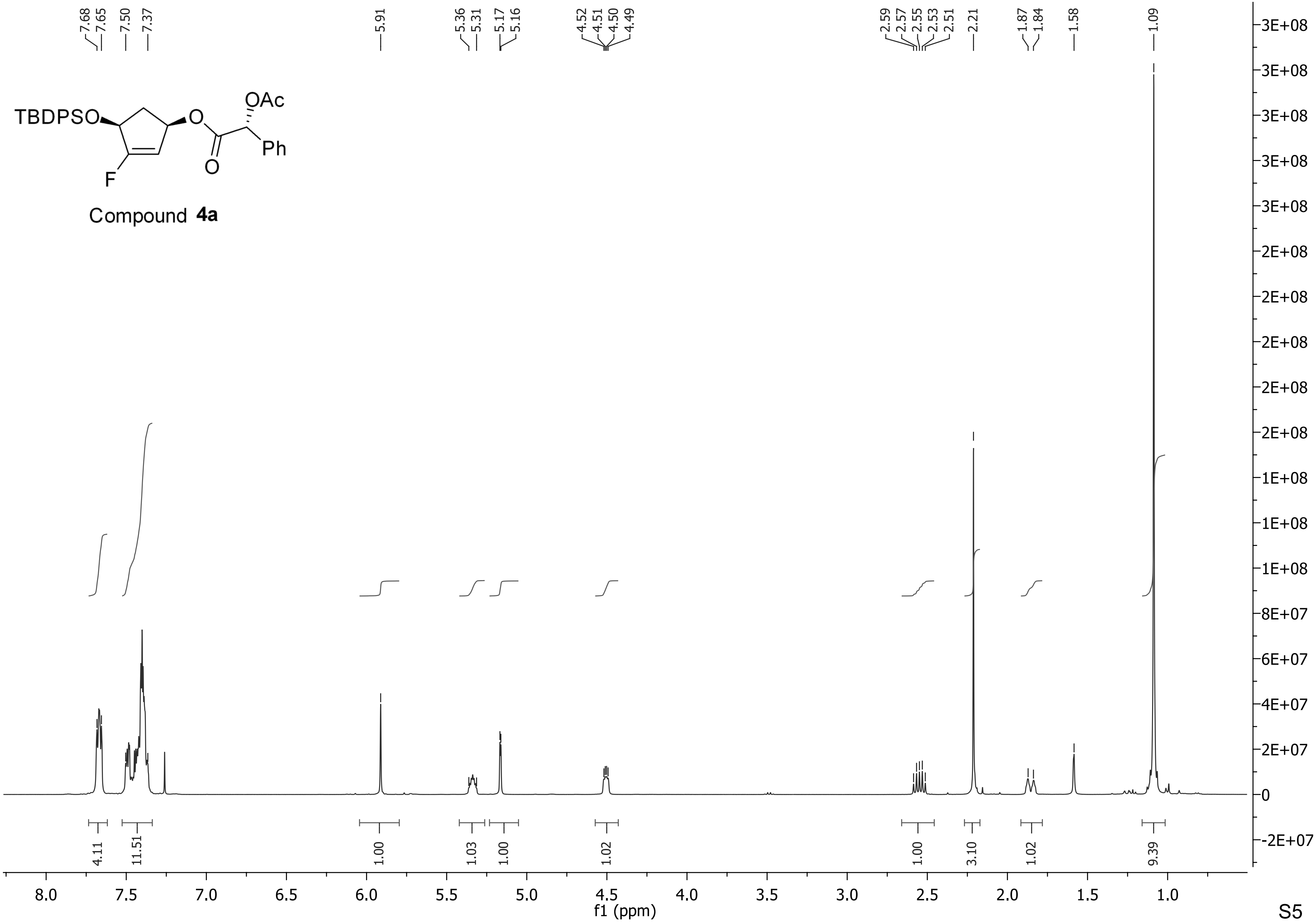
7.68
7.65
7.50
7.37
5.91
5.36
5.31
5.17
5.16
4.52
4.51
4.50
4.49
2.59
2.57
2.55
2.53
2.51
2.21
1.87
1.84
1.58
1.09
TBDPSO
OAc
O
O
Ph
F
Compound 4a
4.11
11.51
1.00
1.03
1.00
1.02
1.00
3.10
1.02
9.39
8.0
7.5
7.0
6.5
6.0
5.5
5.0
4.5
4.0
3.5
3.0
2.5
2.0
1.5
1.0
f1 (ppm)
3E+08
3E+08
3E+08
3E+08
3E+08
2E+08
2E+08
2E+08
2E+08
2E+08
1E+08
1E+08
1E+08
8E+07
6E+07
4E+07
2E+07
0
-2E+07

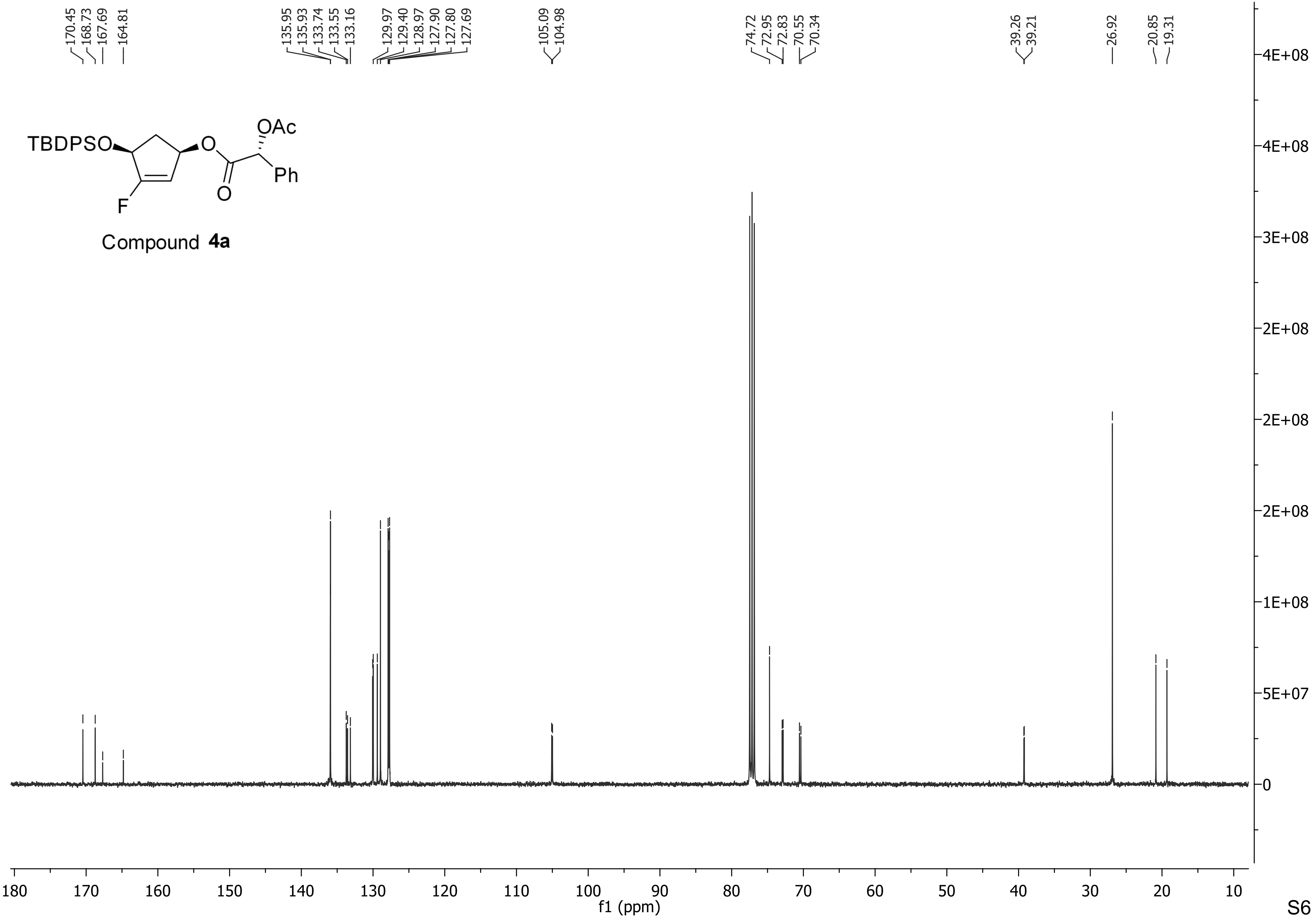

170.45
168.73
167.69
164.81
135.95
135.93
133.74
133.55
133.16
129.97
129.40
128.97
127.90
127.80
127.69
105.09
104.98
74.72
72.95
72.83
70.55
70.34
39.26
39.21
26.92
20.85
19.31
TBDPSO
OAc
O
Ph
O
F
4E+08
4E+08
3E+08
2E+08
2E+08
2E+08
1E+08
5E+07
0
180
170
160
150
140
130
120
110
100
90
80
70
60
50
40
30
20
10
f1 (ppm)

Compound **4a**

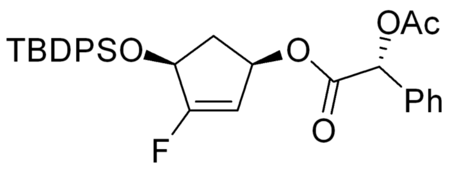
TBDPSO
OAc
O
Ph
F
O

Compound **4a**

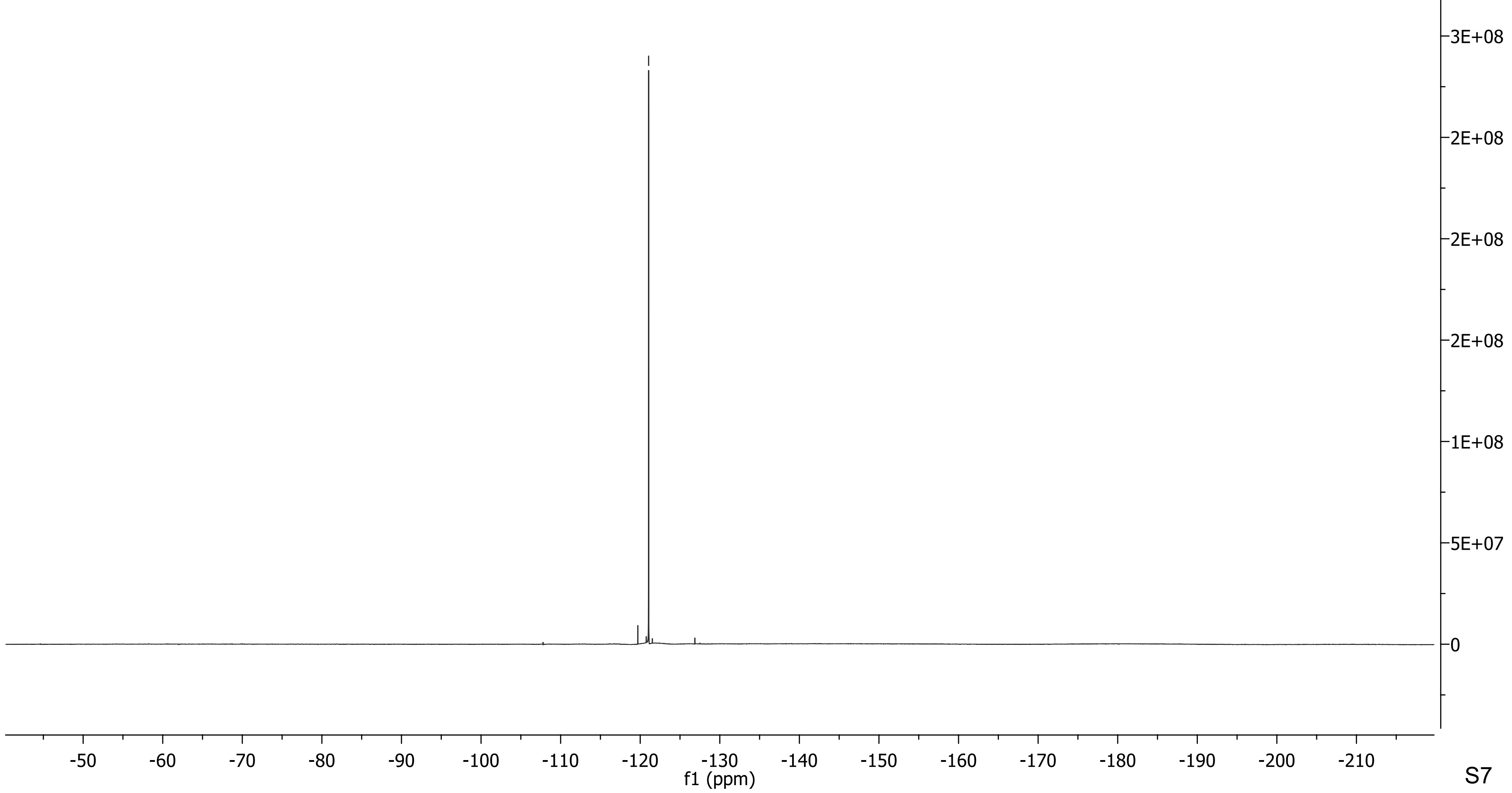
-121.06
4E+08
4E+08
3E+08
2E+08
2E+08
2E+08
1E+08
5E+07
0
-50
-60
-70
-80
-90
-100
-110
-120
-130
-140
-150
-160
-170
-180
-190
-200
-210
f1 (ppm)

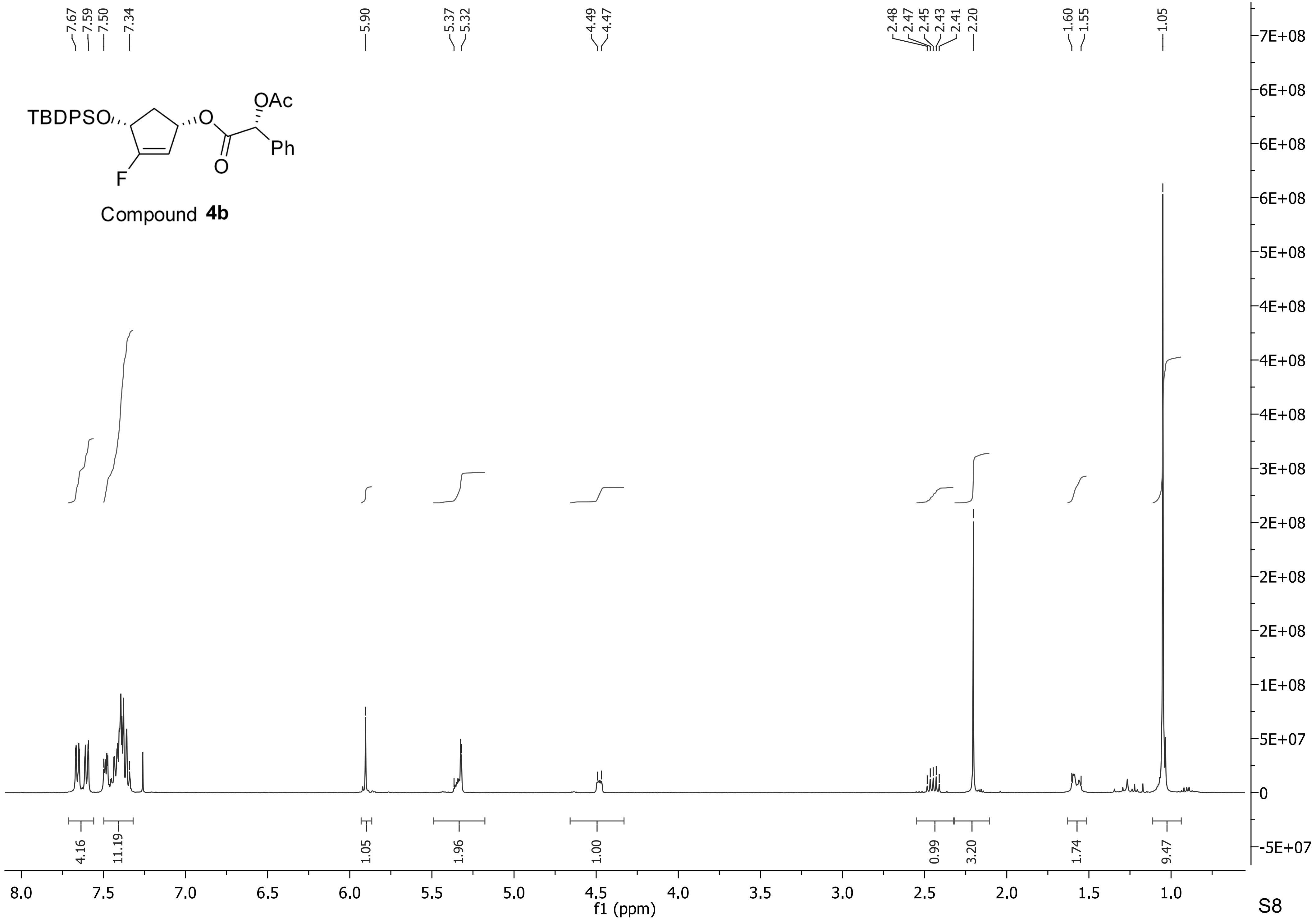
7.67
7.59
7.50
7.34
5.90
5.37
5.32
4.49
4.47
2.48
2.47
2.45
2.43
2.41
2.20
1.60
1.55
1.05
TBDPSO
OAc
O
O
Ph
F
Compound 4b
4.16
11.19
1.05
1.96
1.00
0.99
3.20
1.74
9.47
8.0
7.5
7.0
6.5
6.0
5.5
5.0
4.5
4.0
3.5
3.0
2.5
2.0
1.5
1.0
f1 (ppm)
7E+08
6E+08
6E+08
6E+08
5E+08
4E+08
4E+08
4E+08
3E+08
2E+08
2E+08
2E+08
1E+08
5E+07
0
-5E+07

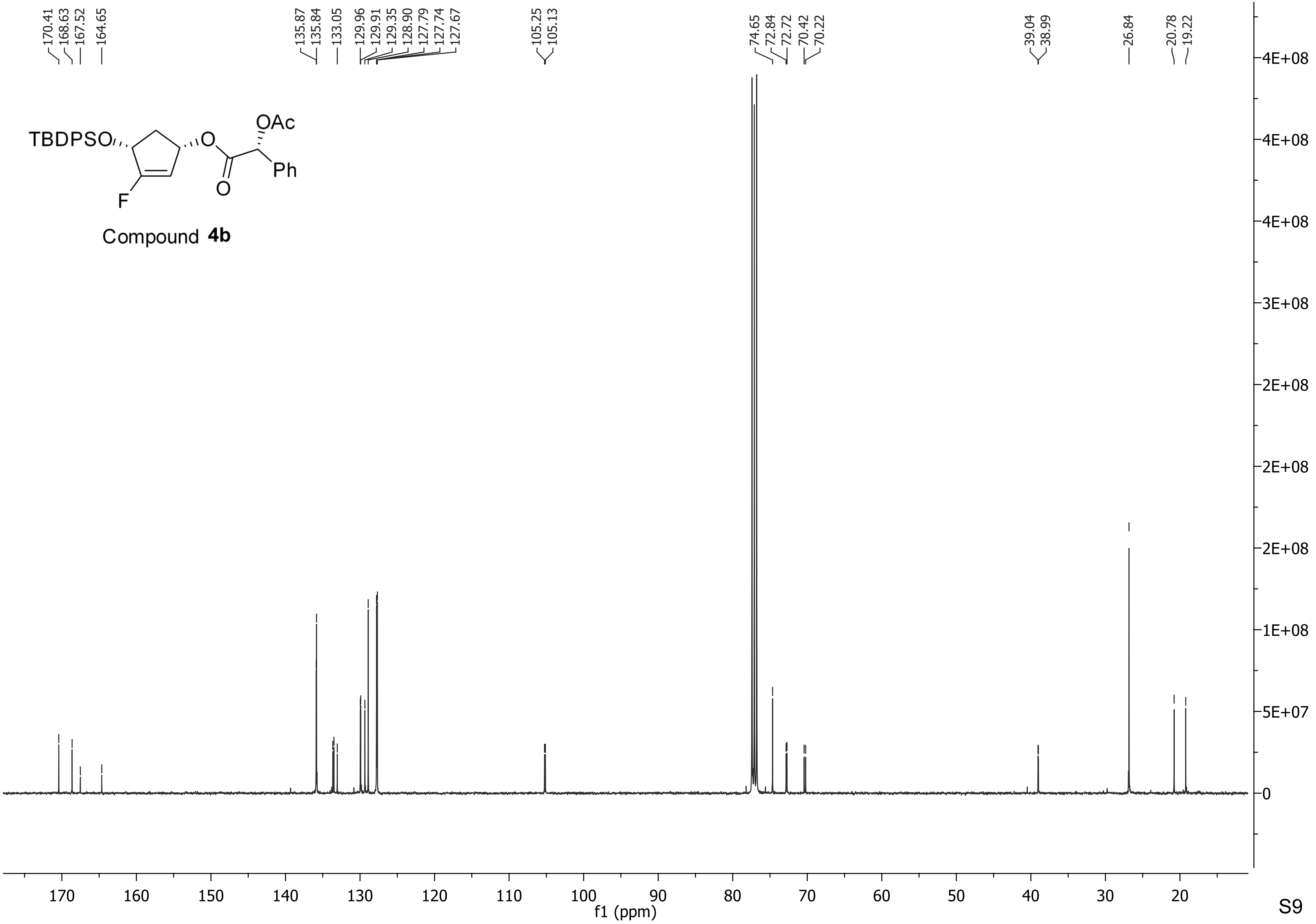
170.41
168.63
167.52
164.65
135.87
135.84
133.05
129.96
129.91
129.35
128.90
127.79
127.74
127.67
105.25
105.13
74.65
72.84
72.72
70.42
70.22
39.04
38.99
26.84
20.78
19.22
TBDPSO
OAc
O
Ph
F
4E+08
3E+08
2E+08
1E+08
5E+07
0
170
160
150
140
130
120
110
100
90
80
70
60
50
40
30
20
f1 (ppm)

Compound **4b**

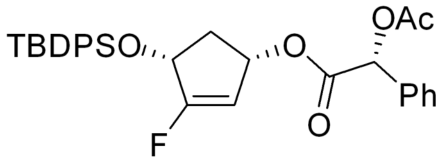
TBDPSO
OAc
O
Ph
O
F

Compound **4b**

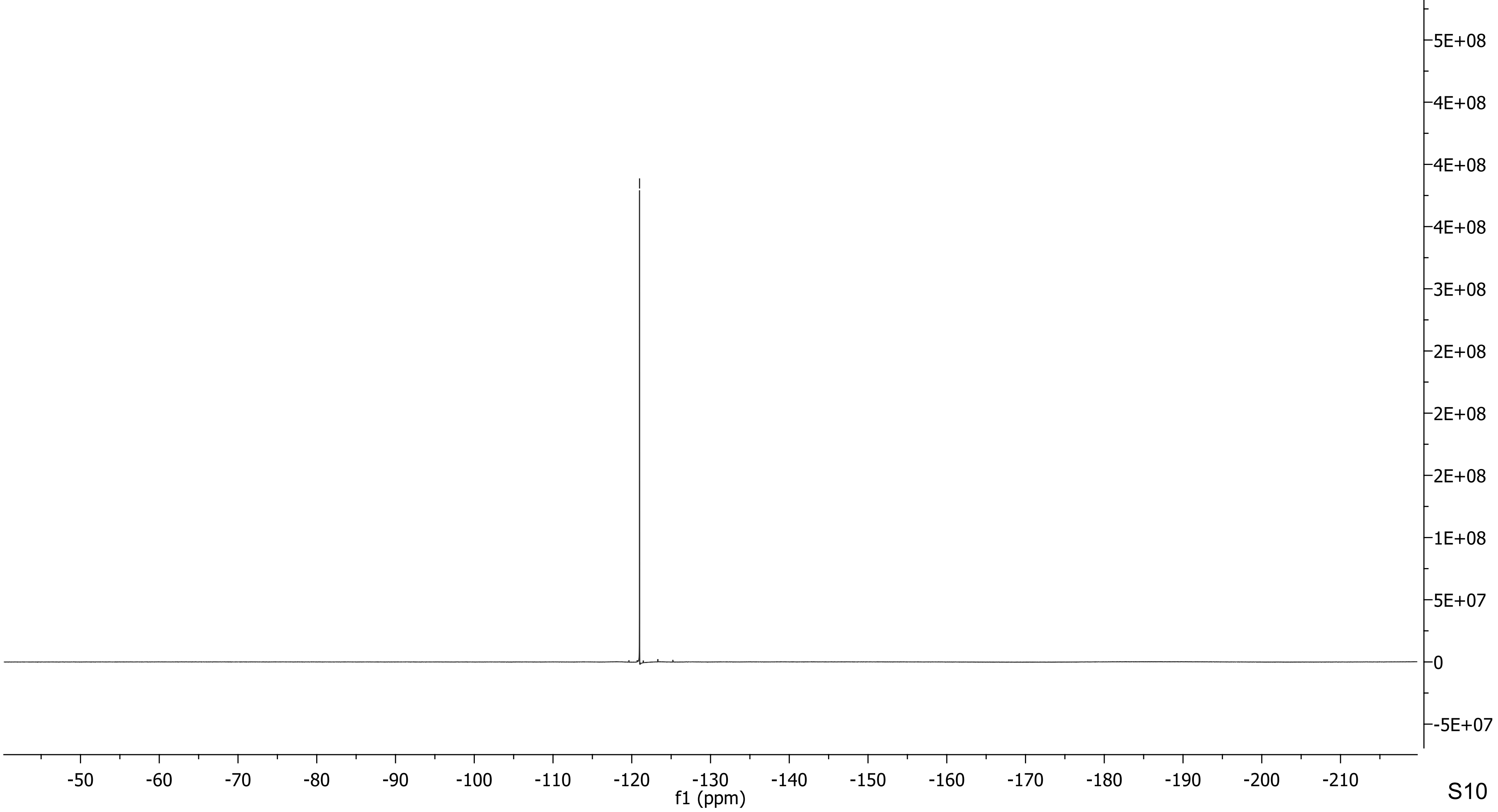
-120.99
7E+08
6E+08
6E+08
6E+08
5E+08
4E+08
4E+08
4E+08
3E+08
2E+08
2E+08
2E+08
1E+08
5E+07
0
-5E+07
-50
-60
-70
-80
-90
-100
-110
-120
-130
-140
-150
-160
-170
-180
-190
-200
-210
f1 (ppm)

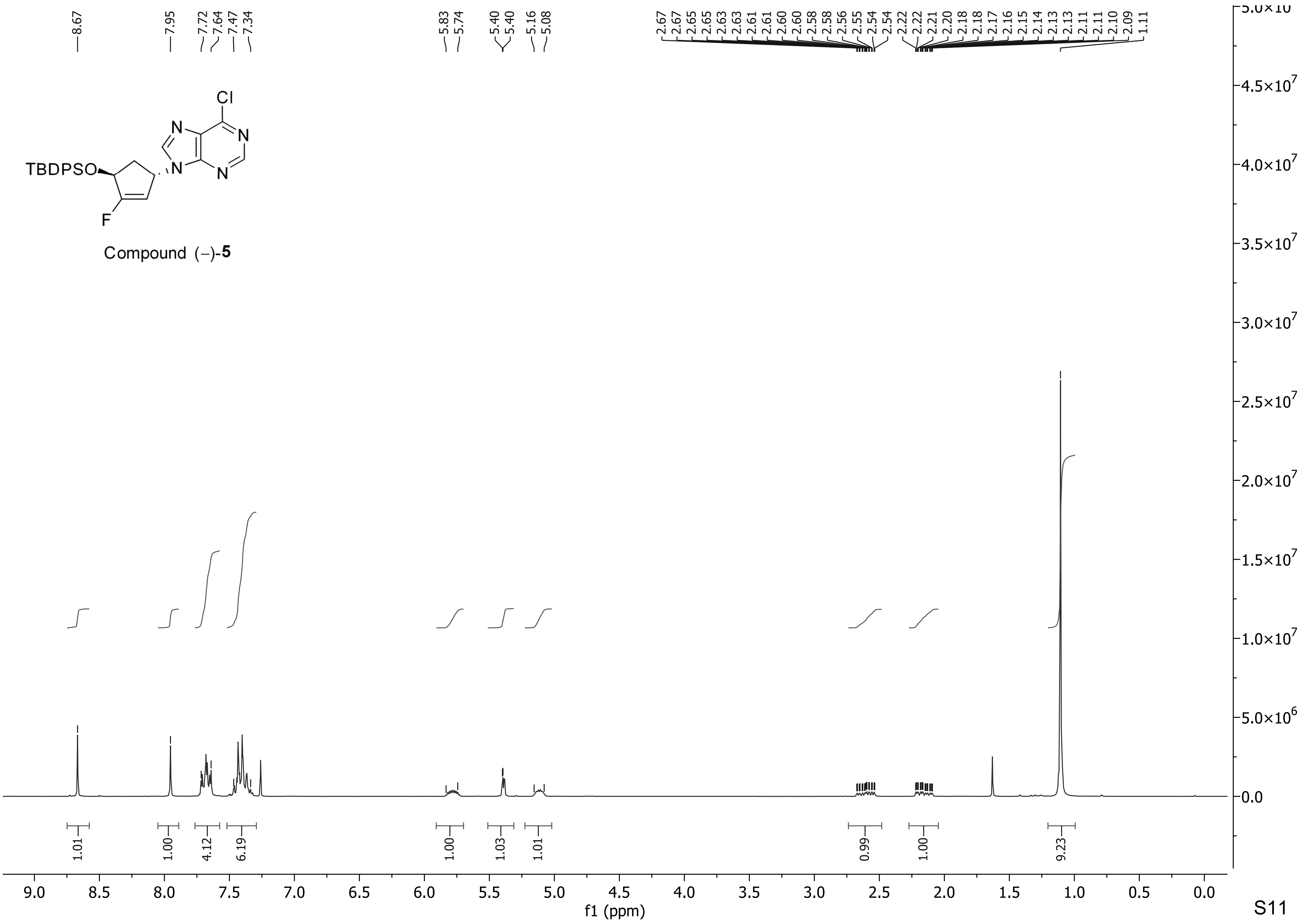
8.67
7.95
7.72
7.64
7.47
7.34
5.83
5.74
5.40
5.40
5.16
5.08
2.67 2.67 2.65 2.65 2.63 2.63 2.61 2.61 2.60 2.60 2.58 2.58 2.56 2.55 2.54 2.54
2.22 2.22 2.21 2.20 2.18 2.18 2.17 2.16 2.15 2.14 2.13 2.13 2.11 2.11 2.10 2.09
1.11
Cl
N
TBDPSO
F
Compound (−)-5
1.01 1.00 4.12 6.19 1.00 1.03 1.01 0.99 1.00 9.23
9.0 8.5 8.0 7.5 7.0 6.5 6.0 5.5 5.0 4.5 4.0 3.5 3.0 2.5 2.0 1.5 1.0 0.5 0.0
f1 (ppm)
5.0×10^7
4.5×10^7
4.0×10^7
3.5×10^7
3.0×10^7
2.5×10^7
2.0×10^7
1.5×10^7
1.0×10^7
5.0×10^6
0.0

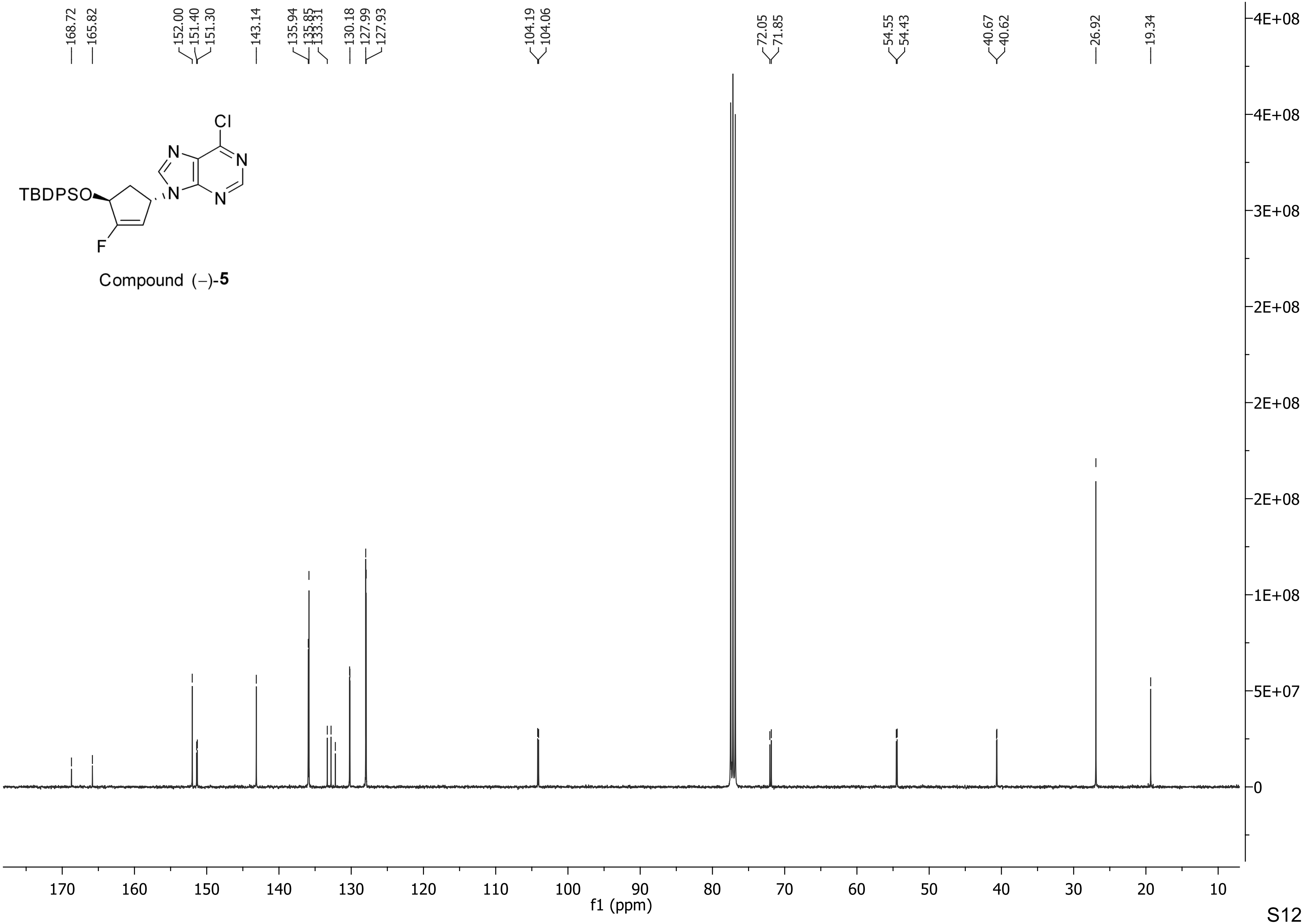
168.72
165.82
152.00
151.40
151.30
143.14
135.94
135.85
133.31
130.18
127.99
127.93
104.19
104.06
72.05
71.85
54.55
54.43
40.67
40.62
26.92
19.34
Cl
N
N
N
N
TBDPSO
F
Compound (–)-5
4E+08
4E+08
3E+08
2E+08
2E+08
2E+08
1E+08
5E+07
0
170 160 150 140 130 120 110 100 90 80 70 60 50 40 30 20 10
f1 (ppm)

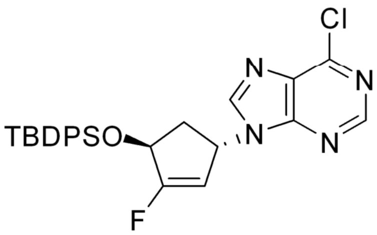
TBDPSO
Cl
N
N
N
N
F

Compound (–)-**5**

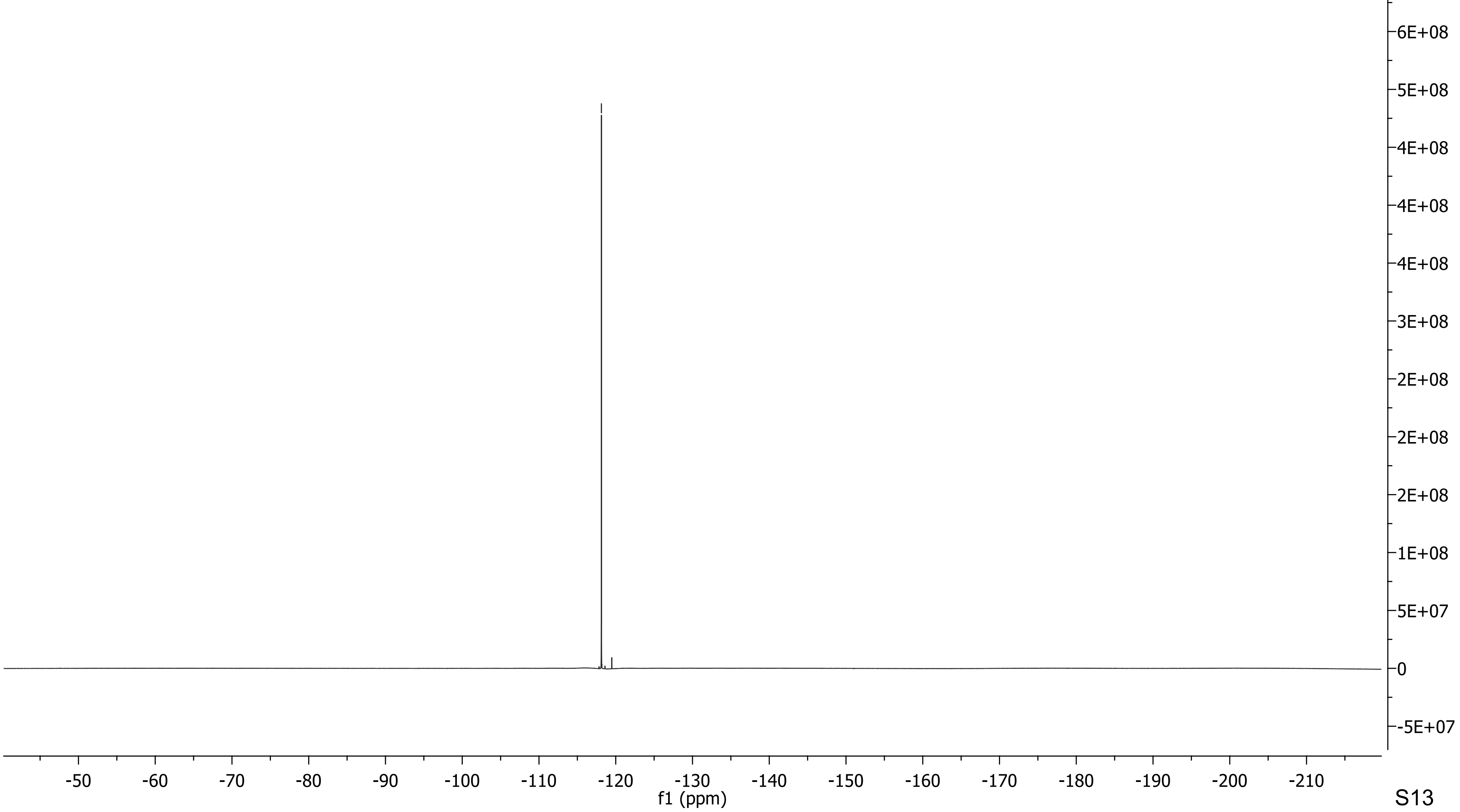
-118.13
8E+08
7E+08
6E+08
6E+08
6E+08
5E+08
4E+08
4E+08
4E+08
3E+08
2E+08
2E+08
2E+08
1E+08
5E+07
0
-5E+07
-50
-60
-70
-80
-90
-100
-110
-120
-130
-140
-150
-160
-170
-180
-190
-200
-210
f1 (ppm)

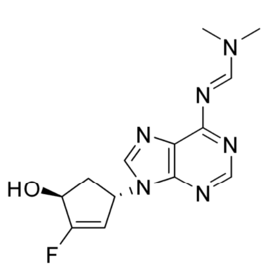
N
N
N
N
N
N
HO
F

Compound (–)-**6**

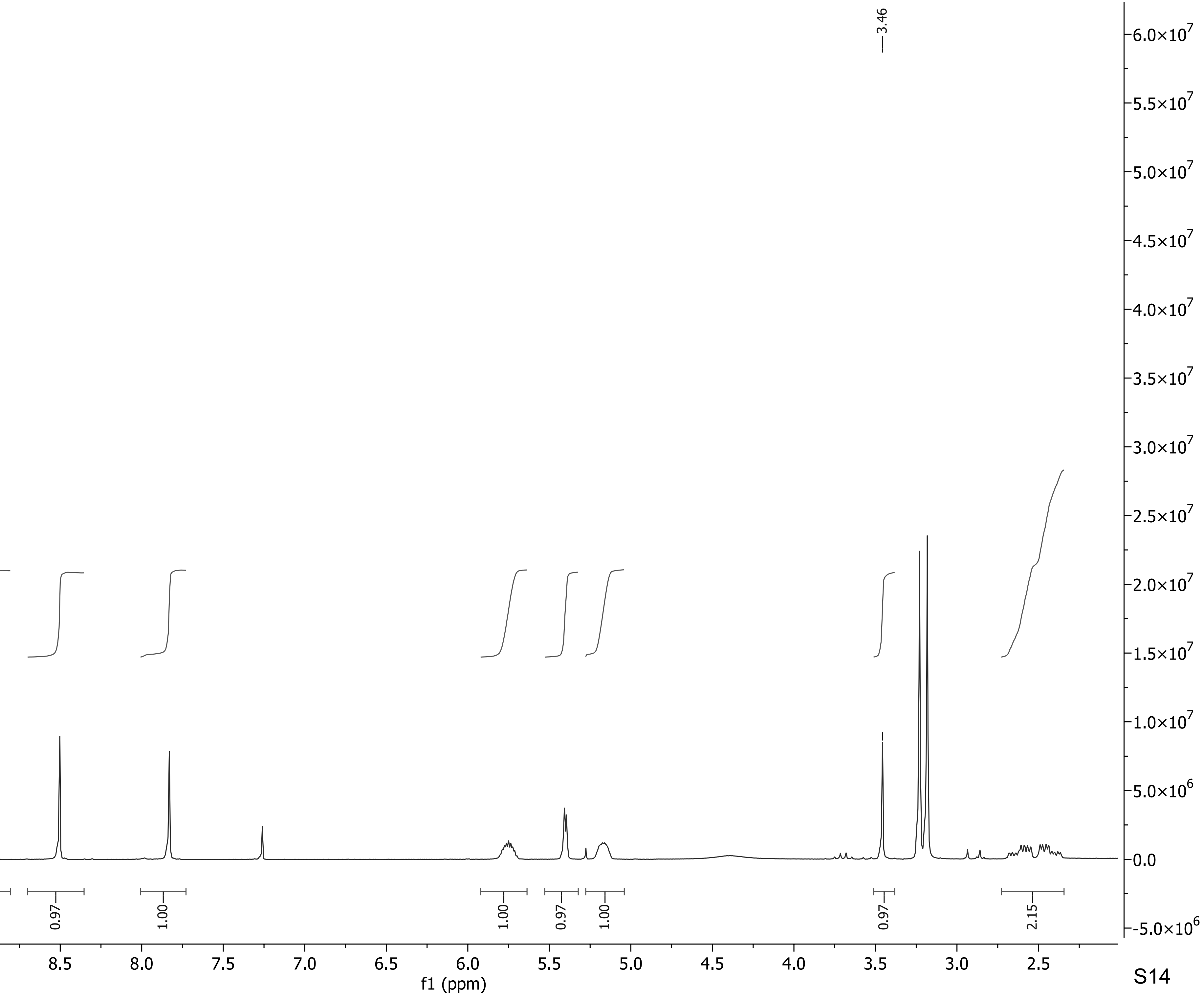
3.46
0.99
0.97
1.00
1.00
0.97
1.00
0.97
2.15
10.0
9.5
9.0
8.5
8.0
7.5
7.0
6.5
6.0
5.5
5.0
4.5
4.0
3.5
3.0
2.5
f1 (ppm)
6.0×10^7
5.5×10^7
5.0×10^7
4.5×10^7
4.0×10^7
3.5×10^7
3.0×10^7
2.5×10^7
2.0×10^7
1.5×10^7
1.0×10^7
5.0×10^6
0.0
-5.0×10^6

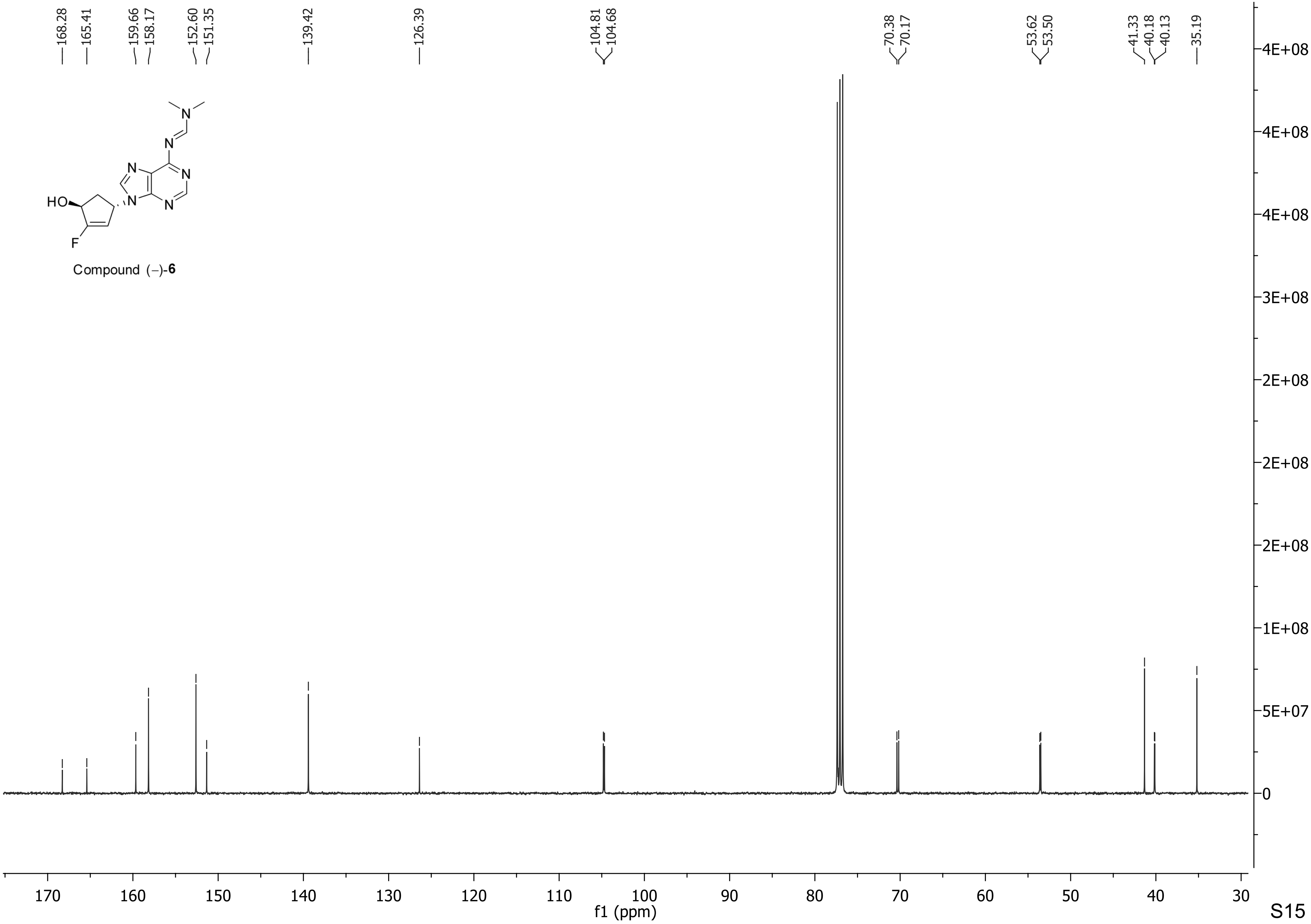
168.28
165.41
159.66
158.17
152.60
151.35
139.42
126.39
104.81
104.68
70.38
70.17
53.62
53.50
41.33
40.18
40.13
35.19
HO
F
N
f1 (ppm)
Compound (−)-**6**

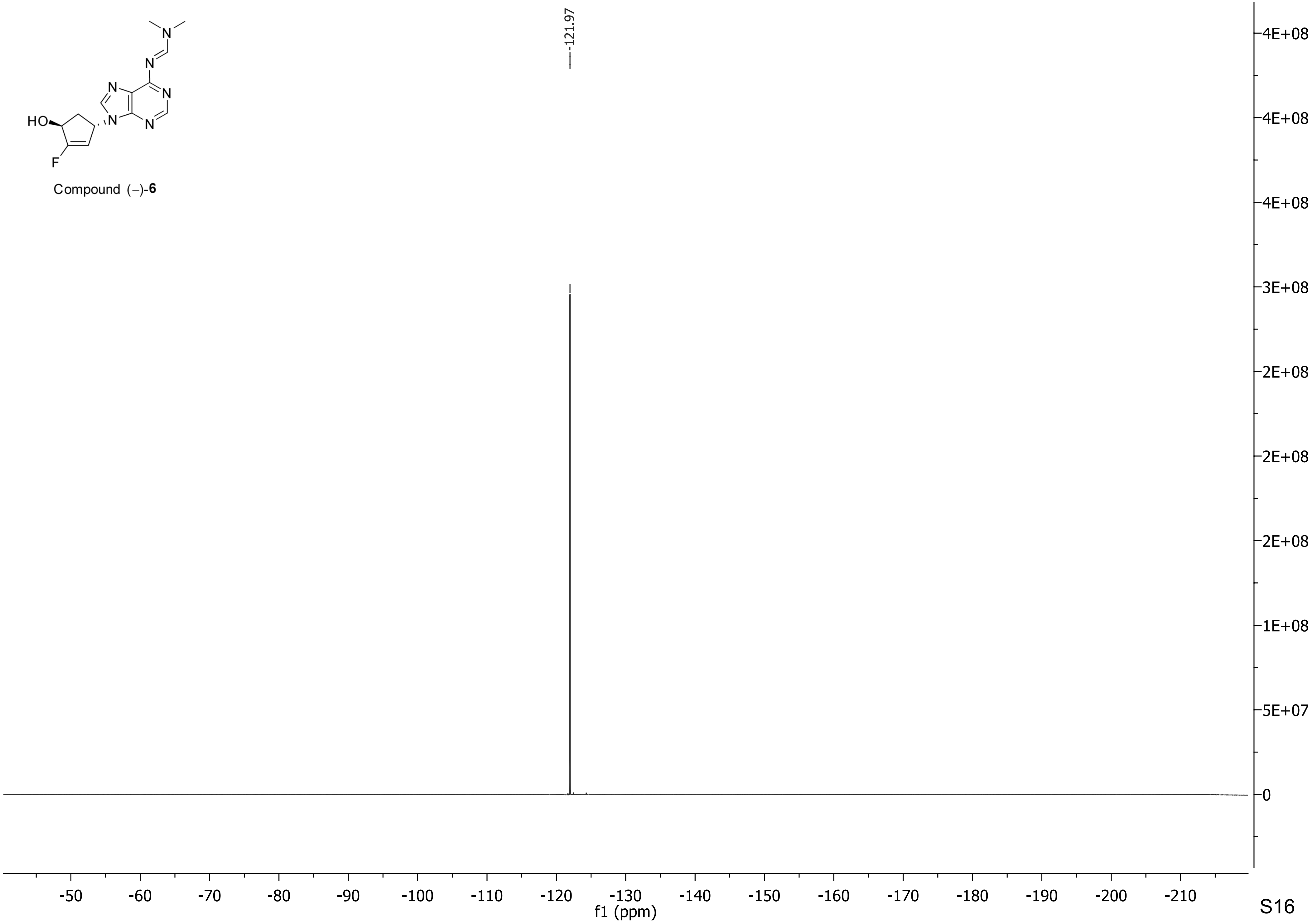

Compound (–)-6
HO
F
N
-121.97
4E+08
3E+08
2E+08
1E+08
5E+07
0
-50 -60 -70 -80 -90 -100 -110 -120 -130 -140 -150 -160 -170 -180 -190 -200 -210
f1 (ppm)

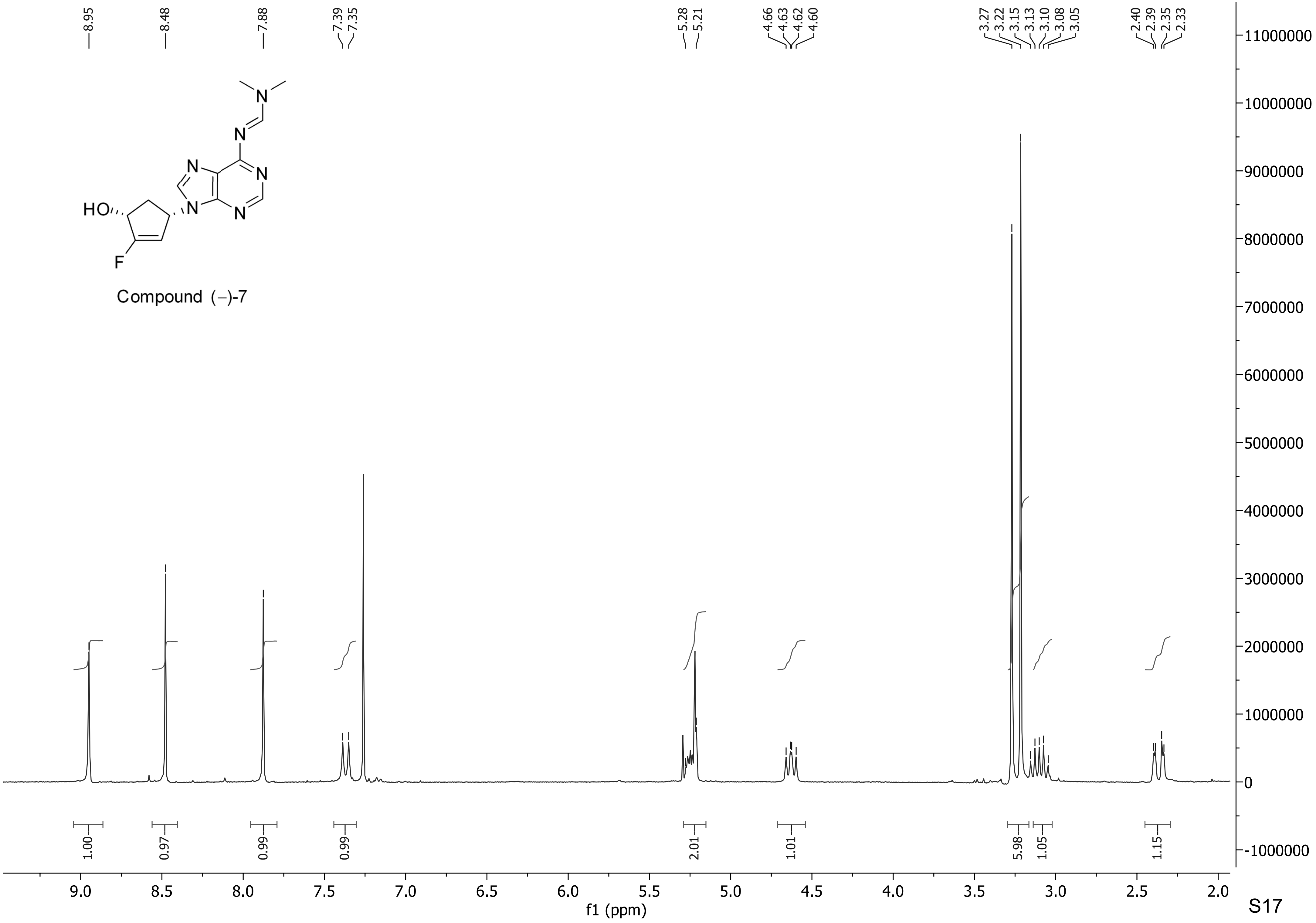
8.95
8.48
7.88
7.39
7.35
5.28
5.21
4.66
4.63
4.62
4.60
3.27
3.22
3.15
3.13
3.10
3.08
3.05
2.40
2.39
2.35
2.33
HO
F
N
Compound (−)-7
1.00
0.97
0.99
0.99
2.01
1.01
5.98
1.05
1.15
f1 (ppm)

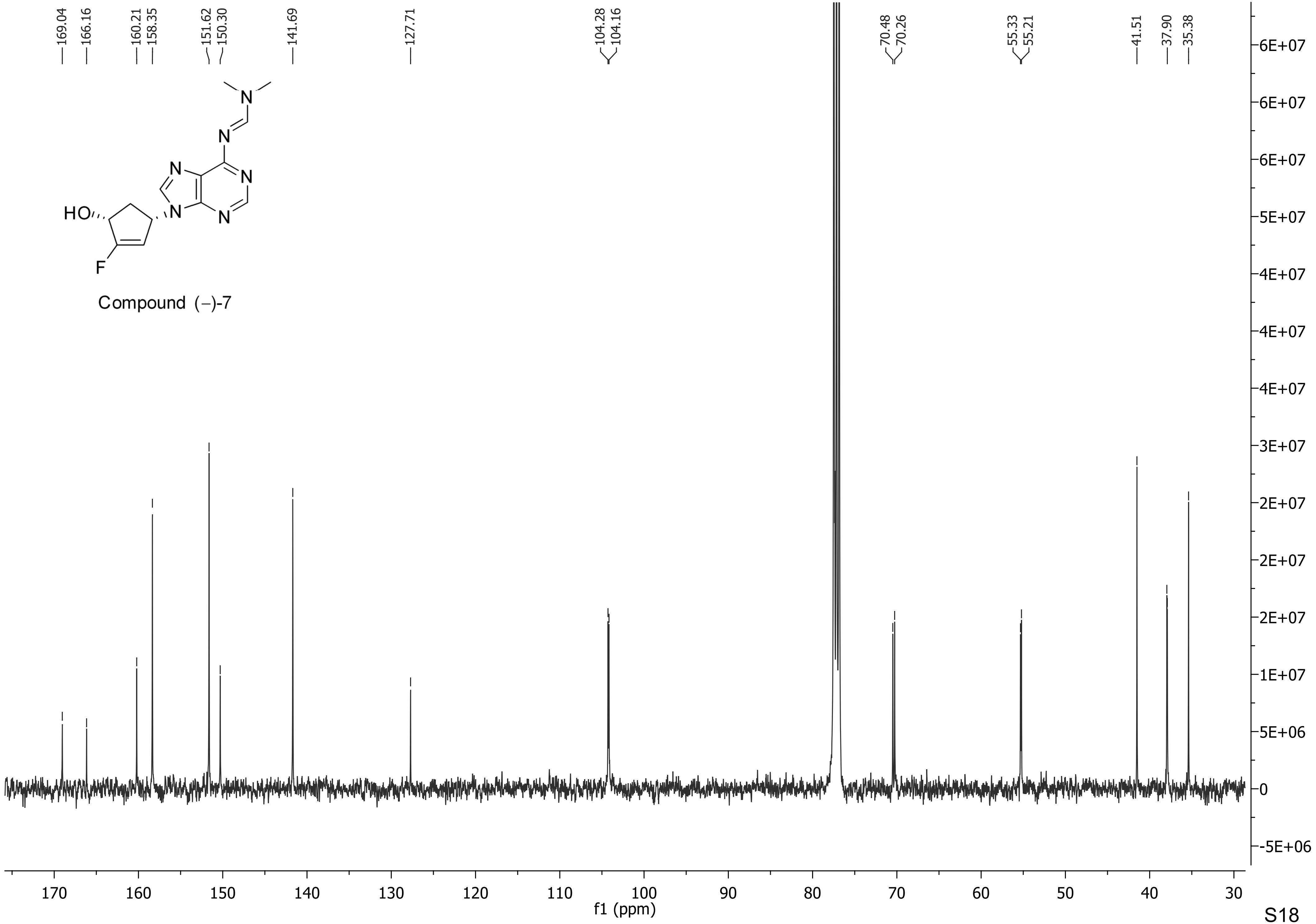
169.04
166.16
160.21
158.35
151.62
150.30
141.69
127.71
104.28
104.16
70.48
70.26
55.33
55.21
41.51
37.90
35.38
HO
F
N
Compound (–)-7
6E+07
5E+07
4E+07
3E+07
2E+07
1E+07
5E+06
0
-5E+06
170
160
150
140
130
120
110
100
90
80
70
60
50
40
30
f1 (ppm)

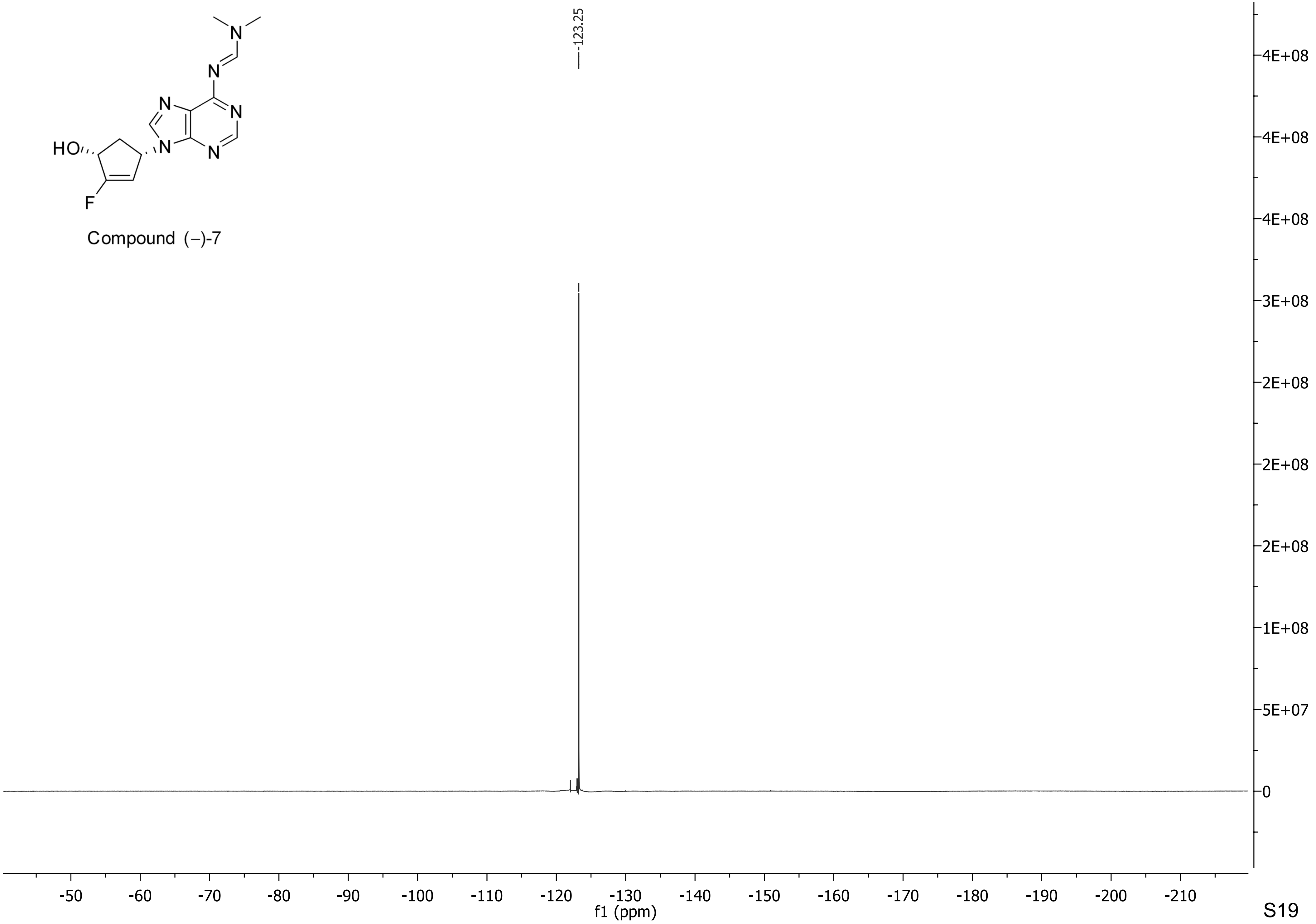
HO
F
N
N
N
N
N
N
Compound (–)-7
-123.25
4E+08
4E+08
4E+08
3E+08
2E+08
2E+08
2E+08
1E+08
5E+07
0
-50
-60
-70
-80
-90
-100
-110
-120
-130
-140
-150
-160
-170
-180
-190
-200
-210
f1 (ppm)

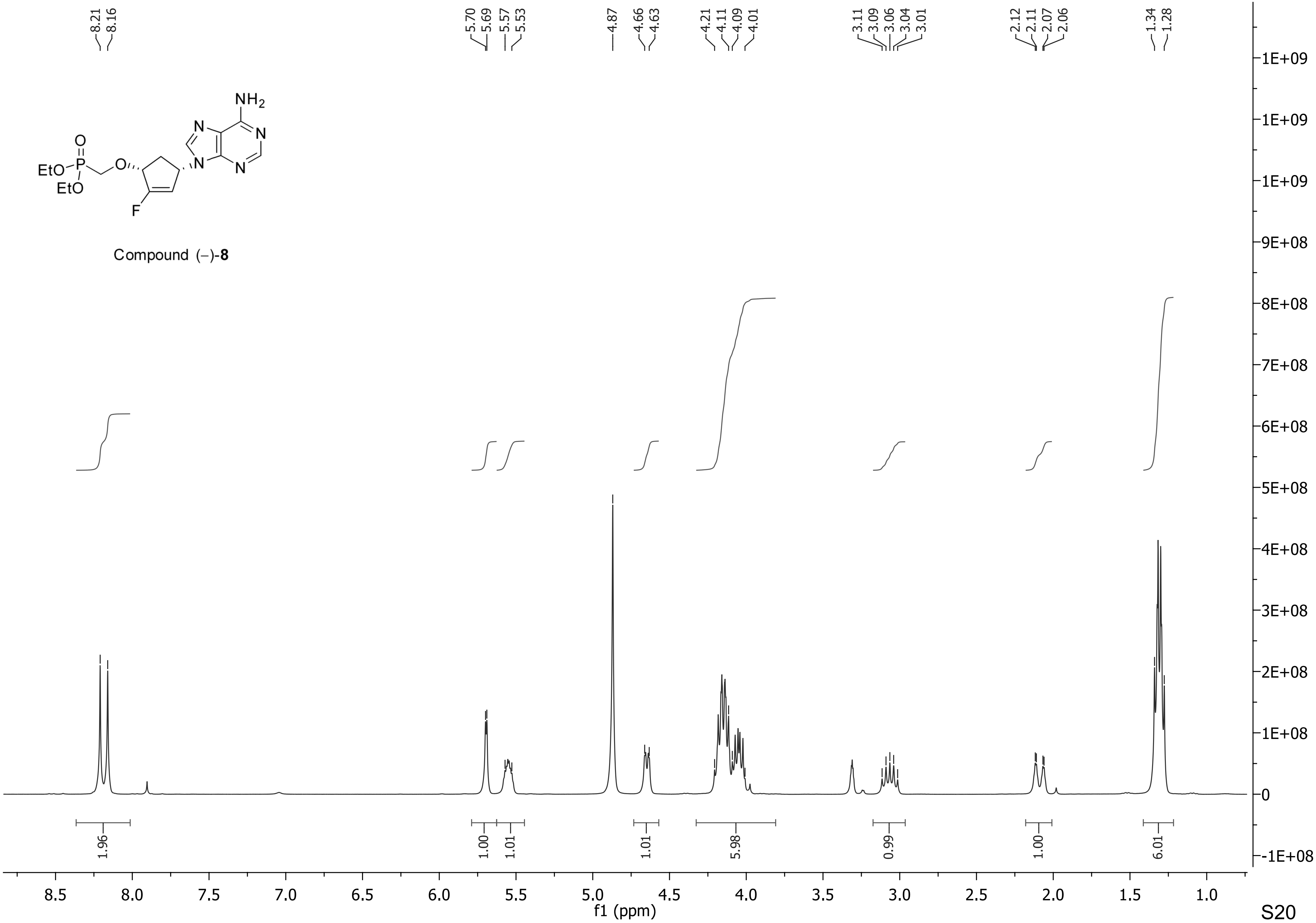
8.21
8.16
5.70
5.69
5.57
5.53
4.87
4.66
4.63
4.21
4.11
4.09
4.01
3.11
3.09
3.06
3.04
3.01
2.12
2.11
2.07
2.06
1.34
1.28
NH2
N
O
EtO
P
O
EtO
F
1.96
1.00
1.01
1.01
5.98
0.99
1.00
6.01
f1 (ppm)

Compound (–)-**8**

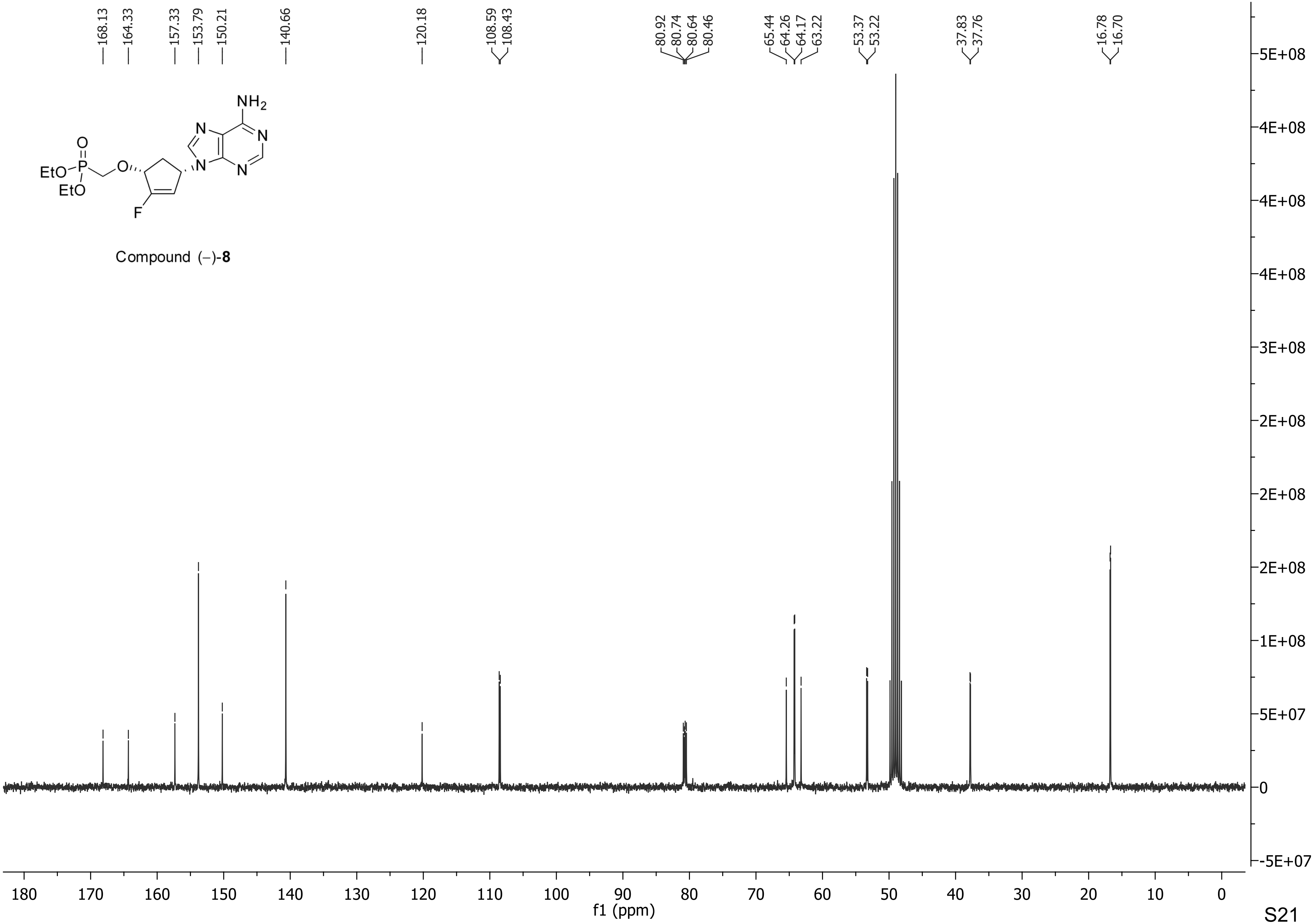

168.13
164.33
157.33
153.79
150.21
140.66
120.18
108.59
108.43
80.92
80.74
80.64
80.46
65.44
64.26
64.17
63.22
53.37
53.22
37.83
37.76
16.78
16.70
NH2
N
N
N
N
O
EtO
P
O
EtO
F
Compound (–)-8
5E+08
4E+08
4E+08
4E+08
3E+08
2E+08
2E+08
2E+08
1E+08
5E+07
0
-5E+07
180
170
160
150
140
130
120
110
100
90
80
70
60
50
40
30
20
10
0
f1 (ppm)

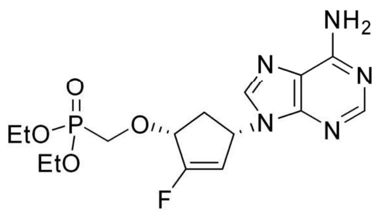

Compound (–)-**8**

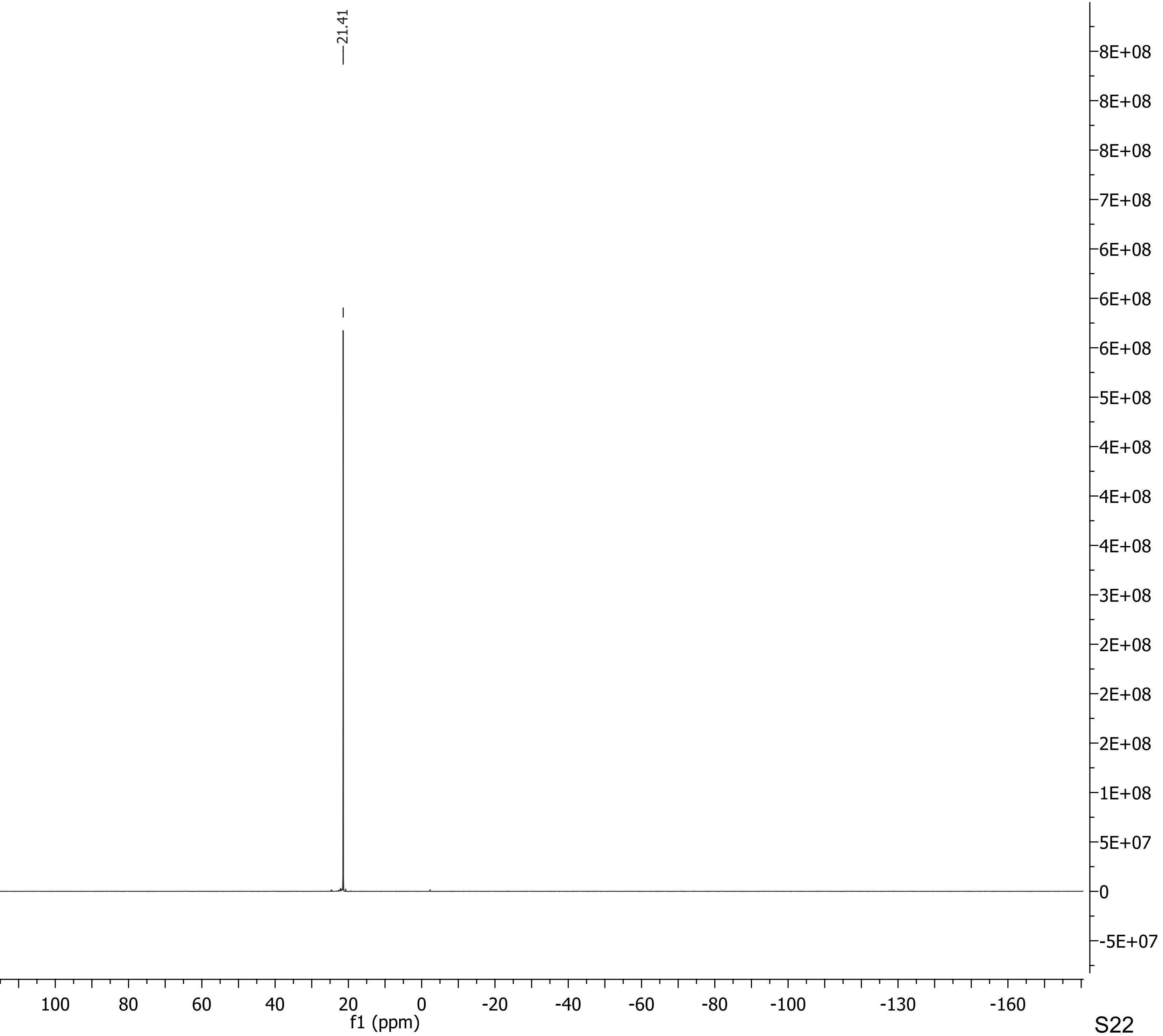

21.41
8E+08
7E+08
6E+08
5E+08
4E+08
3E+08
2E+08
1E+08
5E+07
0
-5E+07
200
180
160
140
120
100
80
60
40
20
0
-20
-40
-60
-80
-100
-130
-160
f1 (ppm)

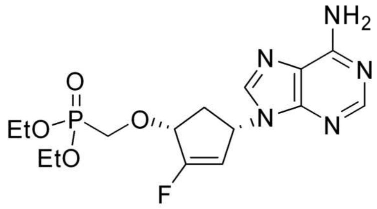
NH2
N
N
N
N
O
EtO
P
O
EtO
F

Compound (–)-**8**

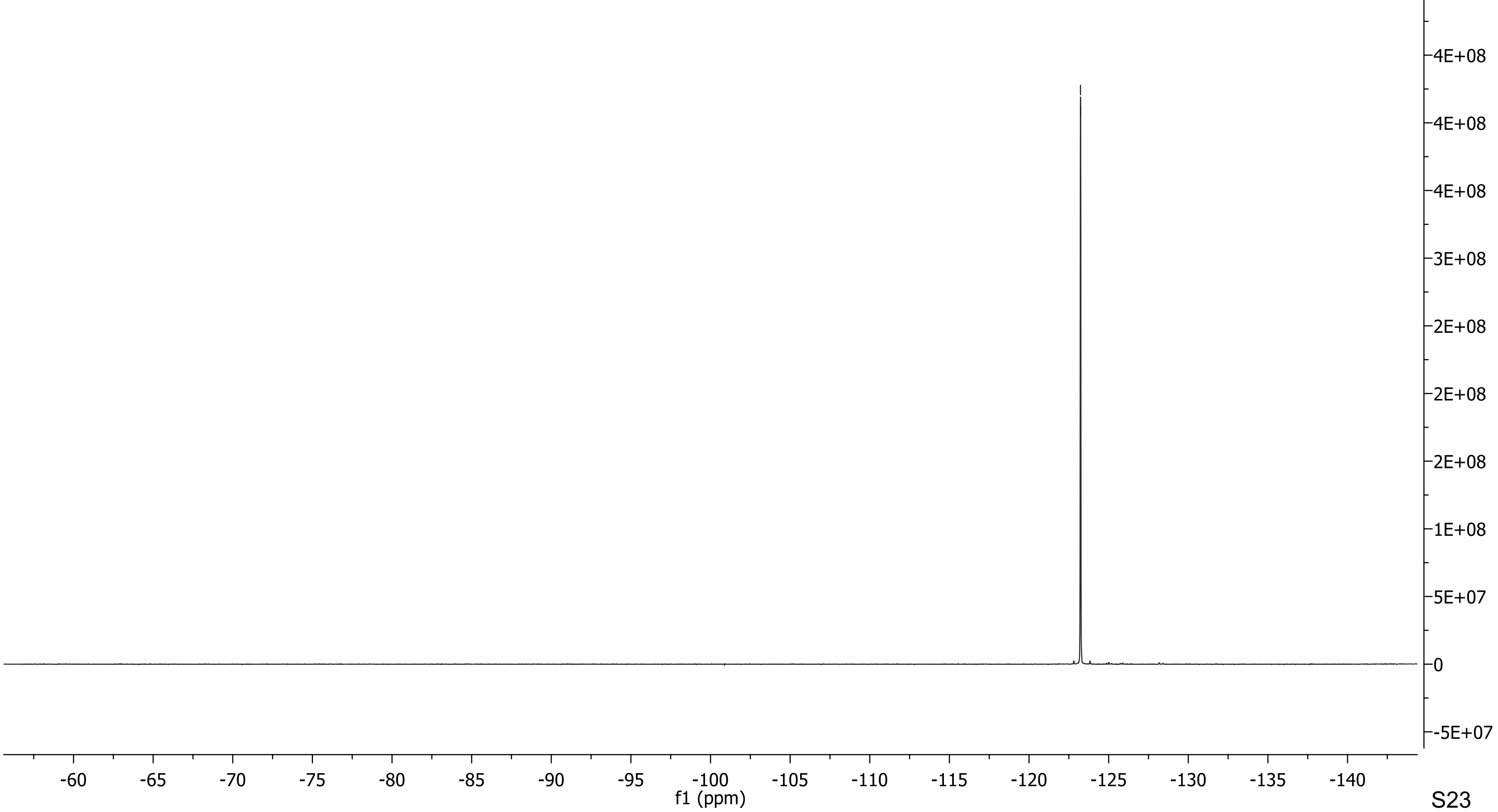
-123.23
-123.24
f1 (ppm)
-60
-65
-70
-75
-80
-85
-90
-95
-100
-105
-110
-115
-120
-125
-130
-135
-140
6E+08
6E+08
6E+08
5E+08
4E+08
4E+08
4E+08
3E+08
2E+08
2E+08
2E+08
1E+08
5E+07
0
-5E+07

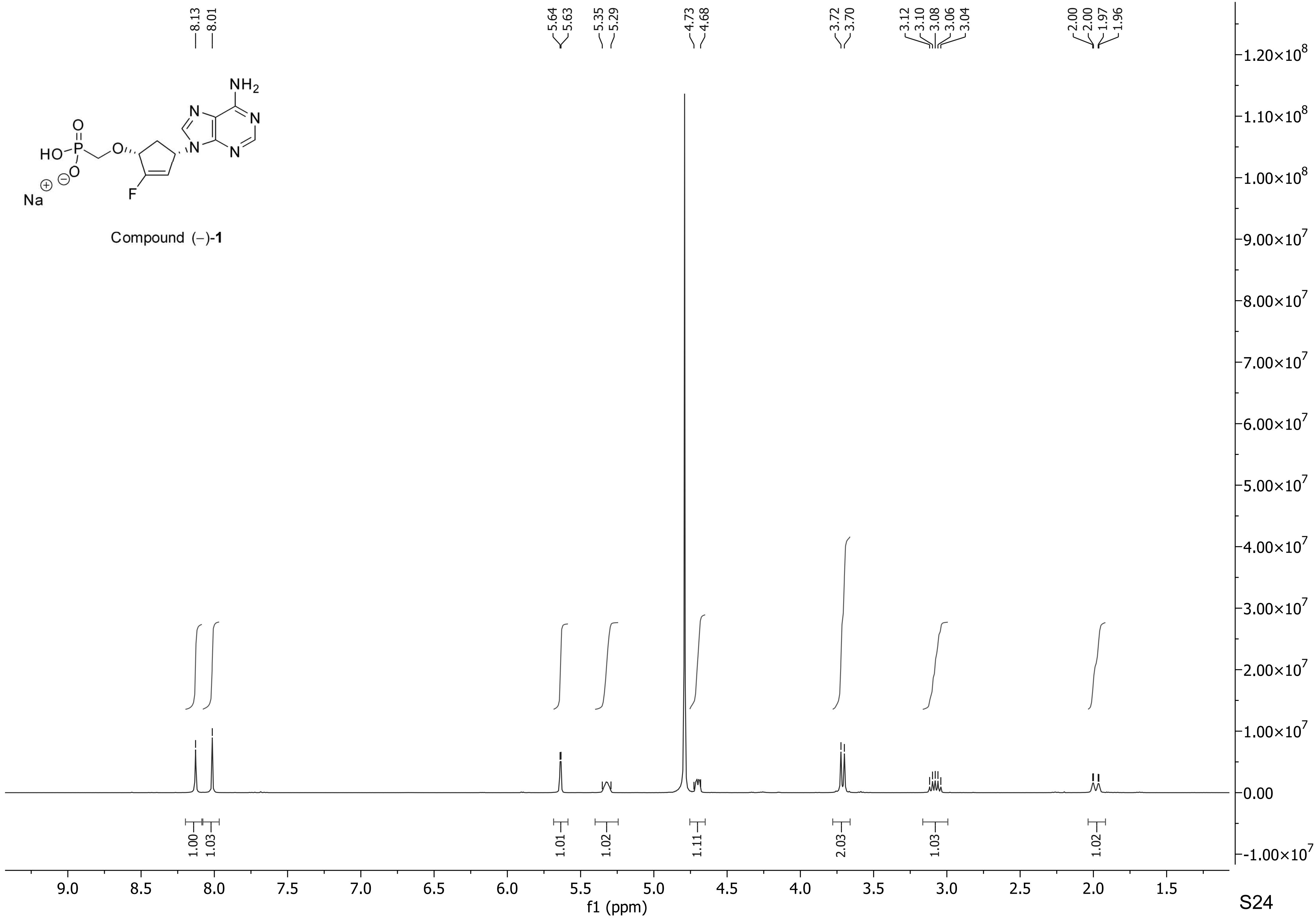
8.13
8.01
5.64
5.63
5.35
5.29
4.73
4.68
3.72
3.70
3.12
3.10
3.08
3.06
3.04
2.00
2.00
1.97
1.96
NH_2
N
N
N
N
O
HO
P
O
O
F
Na
Compound (–)-**1**
1.00
1.03
1.01
1.02
1.11
2.03
1.03
1.02
9.0
8.5
8.0
7.5
7.0
6.5
6.0
5.5
5.0
4.5
4.0
3.5
3.0
2.5
2.0
1.5
f1 (ppm)
1.20×10^8
1.10×10^8
1.00×10^8
9.00×10^7
8.00×10^7
7.00×10^7
6.00×10^7
5.00×10^7
4.00×10^7
3.00×10^7
2.00×10^7
1.00×10^7
0.00
-1.00×10^7

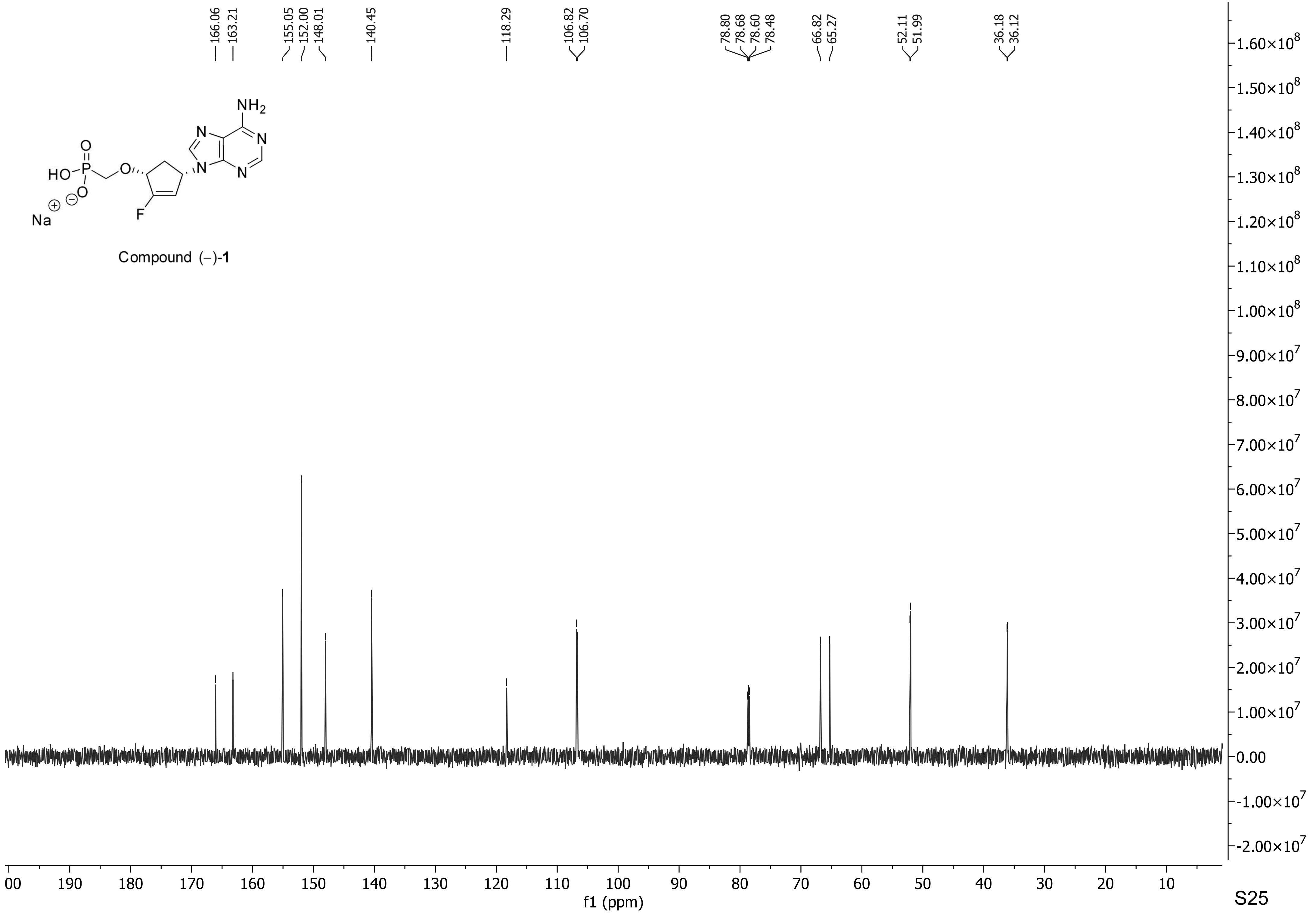
166.06
163.21
155.05
152.00
148.01
140.45
118.29
106.82
106.70
78.80
78.68
78.60
78.48
66.82
65.27
52.11
51.99
36.18
36.12
NH2
N
N
N
N
O
HO
P
O
O
Na
F
Compound (–)-1
1.60×10^8
1.50×10^8
1.40×10^8
1.30×10^8
1.20×10^8
1.10×10^8
1.00×10^8
9.00×10^7
8.00×10^7
7.00×10^7
6.00×10^7
5.00×10^7
4.00×10^7
3.00×10^7
2.00×10^7
1.00×10^7
0.00
-1.00×10^7
-2.00×10^7
00
190
180
170
160
150
140
130
120
110
100
90
80
70
60
50
40
30
20
10
f1 (ppm)

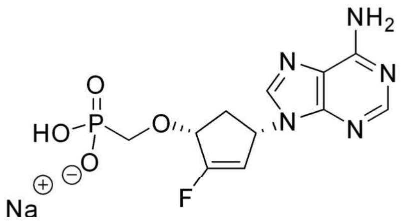
NH2
N
N
N
N
O
HO
P
O
O
Na
F

Compound (–)-**1**

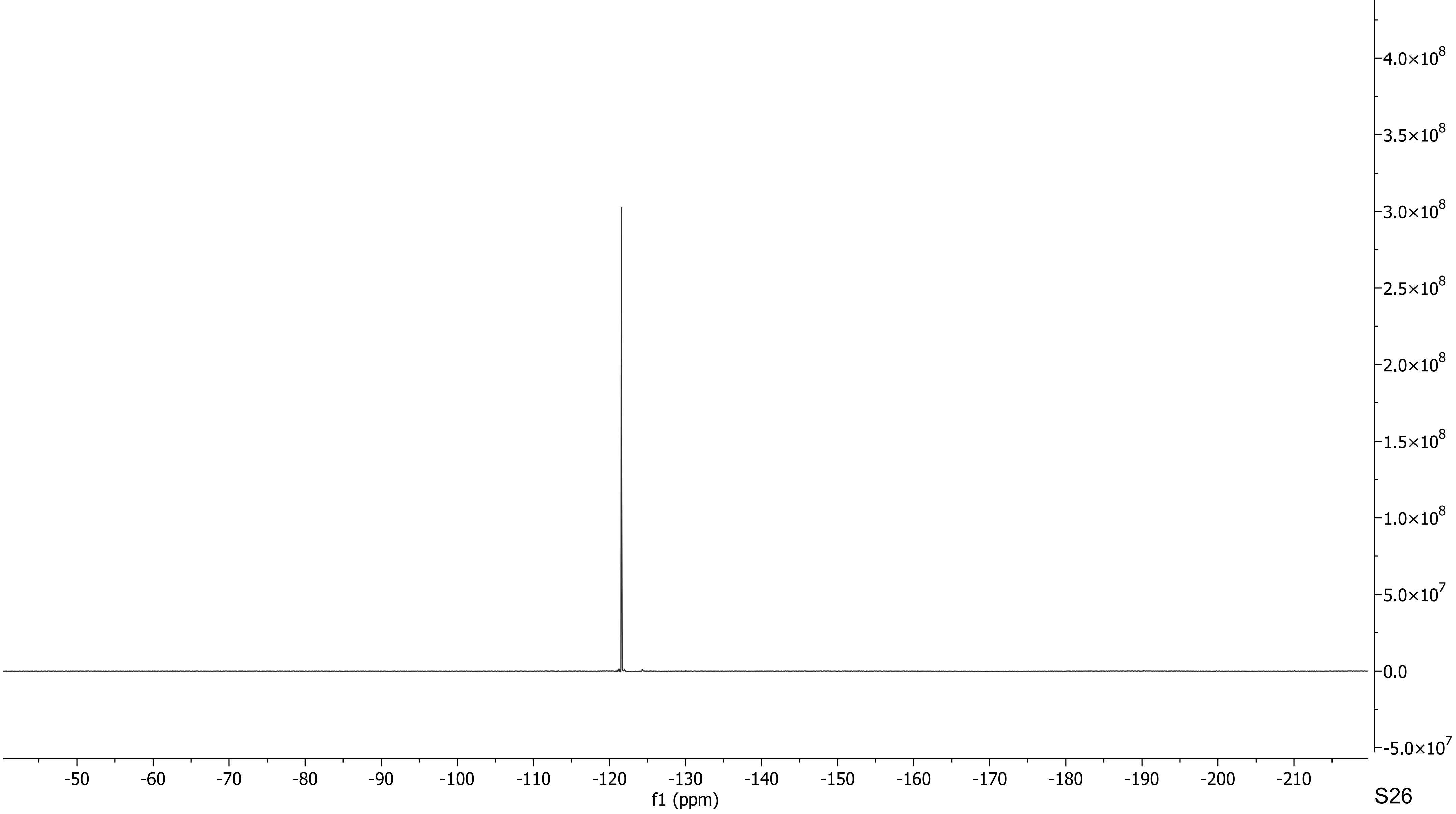
5.5×10^8
5.0×10^8
4.5×10^8
4.0×10^8
3.5×10^8
3.0×10^8
2.5×10^8
2.0×10^8
1.5×10^8
1.0×10^8
5.0×10^7
0.0
-5.0×10^7
-50 -60 -70 -80 -90 -100 -110 -120 -130 -140 -150 -160 -170 -180 -190 -200 -210
f1 (ppm)

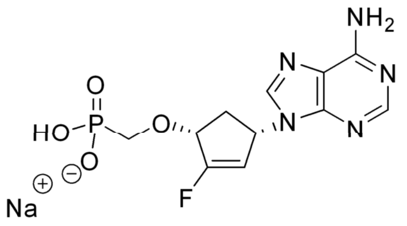
NH2
N
N
N
N
O
HO
P
O
O
Na
F

Compound (–)-**1**

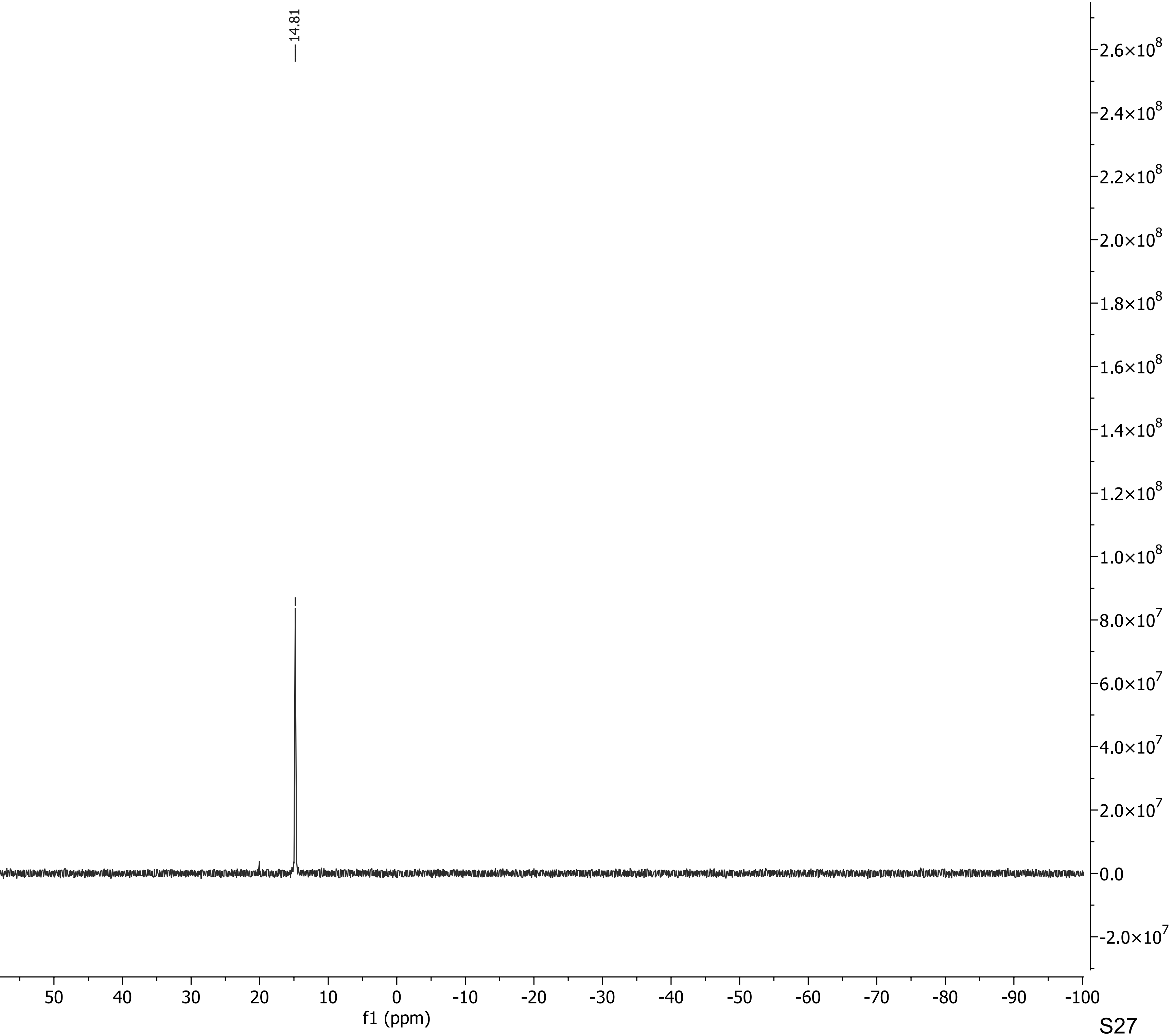
14.81
2.6×10^8
2.4×10^8
2.2×10^8
2.0×10^8
1.8×10^8
1.6×10^8
1.4×10^8
1.2×10^8
1.0×10^8
8.0×10^7
6.0×10^7
4.0×10^7
2.0×10^7
0.0
-2.0×10^7
90
80
70
60
50
40
30
20
10
0
-10
-20
-30
-40
-50
-60
-70
-80
-90
-100
f1 (ppm)

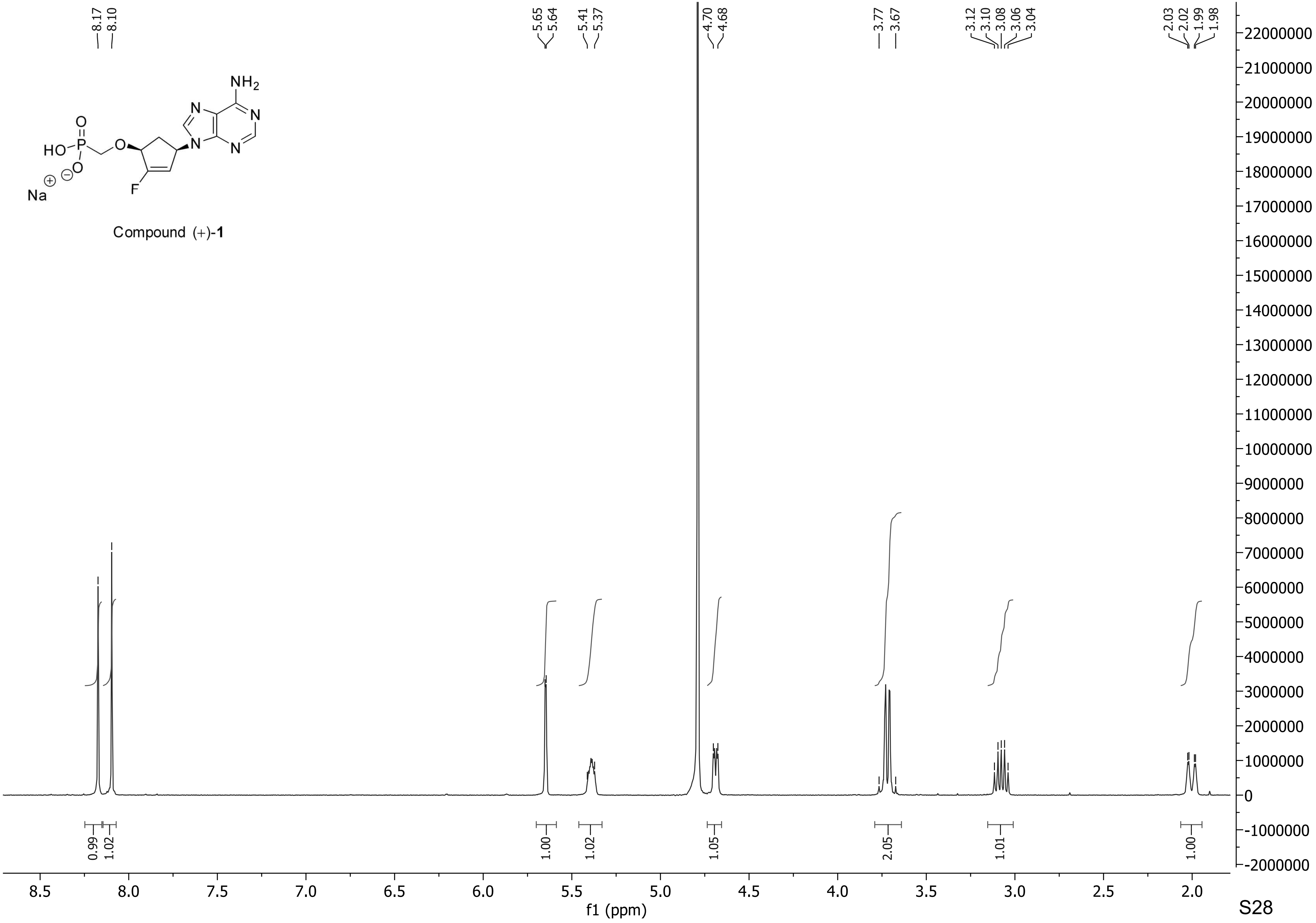
8.17
8.10
5.65
5.64
5.41
5.37
4.70
4.68
3.77
3.67
3.12
3.10
3.08
3.06
3.04
2.03
2.02
1.99
1.98
NH2
N
N
N
N
O
HO
P
O
O
Na
F
Compound (+)-1
0.99
1.02
1.00
1.02
1.05
2.05
1.01
1.00
8.5
8.0
7.5
7.0
6.5
6.0
5.5
5.0
4.5
4.0
3.5
3.0
2.5
2.0
f1 (ppm)
22000000
21000000
20000000
19000000
18000000
17000000
16000000
15000000
14000000
13000000
12000000
11000000
10000000
9000000
8000000
7000000
6000000
5000000
4000000
3000000
2000000
1000000
0
-1000000
-2000000

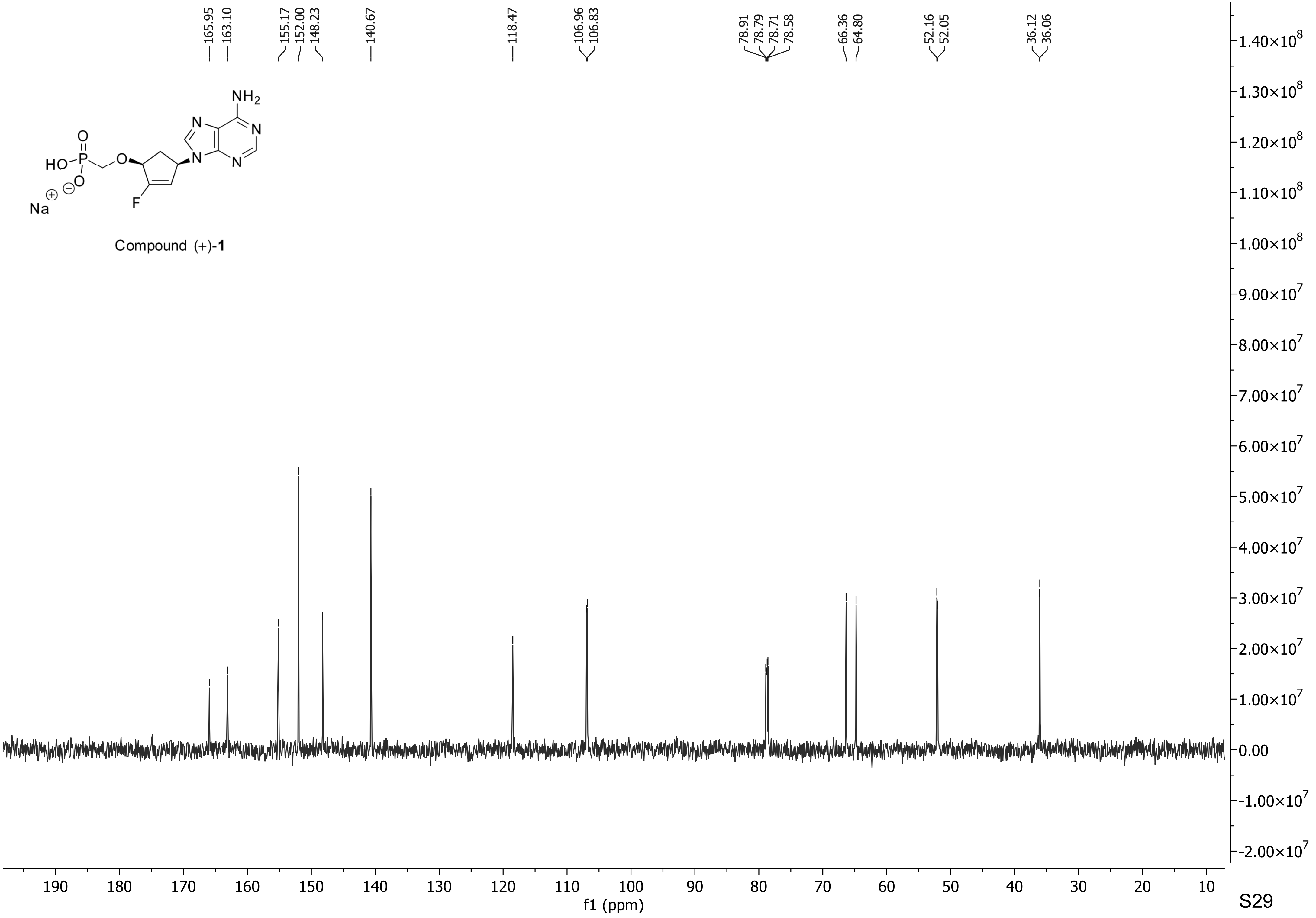

165.95
163.10
155.17
152.00
148.23
140.67
118.47
106.96
106.83
78.91
78.79
78.71
78.58
66.36
64.80
52.16
52.05
36.12
36.06
Compound (+)-1
f1 (ppm)

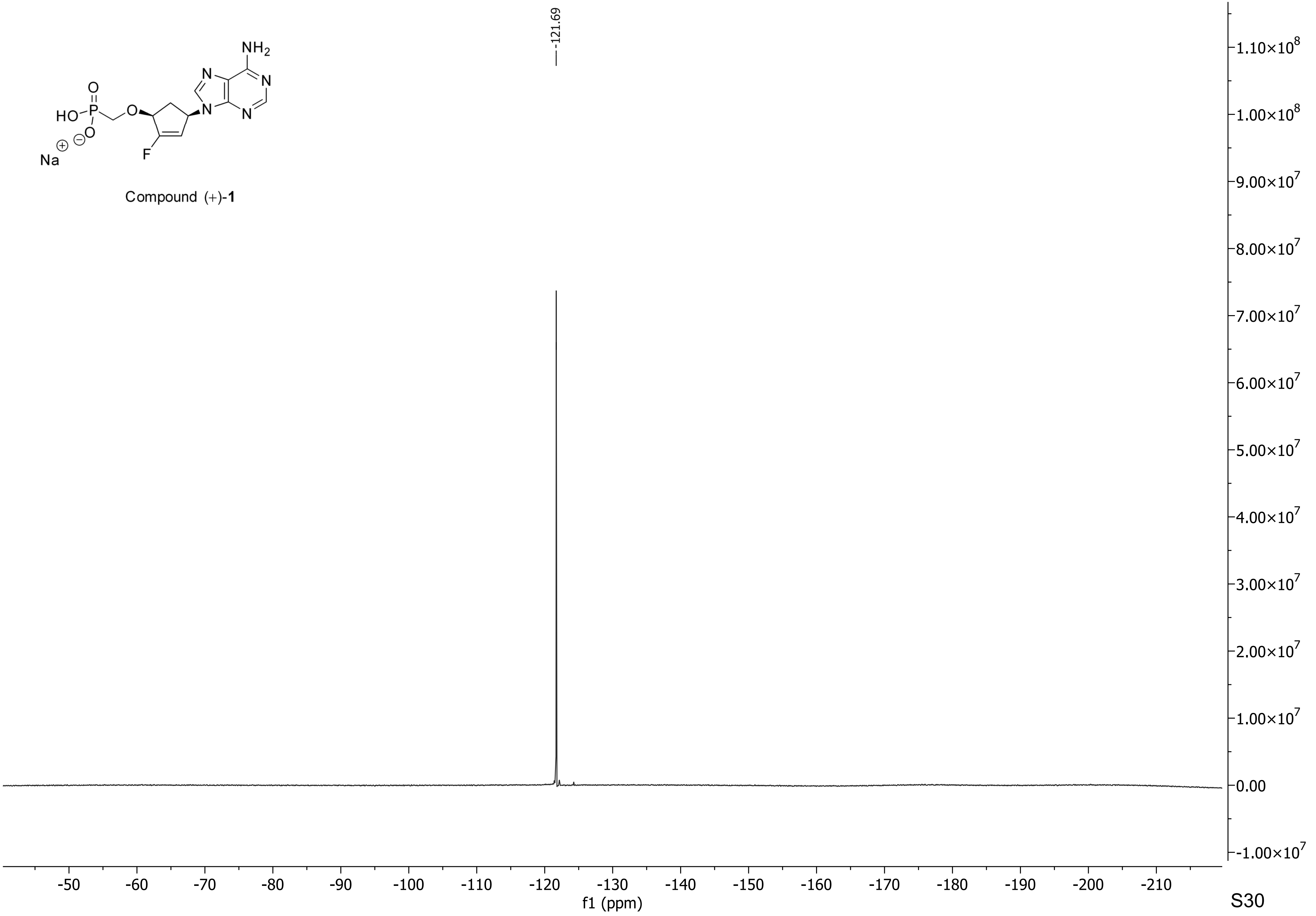

NH2
O
HO
P
O
N
N
N
N
Na
F
Compound (+)-1
-121.69
f1 (ppm)
-50
-60
-70
-80
-90
-100
-110
-120
-130
-140
-150
-160
-170
-180
-190
-200
-210
1.10×10^8
1.00×10^8
9.00×10^7
8.00×10^7
7.00×10^7
6.00×10^7
5.00×10^7
4.00×10^7
3.00×10^7
2.00×10^7
1.00×10^7
0.00
-1.00×10^7

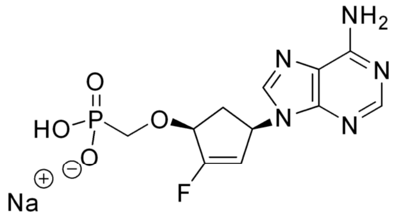

NH2
N
N
N
N
O
HO
P
O
O
Na
F

Compound (+)-**1**

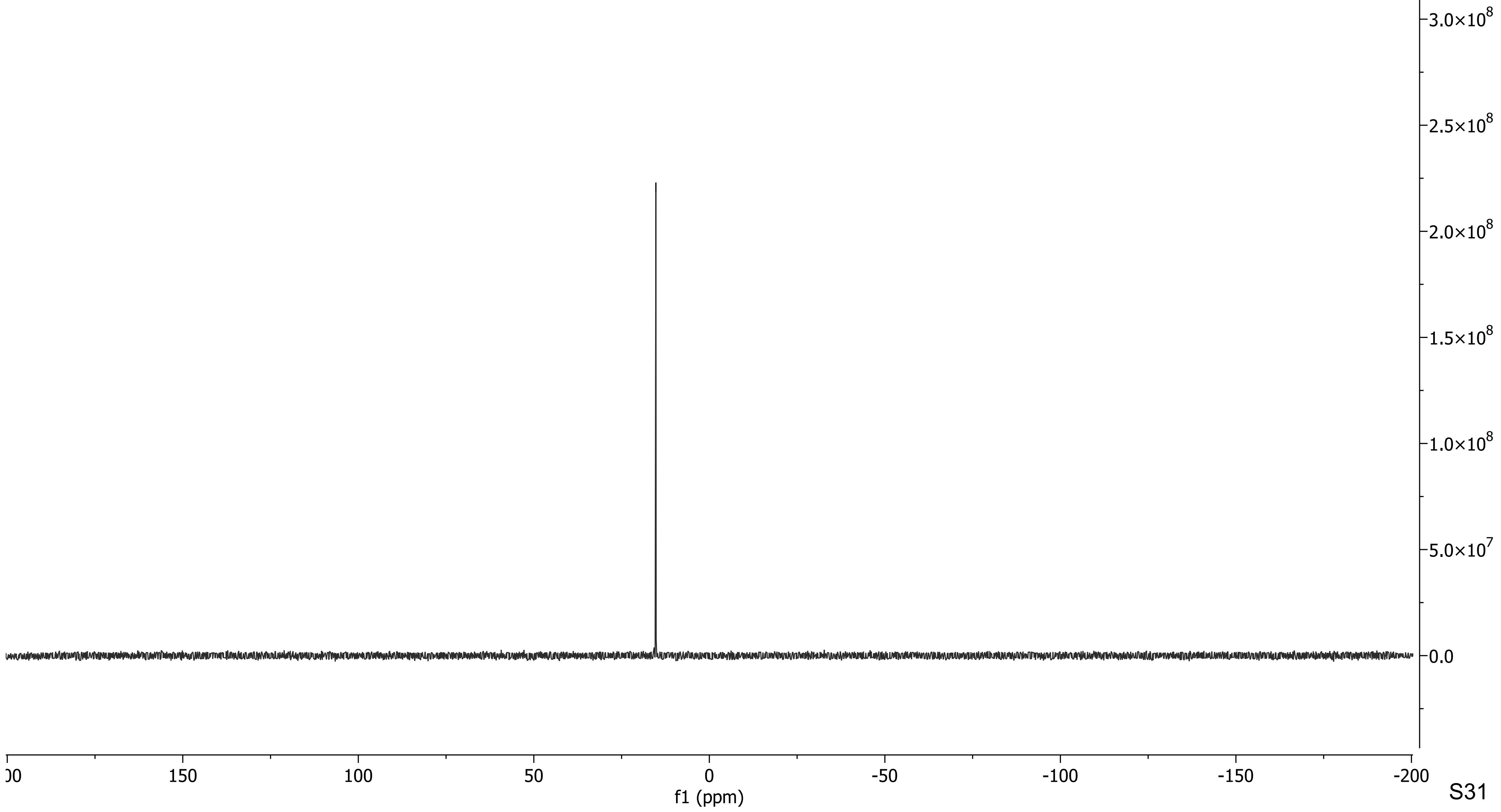

15.22
4.0×10^8
3.5×10^8
3.0×10^8
2.5×10^8
2.0×10^8
1.5×10^8
1.0×10^8
5.0×10^7
0.0
150
100
50
0
-50
-100
-150
-200
f1 (ppm)

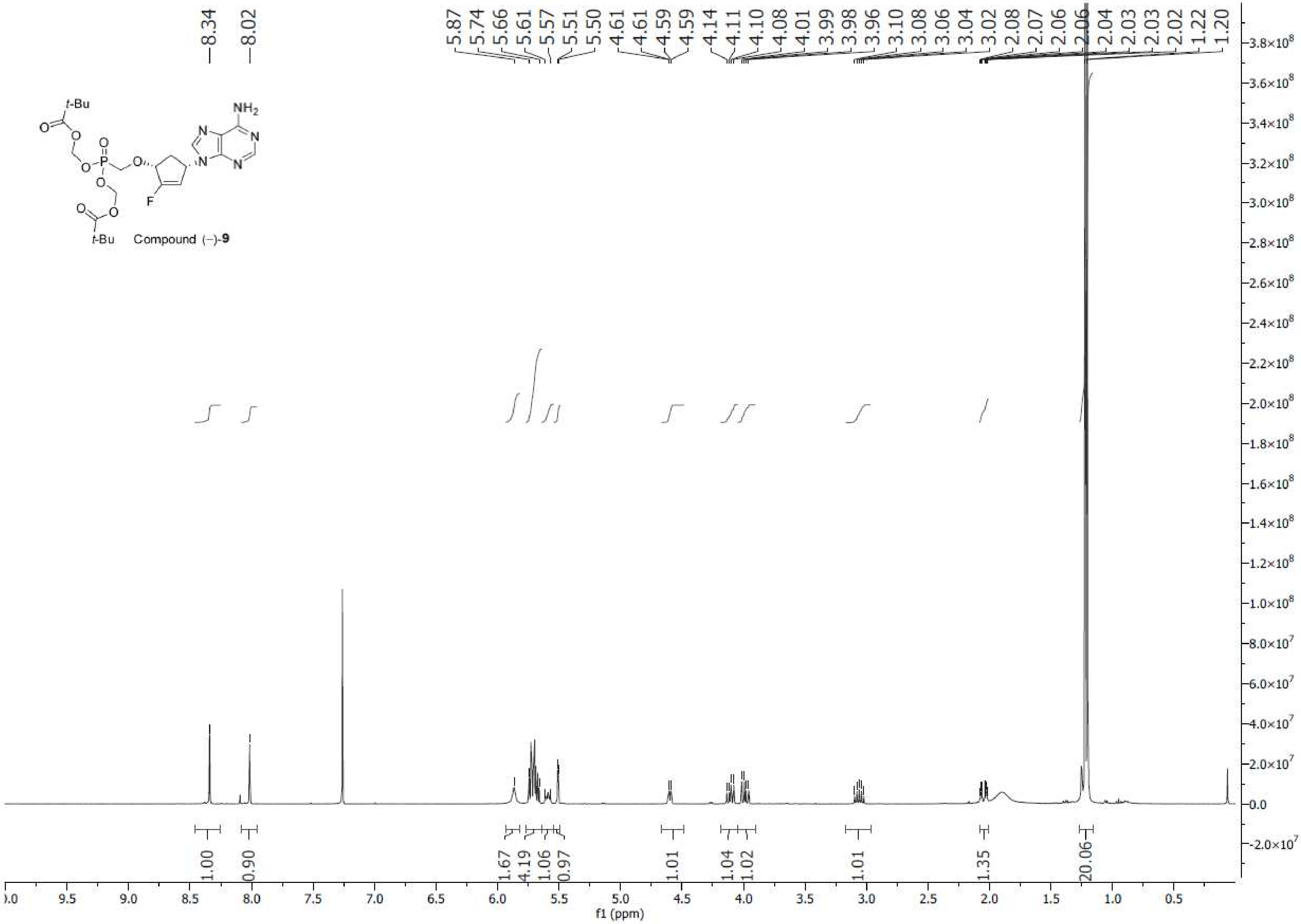

8.34
8.02
5.87
5.74
5.66
5.61
5.57
5.51
5.50
4.61
4.61
4.59
4.59
4.14
4.11
4.10
4.08
4.01
3.99
3.98
3.96
3.10
3.08
3.06
3.04
3.02
2.08
2.07
2.06
2.06
2.04
2.03
2.03
2.02
1.22
1.20
t-Bu
NH2
Compound (−)-9
1.00
0.90
1.67
4.19
1.06
0.97
1.01
1.04
1.02
1.01
1.35
20.06
f1 (ppm)

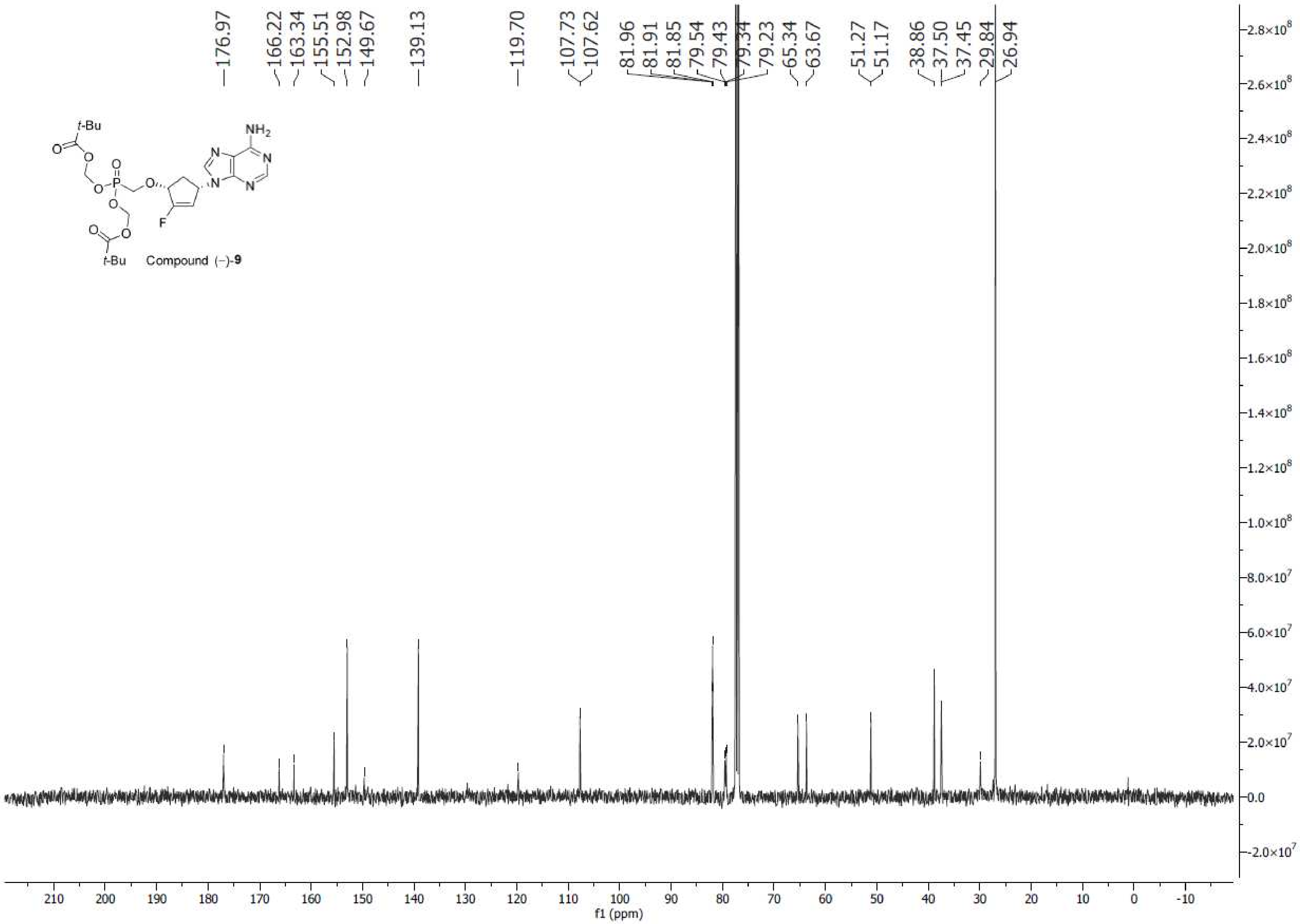
176.97
166.22
163.34
155.51
152.98
149.67
139.13
119.70
107.73
107.62
81.96
81.91
81.85
79.54
79.43
79.34
79.23
65.34
63.67
51.27
51.17
38.86
37.50
37.45
29.84
26.94
t-Bu
NH2
Compound (–)-9
f1 (ppm)

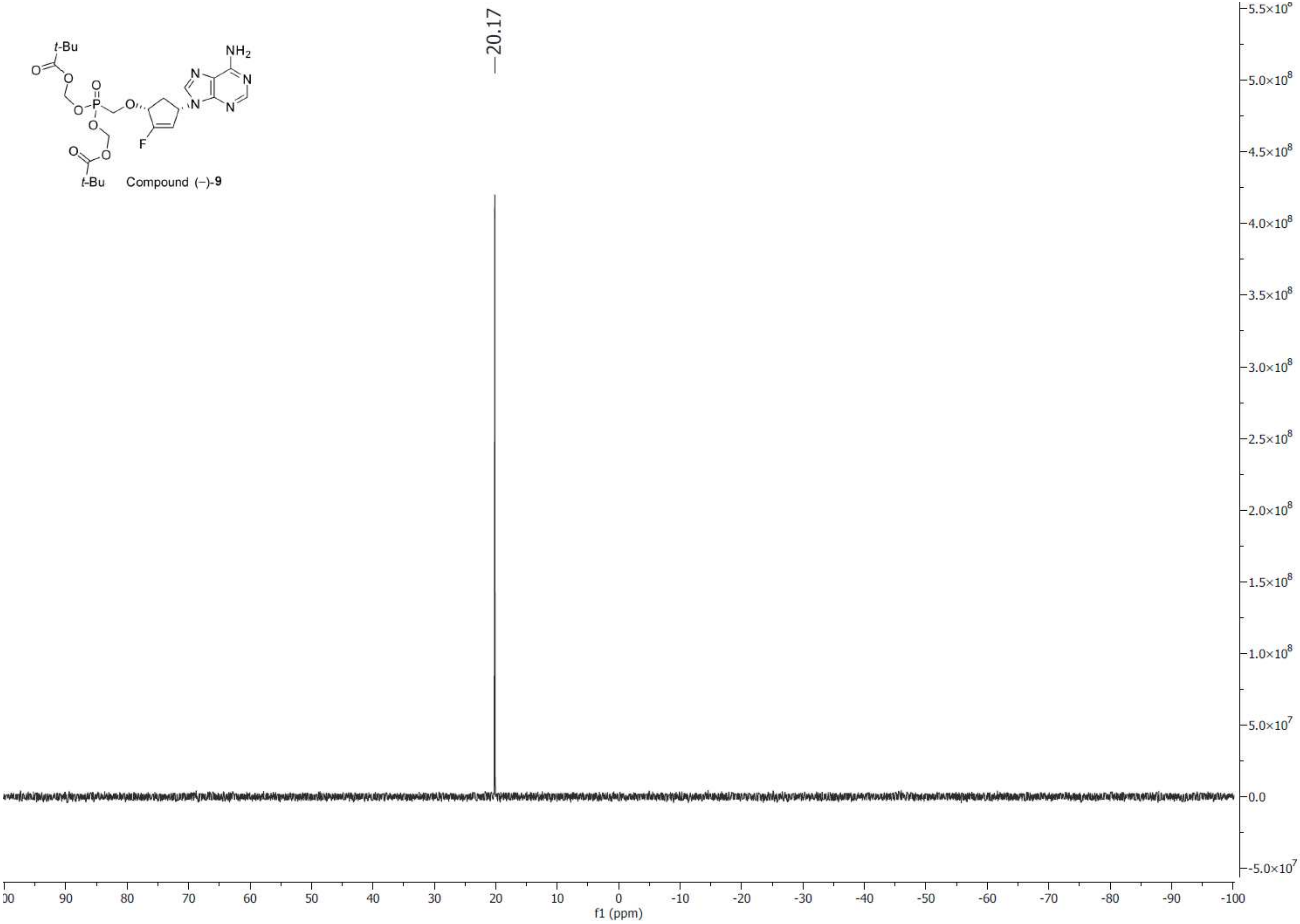
t-Bu
NH2
Compound (−)-9
20.17
f1 (ppm)
5.5×10^8
5.0×10^8
4.5×10^8
4.0×10^8
3.5×10^8
3.0×10^8
2.5×10^8
2.0×10^8
1.5×10^8
1.0×10^8
5.0×10^7
0.0
-5.0×10^7

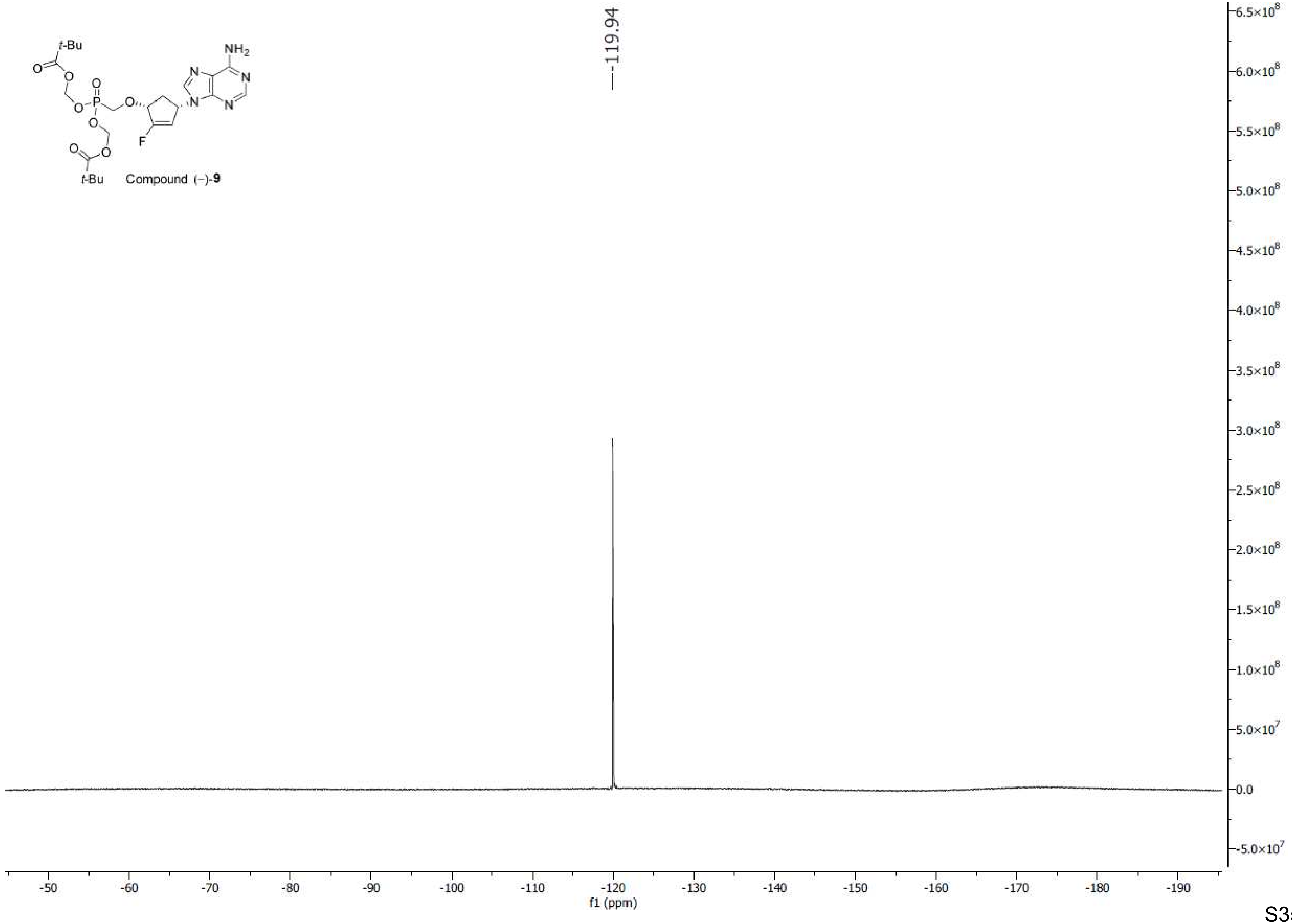

t-Bu
NH$_2$
F
t-Bu
Compound (–)-9
-119.94
f1 (ppm)

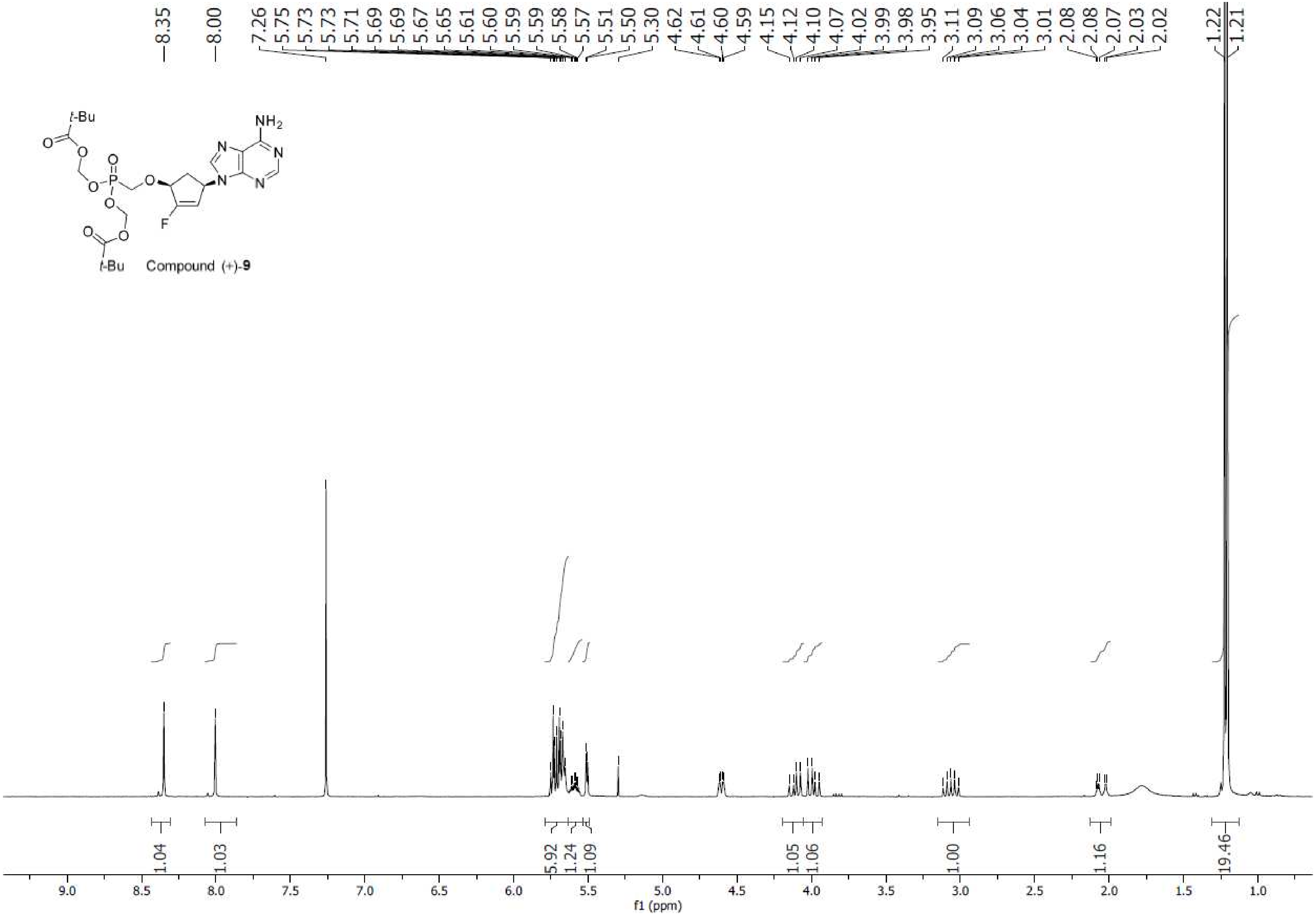

Compound (+)-**9**

Determination of enantiomeric excess by chiral HPLC

HPLC: Shimadzu LC20A, variable detector,

Column: chiralcel OD-H (150 x 4.6 mm x 5 µm), Mobile phase: hexane/iPrOH (98/2),

Flow rate: 1 ml/min, Temperature: 25°C, UV: 220 nm.

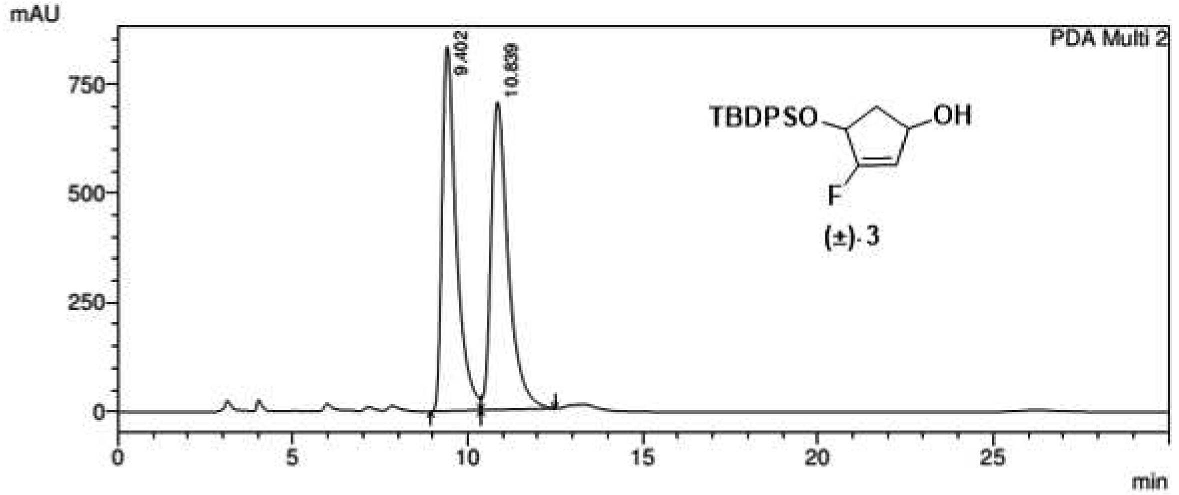

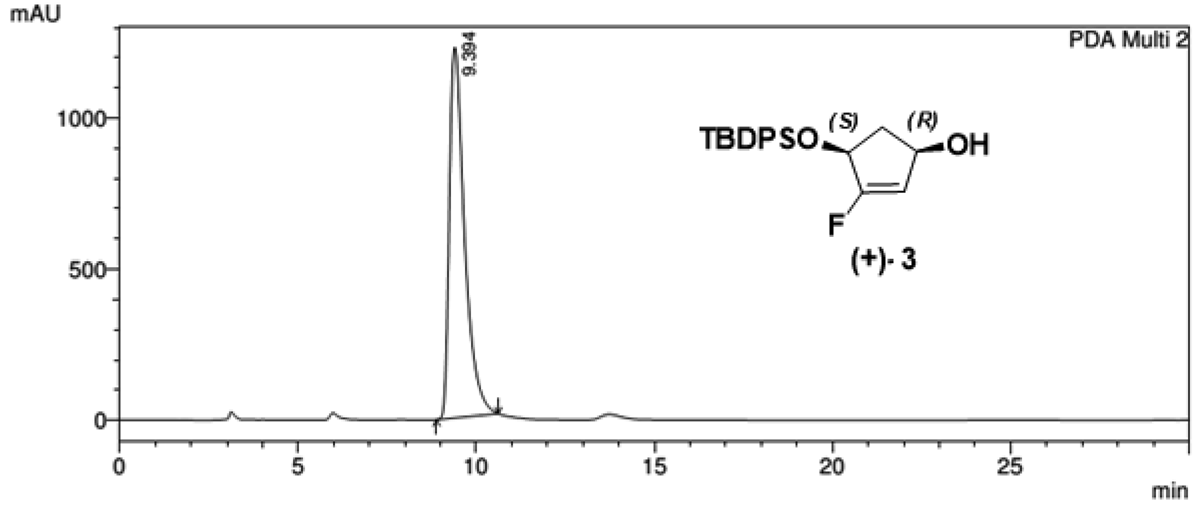

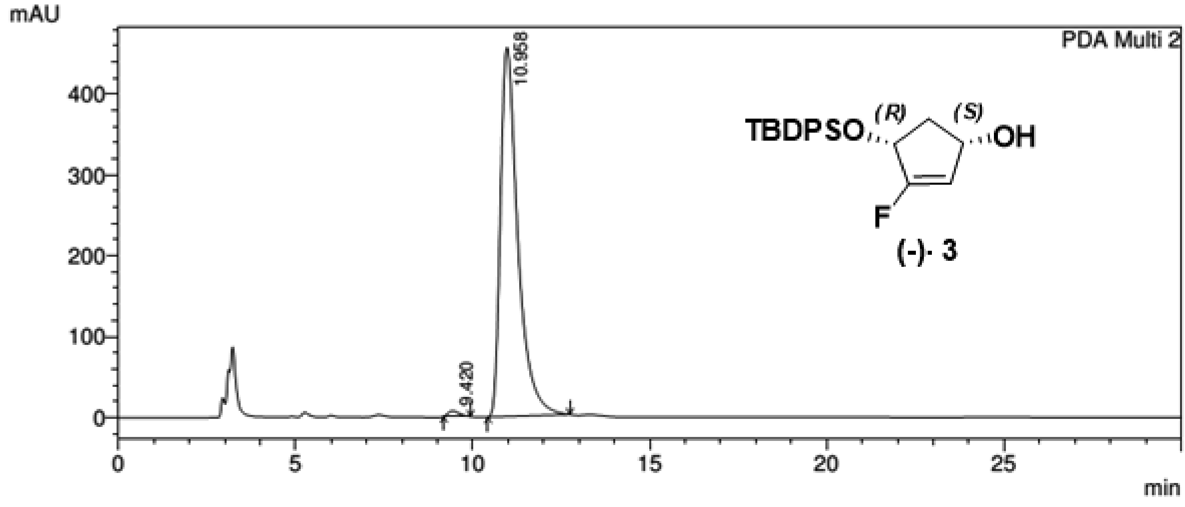

Supplement: Supplementary file 1 — Supporting Information [file CMDC-17-0-s001.pdf]
